# miR-155-5p drives coordinated tumor and macrophage reprogramming across multiple cancer entities

**DOI:** 10.1186/s12916-026-05146-7

**Published:** 2026-08-27

**Authors:** Theresa Kordaß, Ann-Katrin Schlosser, Marijan Czygan, Luisa Vieira Codeco Marques, Melisa Wartusch, Eric Nerenz, Vincencia Stefanie Muliawan, Stephan Kersting, Wolfram Osen, Stefan B. Eichmüller

**Affiliations:** 1 GMP & T Cell Therapy Unit, German Cancer Research Center (DKFZ), Heidelberg, Germany; 2 Department of General, Visceral, Thoracic, and Vascular Surgery, University Medicine Greifswald, Greifswald, Germany; 3 Institute of Pathology, University Hospital Mannheim, Mannheim, Germany; 4 Department of Pharmaceutical Biology, Institute of Pharmacy, Freie Universität Berlin, Berlin, Germany

**Keywords:** MicroRNA, Tumor microenvironment, Macrophage polarization, Immune checkpoint, CD73, PD-L1, Cancer immunotherapy, miR-155-5p, Tumor-immune interaction

## Abstract

**Background:**

T cell-based immunotherapies have improved cancer treatment, however their efficacy is frequently limited by tumor-intrinsic resistance mechanisms and by the immune suppressive tumor microenvironment, counteracting tumor specific immune responses through impaired antigen presentation and the accumulation of M2-like tumor-associated macrophages. MicroRNAs have emerged as regulators of tumor cell – immune cell interactions, yet the miRNAs’ capacity to coordinate anti-tumor effects across different cell types is poorly understood. This study aimed to investigate whether miR-155-5p and miR-3535 represent regulators capable of driving coordinated functional reprogramming of both, tumor cells and macrophages.

**Methods:**

Human tumor cell lines of various cancer entities were transfected with miR-155-5p or miR-3535 and the resulting effects on immune checkpoint molecule expression and tumor cell proliferation were assessed on transcriptional and functional level. Similarly, human PBMC-derived M2 polarized macrophages were transfected with the same microRNAs, followed by analysis of the cytokine secretion patterns and by transcriptomic profiling to evaluate the macrophage polarization states and the immune-regulatory pathways involved. Transcription factor activity and pathway enrichment analyses were performed to identify the regulatory mechanisms affected.

**Results:**

Both miR-155-5p and miR-3535 reduced expression of the immune checkpoint molecule CD73 (NT5E) in tumor cells, while miR-155-5p additionally suppressed PD-L1 (CD274) expression. At the same time, both microRNAs reprogrammed M2-like macrophages toward a pro-inflammatory M1-like phenotype, characterized by increased TNFα and CXCL10 secretion and the induction of M1-associated gene expression. Transcriptomic analysis revealed activation of STAT1/IRF-driven inflammatory pathways and a reduced activity of ZNF703, representing a transcriptional hub associated with M2 macrophage infiltration and poor prognosis. Both microRNAs increased TAP1 expression, suggesting enhanced antigen-processing capacity. Furthermore, miR-155-5p and miR-3535 exerted anti-proliferative effects across cell lines of various tumor entities.

**Conclusions:**

miR-155-5p and miR-3535 modulate tumor-intrinsic immune checkpoint expression and functional macrophage polarization in a coordinated way, linking tumor cell plasticity to neutralization of the immunosuppressive tumor environment. These findings highlight the potential of coordinated microRNA-mediated regulation via different cell types to overcome immune resistance mechanisms in tumors and support further investigation of microRNA-based strategies in cancer immunotherapy.

**Graphical abstract:**

**Figure:**
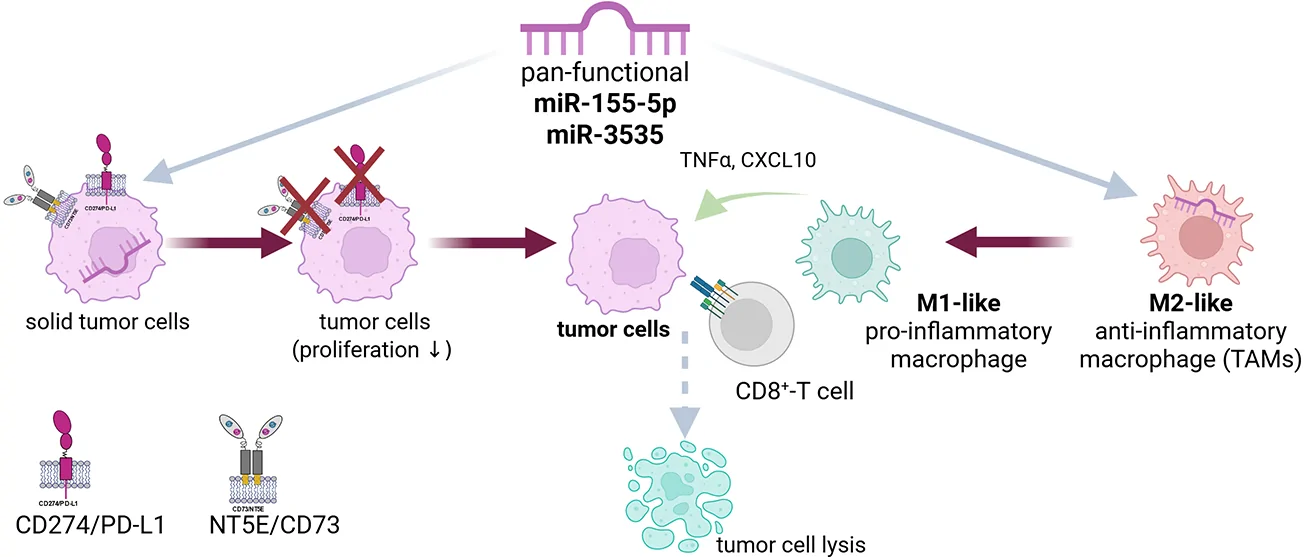

**Supplementary information:**

The online version contains supplementary material available at https://doi.org/10.1186/s12916-026-05146-7.

## Background

Current T cell-based immunotherapy strategies against tumors predominantly rely on the adoptive transfer of autologous tumor-reactive T cells, genetically engineered to express optimized tumor-specific T cell receptors or chimeric antigen receptors (CARs) [1]. Especially when combined with immune checkpoint blockade (ICB), such as PD-L1 inhibition, these approaches have shown the potential to significantly improve clinical outcomes across various tumor entities [2]. However, a substantial proportion of treated patients still fails to benefit from these therapies [3].

One major limitation to therapeutic efficacy is brought about by the immunosuppressive tumor microenvironment (TME), which is orchestrated by tumor cells in concert with a range of inhibitory immune cell populations — including regulatory T cells (Tregs), myeloid-derived suppressor cells (MDSCs), and tolerogenic dendritic cells (DCs) — that collectively antagonize tumor-reactive T cell activity [4]. Within this context, tumor-associated macrophages (TAMs) play a central role due to their pronounced functional plasticity, allowing them to shift dynamically between immunosuppressive M2-like and immunostimulatory M1-like phenotypes [5]. In most solid tumors, TAM populations are skewed toward the M2-like phenotype, a feature consistently linked to poor prognosis [6, 7].

While complete depletion of TAMs has been explored as a therapeutic approach [8] such strategies must be applied with caution, as M1-like macrophages can be critical mediators of antitumor immunity [9]. Consequently, a more promising approach may lie in selective repolarization of M2-like TAMs toward a proinflammatory, M1-like phenotype, to functionally neutralize the immunosuppressive milieu of the TME.

Tumor cells actively contribute to the establishment and maintenance of the TME by secretion of inhibitory cytokines such as TGF-β [10, 11], and additionally, through the expression of inhibitory checkpoint molecules like PD-L1 [12, 13]. Moreover, surface expression of the ectonucleotidases ENTPD1 (CD39) and NT5E (CD73) by tumor cells and regulatory immune cells promotes extracellular accumulation of adenosine, a potent immunosuppressive metabolite that impairs T cell function [14].

MicroRNAs (miRNAs) are small, non-coding RNAs that regulate gene expression post-transcriptionally by binding to the 3’ untranslated region (UTRs) of target mRNAs, thereby repressing mRNA translation [15]. A number of miRNAs have been implicated in macrophage polarization: for instance, miR-125-5p, miR-127, and miR-27a promote M1 polarization highlighted by upregulated expression of proinflammatory cytokines, whereas miR-23a, let-7c, miR-223a, and miR-511-3p support M2 polarization [16]. Moreover, on the tumor cell side, several miRNAs have been identified that modulate the expression of immune checkpoint molecules, including PD-L1 [17, 18].

In our previous work, we identified miRNAs that downregulate NT5E (CD73) expression in human melanoma and breast cancer cell lines [19, 20]. Building on these findings, we have now expanded our investigations to myeloid cells and report the identification of two miRNAs — miR-3535 and miR-155-5p — exerting complementary functions in distinct cell types, i.e. downregulated surface expression of immune checkpoint molecules (ICM) on tumor cells and M1-repolarization of M2-like macrophages.

MicroRNAs are inherently multi-target regulators and therefore often described as pleiotropic [21, 22]. Pleiotropy in this context refers to the regulation of diverse, sometimes functionally unrelated targets within a given cell type. Such pleiotropic effects can complicate therapeutic exploitation [23–25], and rarely result in coordinated cellular outcomes.In contrast, we propose the concept of pan-functional miRNAs as a distinct miRNA subclass. These miRNAs orchestrate coherent activities across different cell types within the tumor microenvironment, simultaneously acting on tumor-intrinsic resistance mechanisms and on immunoregulatory pathways in stromal or immune cells. By linking regulatory effects in tumor and imune cell compartments, pan-functional miRNAs may represent a novel class of immune-modulating tools that enable simultaneous reprogramming of multiple tumor-promoting mechanisms through a single miRNA species.

## Methods

All experiments were performed in accordance with institutional guidelines and standard laboratory procedures. Unless otherwise stated, experiments were conducted in at least three independent biological replicates. Detailed information on experimental design, reagents, and statistical analyses is provided below. Details of all antibodies, miRNAs, and macrophage inhibitors used in this study are provided in Additional file 1 Tables S1, S2, and S3, respectively.

### Cell lines and cell culture

All cell lines used in this study were cultured in RPMI medium supplemented with 10% FBS, without antibiotics at 37 °C and 5% CO_2_. Supernatants of cultured cells were regularly checked for mycoplasma contamination by PCR. DNA fingerprints were performed to confirm authenticity of human cell lines by the Forensic Medicine Department of the Heidelberg University Hospital (Heidelberg, Germany). Purchased cell lines were fingerprinted upon receipt. The breast cancer cell line MDA-MB-231, the colon cancer cell line HT-29 and the melanoma cell line SK-Mel-28 were purchased from ATCC. Further melanoma cell lines used in this study have been described elsewhere [26].

### Human monocyte isolation, macrophage differentiation, and polarization

Human buffy coats from healthy donors were obtained from the Blood Bank of Heidelberg University Hospital as anonymized surplus material. Peripheral blood mononuclear cells (PBMCs) were isolated by density gradient centrifugation using Ficoll-Paque. Residual erythrocytes were removed by red blood cell lysis. Monocytes were enriched by plastic adherence and cultured in RPMI medium supplemented with 10% fetal bovine serum.

Cells were seeded at a density of up to 5 × 10⁸ cells per 150 cm² dish and cultured for 9 days to allow differentiation into human monocyte-derived macrophages (HMDMs). Non-adherent cells were removed by repeated medium exchange on days 1, 2, 3, and 6.

For experimental assays, macrophages were detached using accutase or TrypLE, counted, and reseeded in 24-well plates (0.25 × 10⁶ cells/well) or 12-well plates (0.2 × 10⁶ cells/well), depending on downstream applications.

Macrophages were polarized for 24 h toward an M2-like phenotype using IL-4 (20 ng/mL) or toward an M1-like phenotype using IFNγ (20 ng/mL) alone or in combination with LPS (100 ng/mL).

### miRNA library screen

The human microRNA Mimic Version 21 library containing 2754 miRNAs was used to screen for miRNAs affecting surface expression of various immune checkpoint molecules in human cancer cell lines as described in our previous work [19]. Shortly, the breast cancer cell line MDA-MB-231 and the melanoma cell line SK-Mel-28 were transfected with the miRNA library and 72 h post transfection, cell surface expression of PDL1/CD274, NT5E/CD73 and ENTPD1/CD39 was measured by flow cytometry.

### RNA- and miRNA-Seq analysis

Human monocyte-derived macrophages (HMDMs) were polarized with either 20 ng/mL IL4 (anti-inflammatory M2-like), 20 ng/mL LPS + 20 ng/mL IFNγ or with 20 ng/mL IFNγ alone (pro-inflammatory M1-like) for 24 h. Untreated HMDMs are referred to as M0. Therefore, cells were seeded with a density of 2 × 10^5^ cells/well in 12-well plates. Total RNA was isolated from harvested cells using the miReasy kit (Qiagen, Hilden, Germany). Whole RNA and small RNA sequencing of HMDMs were conducted by GENEWIZ Multiomics & Synthesis Solutions from Azenta Life Sciences, using an Illumina HiSeq, PE 2 × 150 Sequencing System. GENEWIZ was responsible for library preparation, sequencing, and the subsequent bioinformatics analysis. This analysis involved trimming sequence reads to eliminate potential adapter sequences and low-quality nucleotides with Trimmomatic v.0.36. The trimmed reads were then aligned to the Homo sapiens GRCh38 reference genome, using the STAR aligner v.2.5.2b, For the quantification of gene expression, unique gene hit counts were calculated using featureCounts from the Subread package v.1.5.2, based on the ’gene_id’ feature in the annotation file, and counting only unique reads that mapped to exon regions. Differential gene expression analysis was carried out on the obtained gene hit counts using DESeq2. This involved comparing gene expression between predefined groups of samples, with the Wald test being utilized to calculate p-values and log_2_ fold changes. For small RNA sequencing data, read counts were computed using customized scripts. Genes and miRNAs were identified as differentially expressed based on criteria of an adjusted p-value < 0.05 and a log_2_ fold change > 1 for each comparison. The processed sequencing datasets have been uploaded as supplementary Additional files. Additional file 2 contains the normalized HMDM RNA-sequencing dataset, and Additional file 3 contains the processed human miRNA sequencing dataset.

### Microarray analysis

Gene expression profiling on miRNA-repolarized macrophages was performed as follows. HMDMs were seeded at a density of 4 × 10^5^ cells/well in 6-well plates. Cells were then treated with 20 ng/mL IL4. After 24 h, polarized cells were transfected with 50 nM miR-155-5p, miR-9-5p, miR-3535 or ath-miR-416 (control miRNA, respectively) using Dharmafect 4 Transfection reagent (Horizon Discovery, Cambridge, UK) as described in more detail in the Additional file 1 within Supplementary Methods. After 48 h, cells were treated with accutase and gently harvested with a cell scraper and used for RNA isolation (RNAeasy kit, Qiagen, Hilden, Germany). Microarrays as well as raw data processing were performed by the DKFZ - Genomics and Proteomics Core Facility using an Affymetrix Clariom S human chip for all samples. Differential gene expression was calculated by comparison of miRNA-induced gene expression levels to the respective gene expression levels following control miRNA transfection. Data analysis was performed using R Version 4.4.3 and RStudio Version 2024.12.1. The processed dataset has been uploaded as a Additional file 4, including analyses for each pan-miRNA and corresponding GSEA results.

### Live cell imaging

10,000 tumor cells/well were seeded in 96-well plates, rested for 24 h and subsequently transfected with 50 nM miRNA (0 h) using RNAi Max transfection reagent. Cell confluence was analyzed with Live-cell imaging using the IncuCyte SX5 system over a period of 70 h with images captured every 2 h. Cell confluence and proliferation dynamics were analyzed using the IncuCyte 2020B software.

## Results

### Multiple regulators jointly control repolarization of anti-inflammatory macrophages

Tumor-associated macrophages are key drivers of the immunosuppressive TME. To characterize molecular determinants of macrophage polarization, we compared the transcriptional landscape of pro-inflammatory and anti-inflammatory macrophages. Therefore, human monocytes were isolated from buffy coats of healthy blood donors and polarized with IL4 towards an anti-inflammatory (M2-like) or a pro-inflammatory phenotype using IFNγ or LPS + IFNγ (M1-like), respectively. RNA sequencing data identified classical marker genes allowing discrimination between different macrophage polarization states. M1-like macrophages displayed a distinct transcriptional profile, whereas M0 and M2-like macrophages clustered more closely together (Additional file 1: Fig. S1A, B). Based on differential gene expression analysis we selected CXCL10, CXCL9, TNF, CCL5, TAP1, UBD, JAK3, STAT2 and IL12B as M1-marker genes and MRC1, PPARG, TREM2, CCL22 and CCL18 as M2-marker genes (Additional file 1: Fig. S1 C, D).

**Fig. 1:**
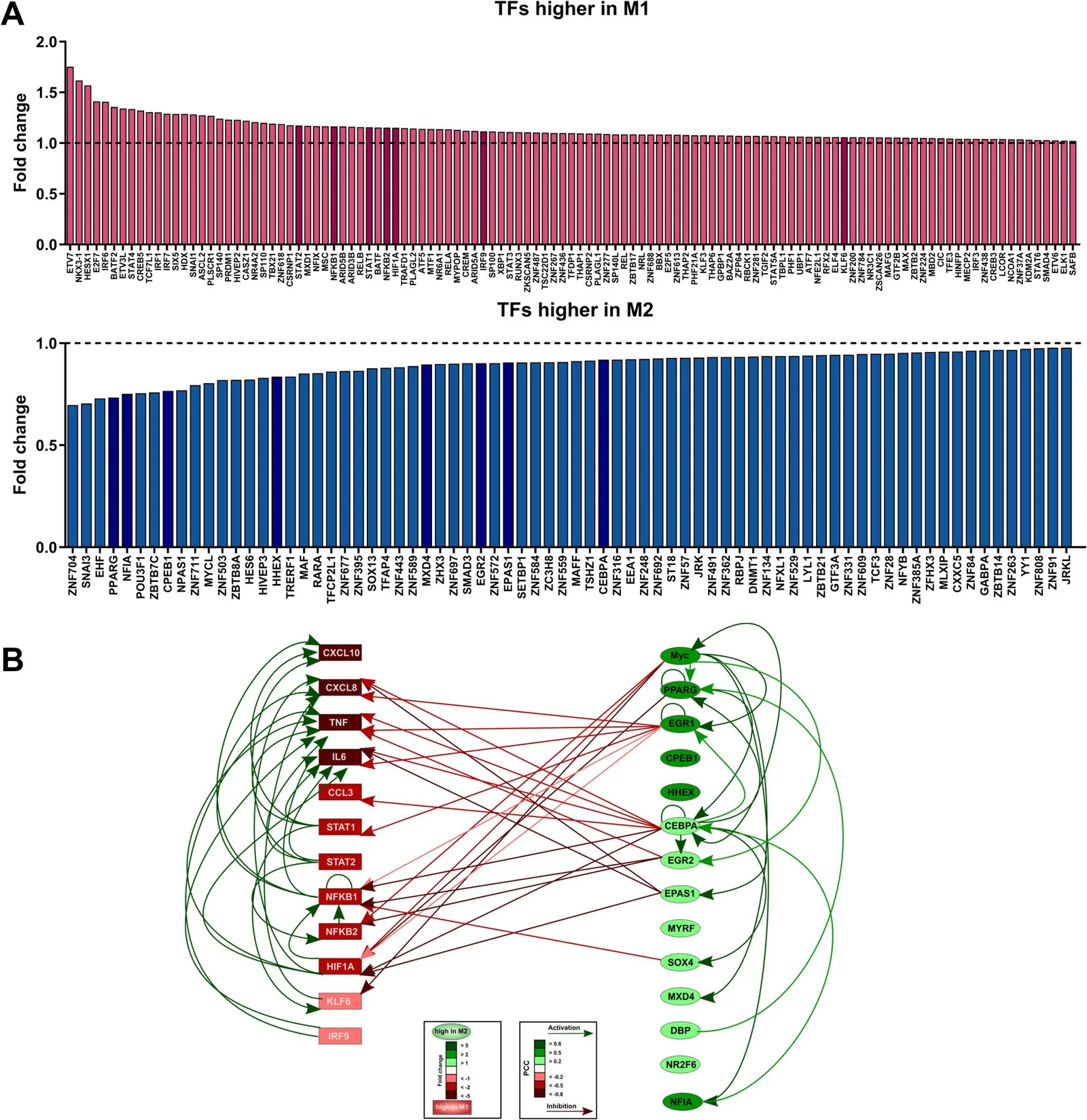
Human macrophages exhibit distinct transcription factor profiles following M1 or M2 polarization. Human monocyte-derived macrophages (HMDMs) derived from three healthy donors were polarized with IL4 into M2-like, or with LPS + IFNγ into M1-like macrophages, respectively and used for mRNA sequencing. Three biological replicates per condition were analyzed. **A**: Transcription factors (TFs) were ranked based on fold expression comparing M1- and M2-like HMDMs and a significant difference (p<0.05) between the two groups (magenta: higher expressed TFs in M1; blue: higher expressed in TFs in M2). **B**: Based on the sequencing data, known TF-target gene interactions and the respective correlation of TF and target gene across all samples (n = 9), a network was constructed showing the interconnection of the differentially expressed TFs as well as classical macrophage polarization marker genes.

**Fig. 2:**
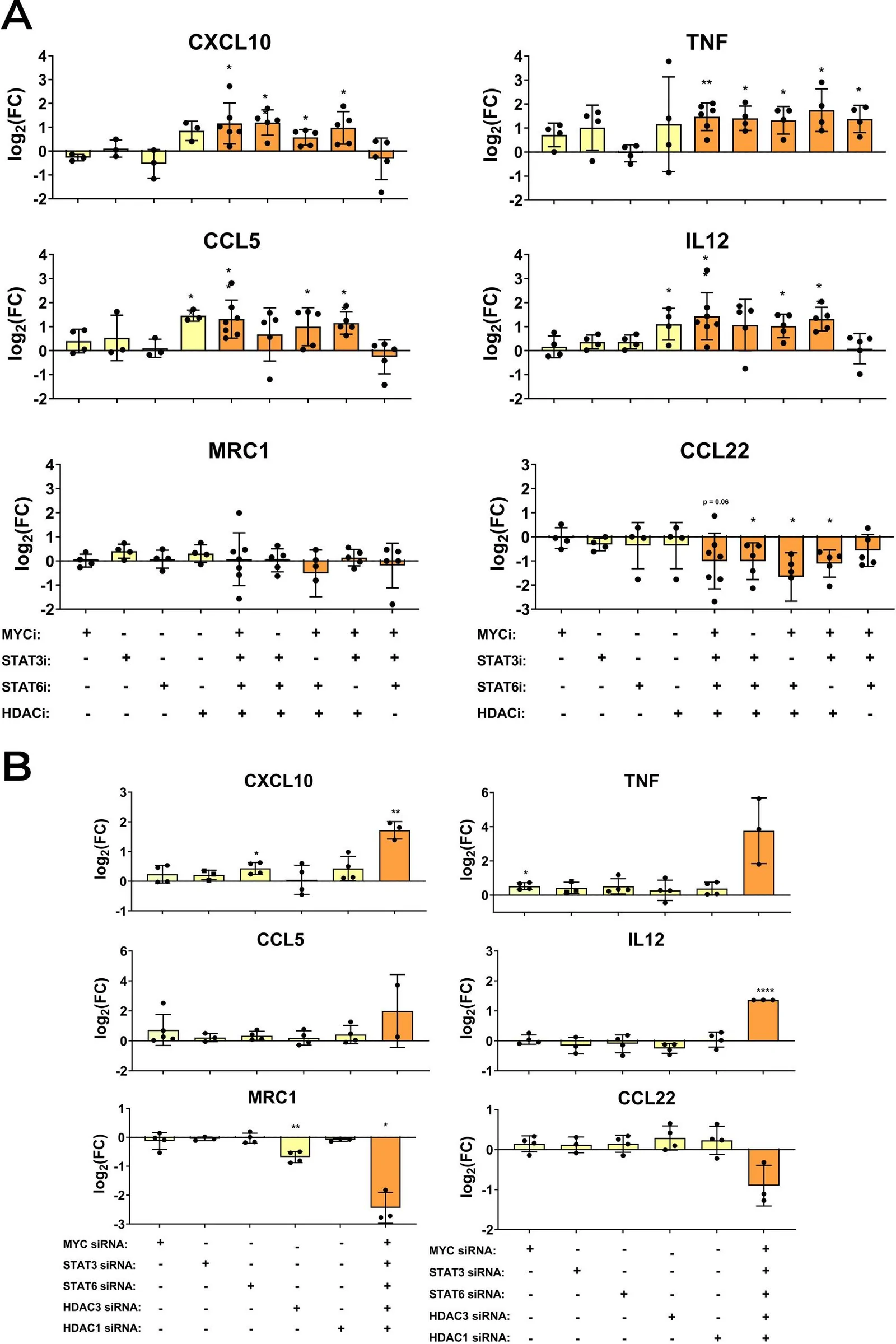
Combined inhibition of M2-associated regulators induces repolarization of M2-like macrophages. HMDMs derived from healthy donors were polarized into M2-like macrophages upon IL4 treatment for 24 h and subsequently treated with A small molecule inhibitors targeting M2-associated regulators (MYC, STAT3, STAT6, HDAC), or B siRNAs directed against the mRNAs of the regulators tested in A. After 48 h, macrophages were harvested, mRNA was isolated, and the expression of representative M1 (CXCL10, TNF, CCL5, IL12) and M2 (MRC1, CCL22) marker genes was quantified by qPCR. RPL19 served as housekeeping gene. Fold changes (FC) were calculated relative to corresponding controls. Individual dots represent donor-derived biological replicates; data are presented as mean ± SD. Yellow bars represent single-agent treatments, whereas orange bars indicate treatment with pooled inhibitors (**A**) or siRNAs (**B**), respectively. Significance was determined using a one-sample t-test. *p < 0.05; **p < 0.01; ***p < 0.001; ****p < 0.0001. Treatments applied in each condition are indicated by “+” or “–” below the figure; the inhibitors with their respective used concentrations are listed in Additional file 1 in Supplementary Table S3 [27–32]. No overt cytotoxic effects were observed under the applied inhibitor conditions, as assessed by cell morphology, adherence, and cell density (data not shown).

We next identified transcription factors (TFs) selectively associated with pro- or anti-inflammatory macrophage phenotypes (Figure 1A) and constructed a gene regulatory network incorporating cytokines and transcriptional regulators (Figure 1B). Upon correlation of expression levels based on known TF-target gene interactions we observed that many M2-associated TFs exhibited a negative correlation with M1-associated TFs and effector genes like MYC, EPAS1 or EGR1. Thus, we hypothesized that silencing of these highly interconnected M2-associated regulators could shift macrophages towards the M1-like, pro-inflammatory phenotype. While the pool of inhibitors targeting the M2-associated transcription factors HDAC, MYC, STAT3, and STAT6 increased the mRNA expression of the M1-like markers CXCL10, TNF, and IL12 in HMDMs, individual inhibition of these TFs failed to do so (Figure 2A). Notably, HDAC was the only transcription factor, whose sole inhibition significantly enhanced the expression of two M1 markers, CCL5 and IL12. Besides inhibitors blocking the TF function on protein level, we also tested siRNA molecules targeting the respective TF-encoding mRNA molecules (Figure 2B). In concordance with the data obtained with TF inhibitors, application of pooled siRNAs targeting all TFs (MYC, STAT3/6, HDAC1/3) significantly increased the expression of the M1-markers CXCL10, TNF and IL12, while the expression of the M2-marker MRC1 was significantly reduced. Targeting TFs with individual siRNAs resulted only in limited M1 repolarization. A minor effect was observed upon HDAC3 knockdown, which modestly decreased MRC1 transcript levels.

### miR-9-5p, miR-155-5p and miR-3535 induce a pro-inflammatory gene expression program in human macrophages

**Fig. 3:**
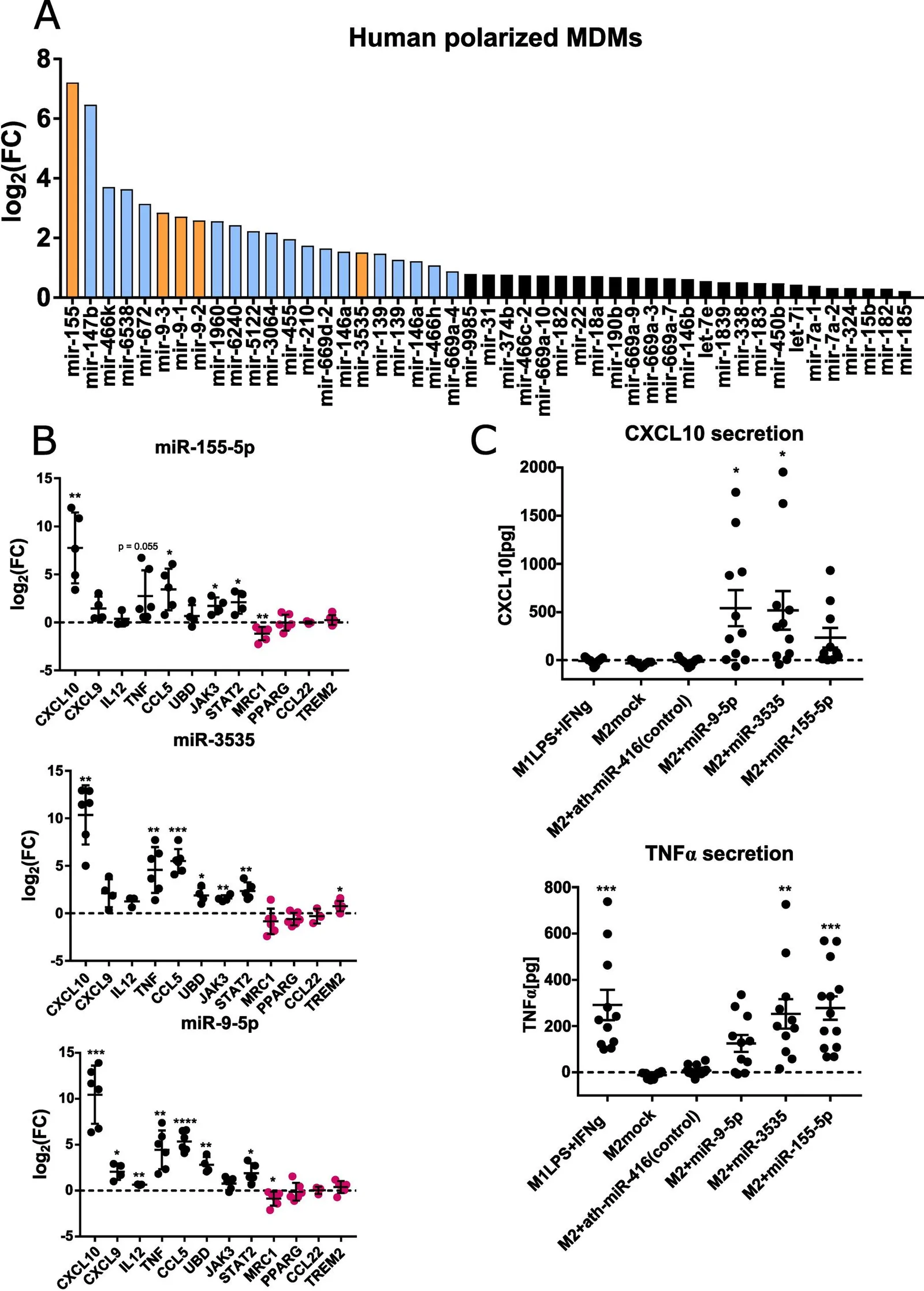
miR-155-5p, miR-3535, and miR-9-5p are enriched in pro-inflammatory macrophages and promote repolarization of anti-inflammatory macrophages. **A**: HMDMs were polarized with either IFNγ+LPS towards M1-like or with IL4 towards M2-like state. After 24 h cells were subjected to miRNA-sequencing. Three replicates were performed per condition. Expression of miRNAs was compared among M1- and M2-like macrophages and only miRNAs significantly (p < 0.05) up-regulated in M1-like cells are depicted. miRNAs with a log2(FC) > 1 are depicted in color and miRNAs selected for further validation are highlighted in orange. **B**: HMDMs were polarized towards M2-like state with IL4 for 24 h. Cells were then transfected with 50 nM miRNA and expression of classical M1(black) and M2(magenta) genes was quantified by qPCR 48 h post transfection. RPL19 was used as housekeeping gene. Fold changes (FC) were calculated relative to respective miRNA control (ath-miR-416) transfected M2-like cells. **C**: HMDMs were polarized with IL4 for 24 h in 6-well plates and transfected with 50 nM miRNA. Supernatant was collected 48 h post transfection and used for quantification of the M1 associated effector cytokines CXCL10 and TNFα by ELISA. Mean ± SD are shown. Each dot represents an independent experiment. Significance was assessed by one-sample T-test. *: p < 0.05; **: p < 0.01; ***: p < 0.001; ****: p < 0.0001.

Our data indicated that inhibition of individual regulators alone is insufficient to induce robust macrophage repolarization. We therefore explored the use of microRNAs for macrophage reprogramming, given their capacity to coordinately regulate multiple target genes. Macrophages display distinct microRNA expression profiles depending on their polarization state, and of note, several microRNAs have previously been implicated in macrophage reprogramming [17, 33, 34]. We focused on miRNAs significantly up-regulated in M1-like macrophages that might promote M1-directed repolarization. Among 22 miRNAs upregulated in M1 macrophages, miR-155-5p, miR-9-5p, and miR-3535 were more abundant in M1- than in M2-like macrophages, with miR-155-5p showing the highest expression (Fig. 3A, Additional file 1: Fig. S2A). To identify candidate miRNAs of M1-repolarization, we predicted target genes [35–44] of the 22 miRNAs that were found to be upregulated in M1-like macrophages. The predicted target genes were subsequently filtered for candidates with elevated expression in M2-like macrophages, yielding a set of 24 genes (Table 1). For each miRNA, the number of potential binding sites within their 3’-UTR for the respective mRNA target molecule was calculated (see Additional file 5). Among the 22 miRNAs analyzed, miR-155-5p had the highest number of predicted M2 targets with (16/24), followed by miR-3535 predicted to harbor binding sites for 11/24 M2 targets. miR-155-5p, miR-9-5p and miR-3535 were selected for further analysis, since these miRNAs were also determined to target multiple M2-regulatory genes (Table 1, Additional file 1: Fig. S2). Importantly, qPCR-based validation confirmed endogenous expression of miR-155-5p and miR-3535 in macrophage polarization models (Additional file 1: Fig. S3). Both miRNAs were enriched in M1-polarized THP1-derived macrophages compared to M0- and M2-like conditions. While miR-155-5p expression was below the detection limit in MDA-MB-231 tumor cells, miR-3535 was readily detectable in both THP1-derived macrophages and MDA-MB-231 cells. Notably, miR-3535 has not been characterized as a human miRNA in standard annotation databases yet, as it maps to a snoRNA-associated genomic locus (Additional file 1: Fig. S4). Nevertheless, its reproducible detection by qPCR in both macrophages and tumor cells supports the presence of this small RNA species in human cellular systems and further motivated its functional characterization in the present study.

**Table 1:** miR-155-5p and miR-3535 are predicted to target classical M2-associated signature genes. MiRNAs significantly upregulated in M1-like macrophages were analyzed for predicted target genes. Candidate targets were filtered for genes showing significantly higher expression in M2-like macrophages based on RNA sequencing data from polarized human monocyte-derived macrophages. Predicted miRNA binding sites were obtained using miRmap. FC, fold change in gene expression comparing M1- versus M2-like polarized macrophages

M2 gene	FC M1 vs. M2	miR-155-5p	miR-3535	miR-9-5p
MRC1	0.65	1	0	0
ADCY1	0.65	1	1	1
CD93	0.66	2	3	1
ZNF704	0.70	2	7	2
AKAP5	0.61	1	0	0
PARM1	0.58	0	4	0
FGD5	0.69	1	0	1
PLXNA2	0.67	1	1	2
CD300LB	0.69	1	1	2
CD200R1	0.63	0	1	0
CXCR1	0.67	1	0	0
TACSTD2	0.68	0	0	0
F13A1	0.50	2	2	0
PLXDC1	0.58	1	0	0
TMEM236	0.60	1	1	0
GPR34	0.65	1	0	1
RASL11B	0.67	1	0	0
ITGA11	0.61	0	0	0
CD180	0.65	0	0	0
PNPLA3	0.62	1	0	0
DACT1	0.66	0	0	0
CLEC4A	0.68	0	0	0
ADAMTS15	0.57	0	3	0
HOMER2	0.70	1	1	0
Sum		16/24	11/24	7/24

Next, we tested the capacity of miR-9-5, miR-155-5p and miR-3535 to repolarize anti-inflammatory macrophages. Quantitative PCR analysis revealed significantly upregulated gene expression of the M1-associated markers CXCL10, TNFα, CCL5 and UBD as well as the M1 associated TF STAT2 in HMDMs transfected with miR-3535 or miR-9-5p (Fig. 3B, middle and bottom). miR-155-5p induced the same gene expression pattern, although the increased expression levels of TNFα and UBD failed to reach significance (Fig. 3B, top). Of note, in HMDMs, transfection with miR-9-5p and miR-155-5p reduced MRC1 expression to a significant extent, while expression of the other M2 associated marker genes or TFs remained unchanged, with the exception of TREM2, whose expression was slightly enhanced upon transfection with miR-3535.

We next quantified the amount of secreted CXCL10 and TNF as representative M1-associated cytokines in M2-polarized HMDMs following transfection with miR-9-5p, miR-3535, or miR-155-5p. All three miRNAs induced secretion of CXCL10 and TNF, albeit the increase in secretion levels of TNF and CXCl10 following transfection of miR-9-5p or miR-155-5p, respectively, failed to reach significance (Fig. 3C). Notably, inter-donor variability was observed in the magnitude of miRNA-induced responses. While all donors showed a consistent shift toward a pro-inflammatory phenotype, the extent of cytokine induction (e.g., TNFα, CXCL10) and gene expression changes varied between donors.

Additionally, we tested the effect of miR-155-5p and miR-3535 on the surface expression of the M1-markers CD80/CD38 and the M2-marker CD209 by flow cytometry 72 h after miRNA transfection (see Additional file 1: Fig.S5). Both, miR-155-5p and miR-3535 strongly enhanced the proportion of CD80^+^ and CD38^+^ cells compared to control transfectants. Furthermore, the percentage of CD209^+^ cells was reduced upon miR-155-5p and miR-3535 transfection to levels comparable to those observed with LPS/IFNγ-polarized cells. Together, these findings indicate that a single miRNA can broaden the macrophage repolarization signatures beyond those achieved by individual inhibition of M2-associated regulators, supporting the concept that macrophage polarization is governed by a complex regulatory network that can be coordinately modulated by particular miRNAs.

### Pan-functional miRNAs inhibit immune checkpoint molecule expression in tumor cells

**Fig. 4:**
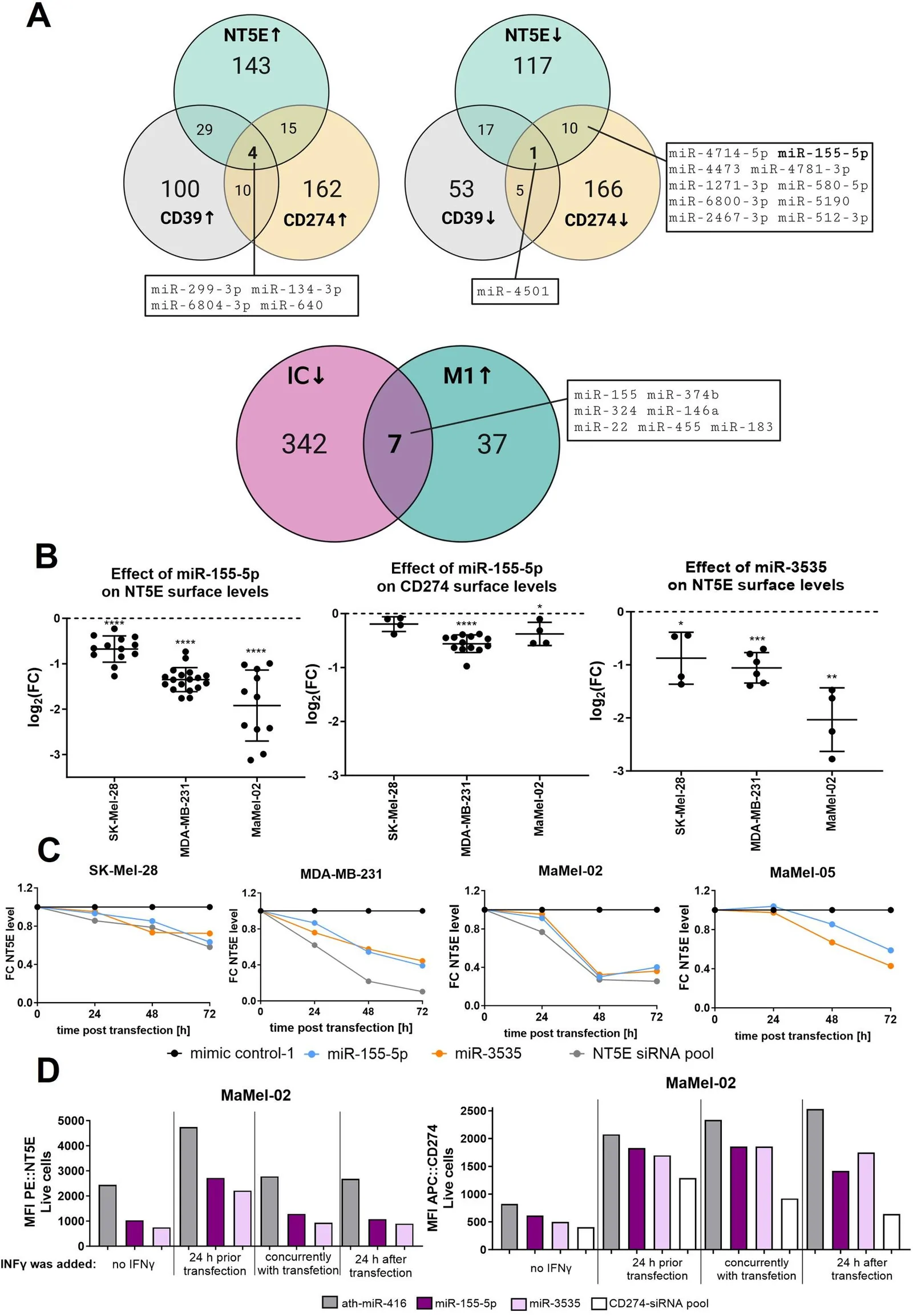
miR-155-5p and miR-3535 exert coordinated effects on immune checkpoint regulation across tumor cell lines and macrophages. **A**: Venn diagram depicting shared miRNAs mediating enhanced or reduced ICM expression in human tumor cell lines (A, top) as well as pan-functional miRNAs inducing both reduced ICM expression as well as M1 polarization of M2-like macrophages (A, bottom). **B**: pan-functional miR-155-5p and miR-3535 suppress NT5E and CD274 surface expression on human cell lines of various tumor entities. Cells were transfected with 50 nM miRNA and cell surface expression of NT5E and CD274 was analyzed by flow cytometry 48 h post transfection. Fold changes (FC) were calculated relative to respective mimic control transfectants (ath-miR-416). Each dot represents an independent experiment. Mean ± SD are shown. Significance was assessed by one-sample T-test. *: p < 0.05; **: p < 0.01; ***: p < 0.001; ****: p < 0.0001. **C**: Kinetics of the inhibitory effect of miR-155-5p and miR-3535 on NT5E surface expression levels in human tumor cell lines. Cells were transfected with 25 nM miRNA or siRNA pool followed by flow cytometric quantification of NT5E surface expression 0, 24, 48 and 72 h post transfection. Median fluorescence intensity (MFI) of NT5E surface expression was compared to the MFI of mimic control-1 transfectants (ath-miR-416). **D**: The effect of IFNγ on miRNA-inhibited NT5E and CD274 surface expression was tested on MaMel-02 cells by addition of 100 ng IFNγ 24 h prior, concurrently with or 24 h after transfection of miR-155-5p or miR-3535, respectively. Transfection with CD274 targeting siRNA pools was used as control. Cell surface levels of NT5E and CD274 were measured 48 h after transfection.

We next asked whether miR-155-5p, miR-3535 and miR-9-5p would exert functional effects on tumor cells and modulate cell surface expression of immune checkpoint molecules. Moreover, to identify miRNAs with pan-functional activity, i.e. miRNAs inducing anti-tumoral activity in different cell types, we compared the results of a previous miRNA library screen on miRNAs affecting the expression of the immune checkpoint molecules (ICMs) NT5E/CD73, CD274 and CD39 on tumor cells [19], with our miRNA sequencing data of M1-like macrophages. miR-4501 turned out as the only miRNA capable of inhibiting all three ICMs. Conversely, miR-134-3p, miR-229-3p, miR-6804-3p and miR-640 consistently enhanced the expression of these ICMs (Fig. 4A top). miR-155-5p emerged as one of ten miRNAs inhibiting surface expression of NT5E/CD73 and PD-L1/CD274 on human tumor cell lines. In addition, miR-155-5p was strongly expressed in M1-like macrophages (Fig. 4A top and bottom). Moreover, further miRNAs were identified that inhibited expression of at least one ICM and whose expression was upregulated in M1-like macrophages, including miR-22, miR-146a, miR-183, and miR-324 (Fig. 4A bottom).

We then selected miR-155-5p and miR-3535 as pan-functional miRNAs for further characterization. miR-3535 maps to a human small nucleolar RNA locus (Additional file 1: Fig. S4) and is predicted to contain multiple seed sequences for binding sites within the 3‘UTR of NT5E and CD274 specific target mRNAs. Because miR-3535 has not yet been annotated in standard human microRNA libraries, it was initially not included in the overlap analysis. However, given its strong upregulation in sequencing data from human M1-like macrophages, we evaluated its potential to regulate immune checkpoint molecule expression. Both miR-155-5p and miR-3535 reduced NT5E surface expression in various human tumor cell lines (Fig. 4B), and this effect was confirmed at the mRNA level (Fig. 4C). Thus, the observed effects were supported by both transcript-level analyses and flow cytometric quantification of cell-surface protein expression, which is particularly relevant for membrane-associated immune checkpoint molecules. Dose–response experiments in MDA-MB-231 cells revealed that both miR-155-5p and miR-3535 reduced NT5E and CD274 expression across all concentrations tested. While miR-3535 mediated increasing effects with higher concentrations, miR-155-5p induced a non-linear response with maximal NT5E suppression at 25 nM, while a plateau in CD274 repression was reached with 25 nM onwards (see Additional file 1: Fig. S6).

In most tumor cell lines tested, the miRNA-mediated inhibitory effect reached its maximum 72 h after transfection. Since IFNγ activates CD274 expression [45, 46], we tested whether miRNA-155-p and miR-3535 mediated inhibition of CD274 and NT5E expression is still preserved in the presence of this cytokine. Thus, changes in NT5E and CD274 cell surface expression levels of miRNA transfected MaMel-02 cells treated with IFNγ at various time points before or after transfection were analyzed by flow cytometry (Fig. 4D). Treatment with IFNγ alone had a stimulatory effect on surface expression levels of CD274. Notably, NT5E surface levels were only increased if IFNγ was supplied prior to miRNA transfection. Regardless of the timepoint of IFNγ application, both miRNAs were capable to inhibit the surface expression of both NT5E and CD274. Interestingly, miRNA mediated inhibition of CD274 surface expression was strongest, when IFNγ was added 24 h after the transfection. This applied also for the inhibition of CD274 expression by the siRNA pool.

### miR-155-5p, miR-3535, and miR-9-5p exert anti-proliferative effects in human tumor cell lines

**Fig. 5:**
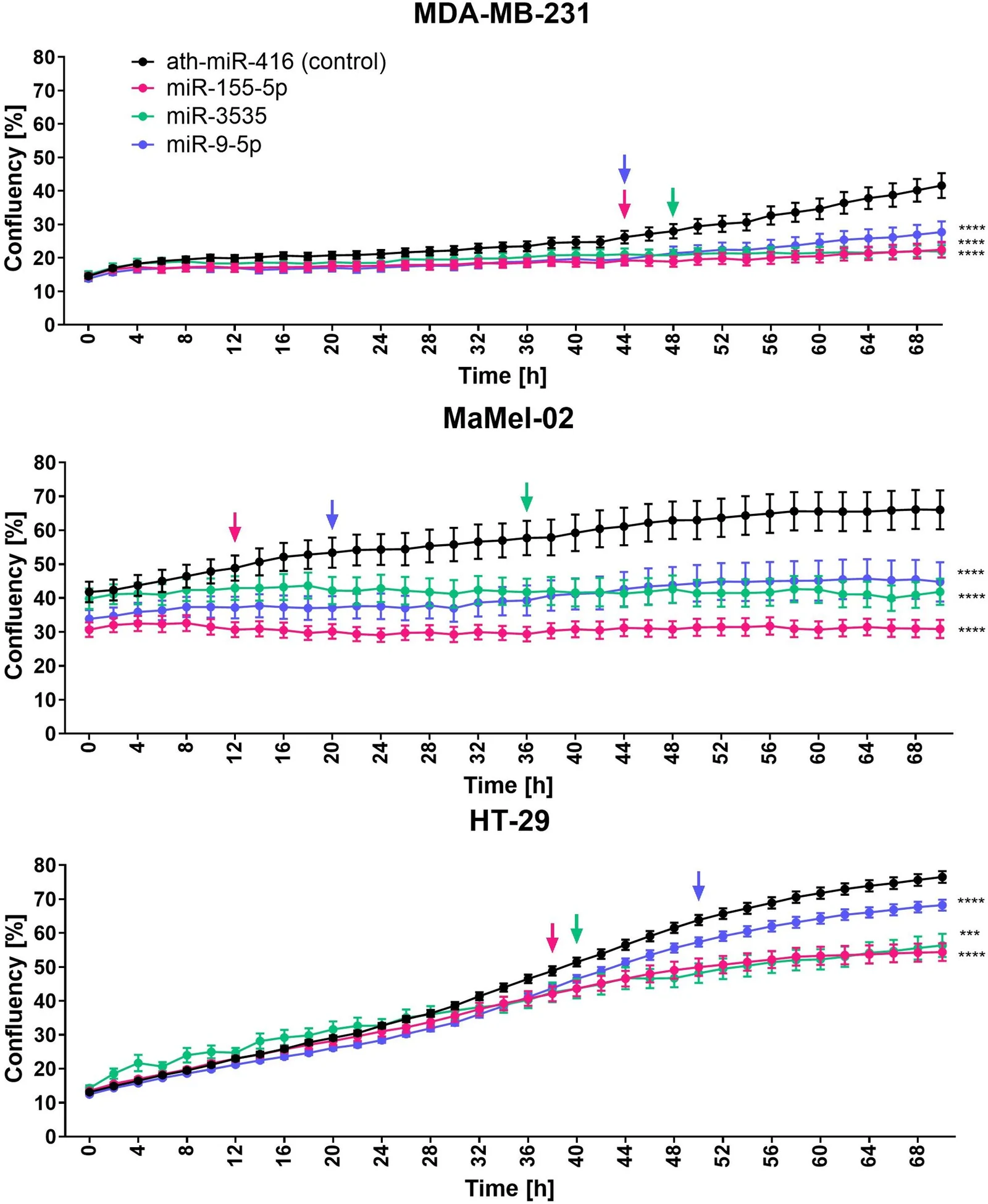
miR-155-5p, miR-3535, and miR-9-5p inhibit proliferation of human cancer cell lines across different tumor entities. Tumor cells (10,000 cells per well) seeded in 96-well plates were transfected 24 h later with 50 nM microRNA (0 h). Confluence was monitored by live-cell imaging using the IncuCyte SX5 system for 70 h, with images captured every 2 h. Confluence and proliferation kinetics were analyzed using IncuCyte 2020B software. Proliferation of miRNA-transfected cells was compared to cells transfected with control miRNA ath-miR-416 (black). Mean ± SD are shown. For each cell line and condition 4 replicates were performed. For control transfections 5 replicates were performed. Statistical significance was assessed by two-way ANOVA followed by multiple comparison testing comparing each miRNA-treated condition with the corresponding ath-miR-416 control at each time point. Arrows indicate the first time point at which statistically significant differences versus control miRNA were detected. In most cell lines, significant divergence became apparent after approximately 48 h, whereas MaMel-02 cells exhibited earlier responses. *: p < 0.05; **: p < 0.01; ***: p < 0.001; ****: p < 0.0001.

We next examined the impact of the selected microRNAs on tumor cell proliferation. Cell growth and viability were monitored through live-cell imaging (Fig. 5). Tumor cell lines of three entities, namely breast cancer (MDA-MB-231), melanoma (MaMel-02), and colon cancer (HT-29) were analyzed. Over a period of three days, transfection with miR-155-5p, miR-3535, or miR-9-5p led to a significant reduction in tumor cell proliferation, reflected by decreased confluency compared to treatment with the control miRNA. In MaMel-02 cells, growth inhibition started particularly early, becoming detectable after approximately 10 h (miR-155-5p), 18 h (miR-9-5p) and 36 h (miR-3535). In contrast, the growth curves of MDA-MB-231 and HT-29 cells started to diverge from control curves at later time points, i.e. 44–48 h after transfection of miR-155-5p, miR-3535 or miR-9-5p in MDA-MB-231 cells and 38–50 h after transfection of these miRNAs in HT-29 cells. Among the cell lines tested, miR-155-5p consistently exerted the strongest anti-proliferative effect, while the effect of miR-9-5p was less pronounced in HT-29 cells.

### Pan-functional miRNAs exert convergent immunoregulatory effects through distinct mechanisms in tumor cells and macrophages

Having established coordinated effects of miR-155-5p and miR-3535 across tumor cells and macrophages, we next investigated their potential mode of action. Regarding the impact of miR-155-5p and miR-3535 on NT5E and CD274 surface expression in tumor cell lines, in silico analysis revealed predicted binding sites within the 3′UTRs of the respective mRNAs (Additional file 1: Fig. S7), suggesting potential canonical miRNA-mediated regulation of translation. However, given the functional complexity of miRNA activity and the absence of definitive reporter-based target validation set up in the present study, we interpret the observed downregulation of NT5E and CD274 as functional regulation that may involve direct as well as indirect mechanisms. Notably, both miRNAs reduced ICM expression at the cell-surface as assessed by flow cytometry, supporting biologically relevant regulation of these immunomodulatory molecules.

**Fig. 6:**
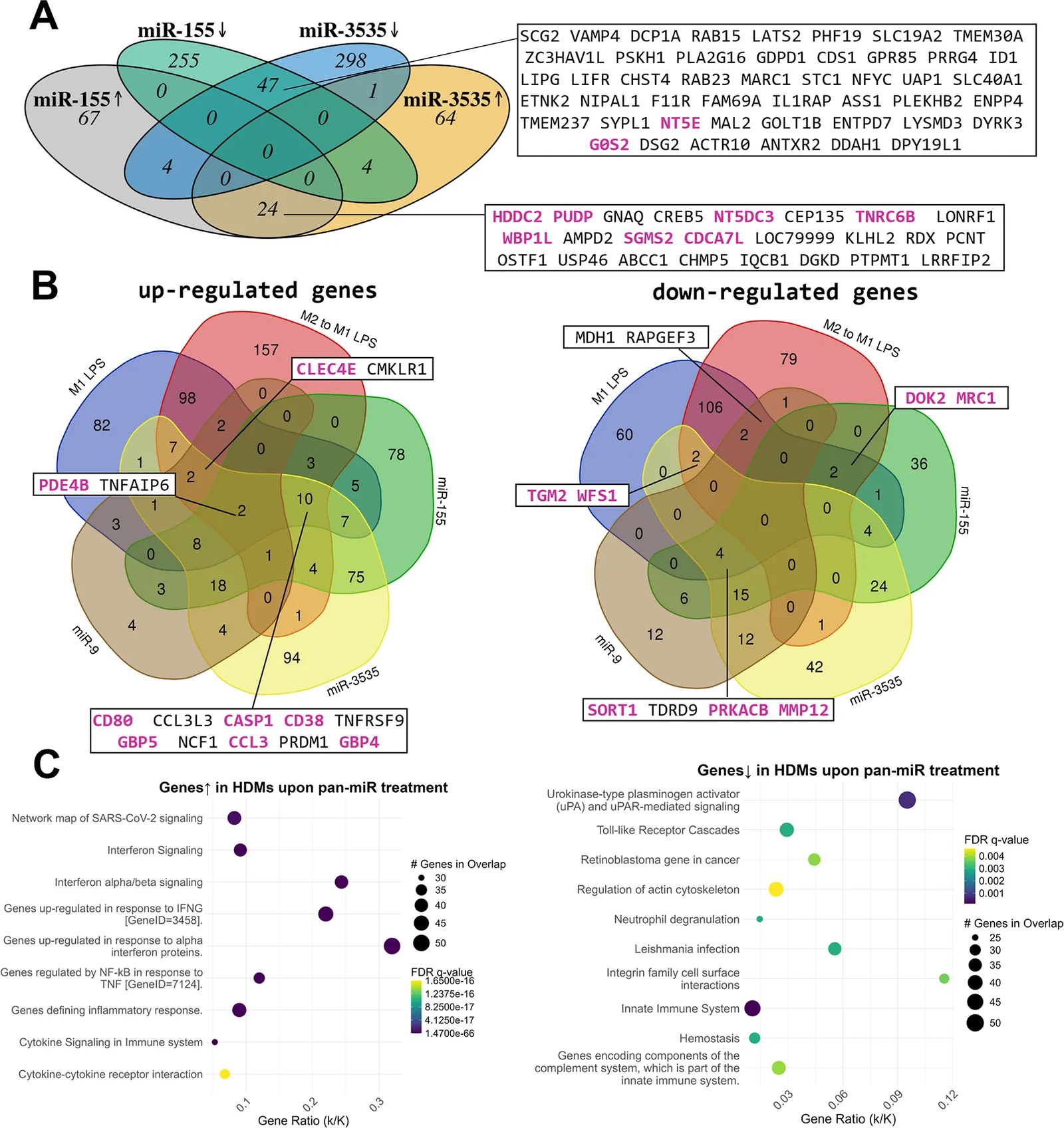
Pan-functional miRNAs induce convergent transcriptional programs through distinct regulatory mechanisms in tumor cells and macrophages. **A**: Venn diagram showing genes whose expression is jointly modulated by pan-functional miRNA. Gene expression microarray analysis of the MDA-MB-231 breast cancer cell line was performed 48 h post miRNA transfection. Differential gene expression was determined relative to control cells transfected with ath-miR-416. Three biological replicates were performed per condition. Genes were defined as differentially expressed (DEGs) using a threshold of p < 0.05, with fold change (FC) > 1.5 for upregulated genes and FC < 0.75 for downregulated genes. Genes classified as strongly regulated (FC < 0.5 for downregulation; FC > 2.0 for upregulation) are highlighted in pink. **B**: Microarray data of miRNA transfected and LPS/IFNγ repolarized HMDMs. Genes marked in pink are implicated in the regulation or characterization of M1/M2 polarization. **C**: Enriched pathways in M2-polarized HMDMs upon pan-functional miRNA treatment.

To further investigate the coordinated impact of these miRNAs in tumor cells, gene expression profiling was performed in miRNA-transfected MDA-MB-231 cells, and the complete processed gene expression dataset is provided in Additional file 6. Microarray analysis (Fig. 6A) identified 401 differentially expressed genes (DEGs) following miR-155-5p transfection and 442 DEGs after miR-3535 transfection. Among these DEGs, 117 genes were strongly downregulated by miR-155-5p (fold change < 0.5) and expression of 84 genes was reduced by miR-3535. Conversely the expression of 32 genes was strongly upregulated by miR-155-5p (fold change > 2.0) and 21 genes were upregulated by miR-3535 (Additional file 1: Fig. S8). Considering DEGs with fold change > 1.5, both miRNAs jointly increased expression of 24 genes, including CREB5, GNAQ, LONRF1, and USP46 (Fig. 6A). Among genes with a fold change < 0.75, both miRNAs reduced expression of 47 genes, including NT5E, G0S2, and RAB15. Notably, miR-155-5p significantly reduced mRNA levels of both immune checkpoint molecules NT5E and CD274 (Fig. 6A). Among the genes downregulated by miR-155-5p, 174 of 306 genes were identified as known or predicted direct targets, consistent with canonical microRNA-mediated repression via 3-UTR binding. In contrast, only 12 of 95 upregulated genes contained predicted miR-155-5p binding sites, indicating a significant enrichment of direct targets among the downregulated gene set (Fisher’s exact test, *p* < 0.0001; Additional file 1: Fig. S8). Gene set enrichment analysis of genes jointly downregulated by miR-155-5p and miR-3535 revealed enrichment of cellular component categories related to membranes and vesicular structures (Additional file 1: Table S4). Conversely, genes jointly upregulated by both miRNAs showed enrichment of the sphingolipid signaling pathway (*p* = 0.0117; genes: GNAQ, SGMS2, ABCC1) and predicted targets of hsa-miR-375 (*p* = 0.022; genes: PUDP, SGMS2, CDCA7L, USP46, DGKD, PTPMT1).

### Transcriptional reprogramming of M2-like macrophages by pan-functional miRNAs reflects an M1-like phenotype

To further characterize the global effects of the identified miRNAs, gene expression profiling was performed in human M2-like macrophages following transfection with miR-155-5p, miR-3535, miR-9-5p or control miRNA. As reference, cytokine-polarized M1 macrophages (IFNγ or IFNγ + LPS), IL4–polarized M2-like macrophages, and macrophages subjected to M2-to-M1 repolarization (initial IFNγ + LPS treatment followed by IL4 exposure) were included.

We found that 29 genes, including APOBEC3A and PDE4B, were consistently upregulated by all three miRNAs in transfected M2 -like macrophages (Fig. 6B, Additional file 1: Fig. S9). In addition, miR-155-5p and miR-3535 shared 125 upregulated genes, including CD80, TAP1, CD38, and TNF (Additional file 1: Fig. S9). Notably, 39 of 48 genes upregulated by miR-9-5p were also induced by at least one of the other miRNAs, indicating a broadly conserved pro-inflammatory transcriptional program across the identified pan-functional miRNAs. To assess whether the observed transcriptional changes reflected bona fide M1 polarization programs, we compared miRNA-induced expression profiles with those of IFNγ/LPS-polarized macrophages and macrophages subjected to M2-to-M1 repolarization. Two genes, PDE4B and TNFAIP6, were consistently upregulated across all pan-functional miRNAs and were similarly induced during both LPS-mediated M1 differentiation and LPS-driven repolarization of M2-like macrophages (Fig. 6B, left panel). Moreover, increased expression of classical M1-associated markers, including CD80, CD38, CCL3, CASP1, and GBP5, was shared between macrophages transfected with miR-155-5p or miR-3535 and cytokine-polarized M1-like macrophages. Upregulation of CLEC4E and CMKLR1 was observed across all three miRNA-treated groups and in both LPS- and LPS/IFNγ-polarized M1 conditions, further supporting their role as common markers of pro-inflammatory macrophage polarization. Notably, miRNA-mediated gene repression in macrophages was overall less prominent than gene induction following transfection with miR-3535, miR-155-5p, or miR-9-5p. Only 19 genes, including CD180, MMP12, and SORT1, were jointly downregulated by all three miRNAs, whereas additional 47 genes were jointly suppressed by miR-155-5p and miR-3535 (Additional file 1: Fig. S10). Notably, no genes were consistently downregulated across all five groups, comprising miRNA-transfected and externally polarized macrophages (Fig. 6B, right panel).

Overlap with LPS- or LPS/IFNγ-induced M1 macrophages was limited; however, selected genes showed partial convergence. For example, MRC1 and DOK2 were downregulated in miR-155-5p–treated macrophages and in both M1 reference groups, while RAPGEF3 and MDH1 were reduced in miR-3535–treated macrophages as well as in LPS-polarized and M2-to-M1–repolarized macrophages. Among the 19 genes jointly suppressed, only NNT and TGFBR2 contained predicted binding sites for all three miRNAs, suggesting that indirect post-transcriptional regulatory mechanisms contribute to the observed transcriptional changes.

### Pan-functional miRNAs promote M1-like macrophage polarization through induction of transcriptional regulatory programs

Next, we investigated potential regulatory drivers underlying the changes in gene expression observed in miRNA-transfected macrophages. Gene Set Enrichment Analysis (GSEA) was performed using the set of genes upregulated by at least two of the three pan-functional miRNAs. This analysis revealed significant enrichment of pathways associated with pro-inflammatory and innate immune responses, including type I and type II interferon signaling, TNFα/NF-κB signaling, antigen processing and presentation, Toll-like receptor signaling, and cytokine–cytokine receptor interactions (Table 2; Fig. 6C). These pathways are characteristic of M1-like macrophage activation.

Moreover, upstream regulator analysis using GSEA of transcription factor target (TFT) gene sets, focusing on genes significantly upregulated in macrophages by at least two of the three pan-functional miRNAs, identified the TFs STAT1/2, IRF1, IRF2, IRF7, IRF8, NFKB1, and ZNF597 as significantly enriched drivers of the pan-miRNA-induced gene expression. Importantly, based on our RNA-seq data of cytokine-polarized macrophages, seven of these TFs were significantly upregulated in M1-like compared to M2-like macrophages (see Additional file 1: Fig. S11), indicating their functional relevance for M1 polarization. Moreover, pan-functional miRNA transfection itself induced expression of several of these regulators: miR-3535 significantly upregulated STAT1, IRF1, and IRF7 expression, while miR-155-5p also increased STAT1 expression and showed a trend toward upregulation of IRF1 and IRF7 expression (see Additional file 1: Fig. S12).

To further dissect this regulatory landscape, we applied a TF activity model [47] to infer activity scores of 1160 TFs based on microarray data. We identified a subset of TFs showing consistently elevated activity in all three pan-functional miRNA-treated groups, including IRF7 and IRF9—factors with implicated roles in interferon responses and pro-inflammatory signaling. Conversely, TFs such as HOXA5, JUND/FRA1, E2F3/DP1, and ZNF703 showed reduced activity upon miRNA transfection. Notably, ZNF703, a known transcriptional co-repressor, is predicted as direct target of both miR-155-5p and miR-9-5p and was found highly expressed in M2-like macrophages (see Additional file 1: Fig. S13). Thus, inhibited ZNF703 expression may contribute to de-repression of M1 genes.

**Table 2:** Enriched Gene sets and pathways in human anti-inflammatory macrophages (M2-like) upon treatment with pan-functional miRNAs. Results are shown for genes whose expression was modulated by at least two pan-functional miRNAs. k/K: Ratio of k number of overlapping genes and K total genes in the pathway; FDR: False-Discovery-Rate

Genes up-regulated upon pan-functional miRNA treatment			
Description	k/K	*p*-value	FDR q-value
Genes up-regulated in response to IFNG [GeneID = 3458].	0.2200	3.7E-70	1.47E-66
Genes up-regulated in response to alpha interferon proteins.	0.3196	6.93E-55	1.37E-51
Cytokine Signaling in Immune system	0.0532	6.77E-40	8.95E-37
Genes regulated by NF-kB in response to TNF [GeneID = 7124].	0.1200	2.82E-31	2.56E-28
Interferon alpha/beta signaling	0.2436	3.23E-31	2.56E-28
Interferon Signaling	0.0916	1.56E-29	1.03E-26
Genes defining inflammatory response.	0.0900	2.44E-21	1.39E-18
Network map of SARS-CoV-2 signaling	0.0826	1.18E-20	5.84E-18
Cytokine-cytokine receptor interaction	0.0682	3.74E-19	1.65E-16
**Genes down-regulated upon pan-functional miRNA treatment**			
**Description**	**k/K**	**p-value**	**FDR q-value**
Innate Immune System	0.0106	4.01E-8	1.59E-4
Urokinase-type plasminogen activator (uPA) and uPAR-mediated signaling	0.0952	3.58E-7	7.1E-4
Leishmania infection	0.0556	3.19E-6	2.93E-3
Toll-like Receptor Cascades	0.0294	4.02E-6	2.93E-3
Hemostasis	0.0118	4.35E-6	2.93E-3
Neutrophil degranulation	0.0146	4.43E-6	2.93E-3
Integrin family cell surface interactions	0.1154	6.42E-6	3.64E-3
Retinoblastoma gene in cancer	0.0444	7.77E-6	3.85E-3
Genes encoding components of the complement system, which is part of the innate immune system.	0.0250	8.86E-6	3.91E-3
Regulation of actin cytoskeleton	0.0235	1.2E-5	4.6E-3

Finally, we compared DEGs in MDA-MB-231 breast cancer cells and M2-like polarized human macrophages transfected with either miR-155-5p or miR-3535 to assess shared transcriptional programs induced by pan-functional miRNAs across cell types (see Additional file 1: Fig. S14). Regarding miR-155-5p, 631 DEGs were identified and only 1% of these genes were uniformly altered in both tumor cells and macrophages. Among these, five genes were upregulated in both cell types, including TSC22D2, RHOB, STX11, DUSP1, and TNFAIP3, while three genes (SORT1, HSD17B12, and MYO1D) were consistently downregulated. Notably, SGMS2 and CDCA7L were regulated in opposite directions, suggesting cell-type-specific responses (Additional file 1: Fig. S14, left).

With respect to miR-3535, 643 DEGs were detected. Six genes were jointly upregulated, including ARL5B, USP42, NEXN, ADM, STX11, and SAV1, while five genes (NCAPH, PLBD2, MAN1A1, SC5D, and CBX5) were downregulated in M2 polarized macrophages and in MDA-MB-231 cells (Additional file 1: Fig. S14, right). Similarly to miR-155-5p, miR-3535 transfection also resulted in opposite regulation, observed for CDCA7L and additionally for GCH1. Notably, STX11 emerged as the only gene consistently upregulated by both miR-155-5p and miR-3535 in tumor cells and macrophages. STX11 encodes a vesicle-associated membrane trafficking regulator implicated in immune effector functions [48–50]. Analysis of TCGA breast cancer datasets revealed that high STX11 expression was significantly associated with improved overall survival (Additional file 1: Fig. S15), supporting its potential clinical relevance. Consistent with this observation, miR-155 expression showed a strong positive correlation with STX11 mRNA levels across TCGA breast cancer and melanoma cohorts (see Additional file 1: Fig. S15), further linking this gene to the pan-functional microRNA-associated regulatory program.

Correlation analysis of TCGA breast cancer transcriptomic data revealed a strong positive association between miR-155-5p expression and key genes of the interferon-driven pro-inflammatory macrophage program, including GBP5, CXCL9, CXCL10, STX11, STAT1, and IRF1 (Additional file 1: Fig. S15). These genes were also strongly correlated with each other, indicating a coordinated inflammatory transcriptional network. In contrast, ZNF703, a transcription factor associated with pro-tumoral and immunosuppressive macrophage states, showed a pronounced negative correlation with miR-155-5p and with the above inflammatory genes, with the strongest inverse association observed between miR-155-5p and ZNF703.

Together, these findings support the presence of a conserved interferon-associated regulatory axis linked to miR-155-5p expression and suggest that suppression of ZNF703 may represent a complementary mechanism contributing to the pro-inflammatory transcriptional reprogramming observed in our experimental models. In this context, the data further highlights the context-dependent, yet partially convergent effects of pan-functional miRNAs and identify a limited core of shared regulatory targets across immune cell and tumor cell compartments.

## Discussion

The role of miR-155 has long been debated, as it has been described both as an oncomiR [51] and as a tumor suppressor [52]. A key to resolving this apparent contradiction lies in its strong context dependence. First, the effect of miR-155 varies with cell type: in tumor cells it can promote proliferation and survival pathways [53, 54], whereas in immune cells it frequently enhances activation and effector functions. Second, the outcome may depend on the tumor entity, with reports of oncogenic roles in lymphomas [55, 56] contrasting with tumor-suppressive functions in certain solid cancers [57, 58]. Third, the tumor microenvironment adds an additional layer of regulation: depending on the cytokine milieu and stromal interactions, miR-155 can either reinforce immunosuppression or promote anti-tumor immunity [58, 59]. In line with the reported oncogenic functions, elevated expression of miR-155 has been associated with poor prognosis in hematological malignancies [60, 61], non-small cell lung cancer (NSCLC) [62], breast cancer [63, 64], and glioma [65]. However, in our study, we identified miR-155-5p and miR-3535 as inhibitors of NT5E (CD73) expression in human melanoma and breast cancer cell lines, suggesting a tumor-suppressive function in this context. In addition, miR-155-5p also reduced CD274 (PD-L1) expression in the tumor cell lines tested, indicating potential synergistic effects on immune checkpoint downregulation brought about by this miRNA. In fact, while data on miR-155-mediated regulation of CD274 expression in tumor cells is scarce, one report demonstrated miR-155-induced PD-L1 repression in human lung adenocarcinoma cells [66]. This is in line with our data showing miR-155-5p-mediated downregulation of PD-L1 in melanoma and breast cancer cells. Interestingly, upregulation of miR-155 upon TNFα/IFNγ stimulation in human lymphatic endothelial cells was also reported to result in reduced PD-L1 expression [67], suggesting a broader functional PD-L1/miR-155 axis being active in both, tumor and stromal cells. Furthermore, we observed consistent anti-proliferative effects of miR-9-5p, miR-3535, and miR-155-5p across cell lines of multiple tumor entities, supporting their putative tumor-suppressive function. Notably, in skin cutaneous melanoma (SKCM), high expression of the MIR155 host gene (MIR155HG) was significantly associated with improved overall survival (see Additional file 1: Fig. S16). This prognostic value of MIR155HG expression was also evident in patients receiving immune checkpoint blockade, where higher miR-155-5p levels correlated with superior clinical outcome [68]. Interestingly, we previously found that miR-155-5p enhances tapasin expression in cancer cells, thereby improving antigen presentation through the HLA class I pathway and increasing tumor cell recognition by immune cells [69]. In the present study, we additionally observed that both miR-3535 and miR-155-5p upregulate TAP1 and TAP2 mRNA expression in macrophages, supporting the notion that these miRNAs may propel the antigen-processing and presentation machinery, thereby promoting improved immune visibility of tumor cells.

In macrophages, miR-155 is well-established as an inflammation-associated miRNA molecule [70], since overexpression of miR-155 in tumor-associated macrophages (TAMs) has been shown to promote M1 polarization and anti-tumoral activity [71]. Similarly, in the murine PyMT breast cancer model, miR-155 was found to be upregulated in proinflammatory macrophages, and its knockdown accelerated tumor growth [72]. These findings align with our observation that transfection of miR-155-5p enhances the expression of proinflammatory cytokines and chemokines in human macrophages.

Importantly, we demonstrate that miR-3535 and miR-9-5p can likewise induce M1-like polarization in human macrophages, identifying them as novel pan-functional miRNAs capable of blunting pro-tumoral functions in both tumor cells and immune cells (i.e. macrophages). While these findings establish a clear transcriptional and cytokine signature consistent with M1 polarization, it remains to be determined whether this reprogramming also translates into *bona fide* functional properties of M1 macrophages. The induction of TNF and CXCL10 may be of particular importance for anti-tumor immunity, as TNF amplifies pro-inflammatory signaling and can sensitize tumor cells to necrosis and irradiation induced cell death [73, 74], while CXCL10 serves as a potent chemoattractant for activated T cells, thereby facilitating their infiltration into the tumor microenvironment [75, 76]. Future studies should therefore assess key effector functions such as enhanced ROS production, improved antigen processing and presentation, and the ability to activate and sustain tumor-reactive T cell responses [77, 78]. Demonstrating such functional consequences will be critical to confirm that pan-functional miRNAs not only shift marker expression but also endow macrophages with genuine anti-tumor activity. Importantly, the endogenous biogenesis of miR-3535 remains to be fully characterized. Since the identified sequence maps to a snoRNA-associated genomic locus, both canonical and non-canonical small RNA processing pathways are conceivable [79–81]. Nevertheless, the observed functional effects of the synthetic miR-3535 mimic demonstrate that this small RNA sequence possesses potent immunoregulatory activity independent of its current annotation status. Future studies will therefore be required to determine whether endogenous miR-3535 is generated through canonical DROSHA/DICER-dependent processing or non-canonical small RNA pathways, and whether it is physiologically incorporated into the RISC complex.

To elucidate underlying regulatory mechanisms of miRNA-mediated macrophage polarization, we applied a TF activity model and identified enhanced activity of IRF7, IRF9, and STAT1 in macrophages treated with pan-functional miRNAs. In contrast, the expression of pro-tumoral TFs such as ZNF703 was downregulated in macrophages. Notably, ZNF703 is part of a four-gene M2 macrophage-related score (M2I) linked to poor prognosis and ICB response in colorectal cancer [82], highlighting the translational relevance of M2I suppression. Upstream regulator analysis confirmed activation of key M1-associated TFs, including STAT1 and IRF1, particularly following miR-3535 and miR-155-5p transfection. Although these findings support a regulatory link between miRNA-mediated reprogramming and M1-associated transcriptional networks, causal relationships remain to be formally established and will require targeted perturbation studies. In support of the translational relevance of this regulatory axis, analysis of TCGA datasets revealed a strong positive correlation between miR-155-5p expression and STX11 mRNA levels, as well as with interferon-associated inflammatory genes including STAT1, IRF1, CXCL9, CXCL10, and GBP5. These genes formed a coordinated inflammatory module, whereas ZNF703 displayed consistent inverse correlations with miR-155-5p and the interferon-associated gene set. Moreover, elevated STX11 expression was associated with improved overall survival in breast cancer patients, further supporting its potential clinical relevance. Together, these observations extend our experimental findings and suggest the presence of a conserved interferon-linked regulatory network associated with pan-functional miRNA activity in human tumors. The TCGA data used in this study are derived from bulk tumor tissue and therefore reflect combined signals from tumor, stromal, and immune cell populations. Consequently, cell type–specific conclusions regarding macrophage polarization cannot be directly inferred from these analyses. Nevertheless, the observed association of miR-155-5p with interferon-associated inflammatory gene signatures supports the presence of a conserved pro-inflammatory network within the tumor microenvironment that is consistent with the macrophage reprogramming observed in vitro.

Importantly, our data reveal that pan-functional miRNAs exert convergent immunoregulatory effects across tumor cells and macrophages through distinct regulatory modes. In tumor cells, miRNAs predominantly mediate repression of immune-evasion pathways, including downregulation of CD274/PD-L1 as shown by Vaxevanix et al. by direct canonical interaction of miR-155-5p with the CD274/PD-L1 3′-UTR [83], whereas in macrophages they induce pro-inflammatory transcriptional programs. This dual yet mechanistically distinct mode of action underscores the complexity of miRNA-mediated regulation and may explain their capacity to coordinately influence multiple cellular components of the tumor microenvironment. Importantly, miRNA activity is not necessarily restricted to canonical seed-mediated 3′-UTR repression. In previous work, we demonstrated that miR-155-5p can enhance the HLA class I antigen-processing pathway through interaction with a transcriptional silencer element rather than through classical post-transcriptional repression [84]. Together with the present data, this supports the concept that pan-functional miRNAs may act through a combination of canonical target repression, indirect signaling effects, and non-canonical regulatory mechanisms.

Several limitations of this study should be acknowledged. In physiological settings, miRNA-mediated regulation is unlikely to be governed by single miRNAs in isolation, but rather by the combinatorial balance of multiple miRNAs acting on shared and distinct targets. While the present study focused on individual miRNA effects using mimic-based approaches, the observed convergence on pro-inflammatory transcriptional programs suggests that pan-functional miRNAs may act in a coordinated manner within endogenous regulatory networks. Future studies will therefore be required to investigate potential synergistic, additive, competitive, or antagonistic interactions between different miRNAs. Although standardized in vitro polarization models provide a controlled framework for mechanistic studies, they cannot fully recapitulate the complexity and heterogeneity of tumor-associated macrophages within human tumors. Future studies integrating single-cell transcriptomic datasets and patient-derived myeloid populations will therefore be important to further assess the physiological relevance of pan-functional miRNA-mediated macrophage reprogramming in vivo.

Another limitation of this study is that macrophage experiments were performed using cells derived from healthy donors. The observed inter-donor variability highlights the inherent heterogeneity of primary human macrophages and may reflect differences in baseline immune status, monocyte composition or differentiation efficiency. Such variability is expected in primary human cell systems and underscores the importance of validating miRNA-mediated effects across multiple independent donors. It is well established that monocytes from cancer patients can exhibit altered functional states and polarization capacities [85–87]. Future studies should therefore investigate the response of patient-derived monocytes and macrophages under clinically relevant conditions, within ethically approved clinical study frameworks. Such analyses will be essential to validate the translational applicability of pan-functional miRNA-mediated reprogramming in the context of tumor-induced immune dysfunction.

The present work is primarily based on in vitro systems, and in vivo validation will be required to determine the extent to which miRNA-mediated reprogramming alters tumor progression and immune responses in complex physiological settings, i.e. within the TME. Furthermore, although bioinformatic and transcriptional analyses suggest both direct and indirect regulatory mechanisms, comprehensive mechanistic dissection remains necessary. In particular, dual-luciferase reporter assays, mutational 3′-UTR analyses, pathway perturbation experiments, and additional protein-level validation of intracellular downstream targets will be required to distinguish canonical direct target repression from indirect network effects.

To further explore cross-species conservation of the identified pan-functional miRNAs, we additionally analyzed murine macrophage and tumor cell systems (Additional file 1: Fig. S17). RNA-sequencing of polarized murine bone marrow-derived macrophages (BMDMs) revealed conserved enrichment of miR-155-5p in M1-like macrophages, whereas miR-3535 did not show significant differential expression, suggesting partial species-specific differences in miRNA regulation. Functional experiments demonstrated that miR-155-5p induced repolarization of murine M2-like BMDMs toward a pro-inflammatory phenotype characterized by increased Cxcl10, Il1b, and Tnf expression together with reduced Mrc1 levels. In addition, miR-155-5p reduced proliferation and immune checkpoint molecule expression in the murine EO771 breast cancer cell line, supporting conserved immunomodulatory and tumor-inhibitory activity across human and murine systems. In contrast, miR-9-5p did not induce consistent repolarization effects in murine macrophages, indicating potential context- or species-dependent differences in miRNA function.

To further assess functional consequences of macrophage repolarization, we additionally performed macrophage–tumor and macrophage–T cell co-culture experiments (Additional file 1: Fig. S18). Conditioned medium from classically polarized M1-like macrophages significantly reduced EO771 tumor cell proliferation and enhanced IFNγ secretion in EO771-OVA/OVA-CTL co-culture systems, confirming functional anti-tumoral and T-cell stimulatory properties of pro-inflammatory macrophages. Importantly, conditioned medium from miR-155-5p-transfected macrophages also enhanced IFNγ ELISpot formation, although the functional effects were less pronounced than those induced by classical IFNγ + LPS-mediated M1 polarization. Furthermore, direct transfection of EO771-OVA tumor cells with miR-3535 significantly increased CTL activation, suggesting additional tumor cell–intrinsic immunomodulatory mechanisms beyond macrophage reprogramming. Together, these findings indicate that miRNA-mediated reprogramming can induce biologically relevant functional changes, while likely representing partial or intermediate activation states rather than fully cytokine-driven M1 phenotypes.

While these findings do not replace in vivo validation, they provide an experimental basis for future syngeneic tumor studies investigating macrophage polarization, immune-cell infiltration, and tumor growth following miRNA delivery in vivo.

Future studies using advanced tumor model systems, including 3D tumor–immune co-cultures or in vivo delivery platforms such as lipid nanoparticles, will be essential to evaluate therapeutic potential. In addition, more comprehensive functional analyses, including extended macrophage–T cell interaction assays, tumor cell killing assays, phagocytosis assays, and longitudinal in vivo immune profiling, will be required to further dissect the immunological consequences of miRNA-mediated tumor microenvironment reprogramming. Previous clinical experience with miRNA-based therapeutics [88] highlights both promise and challenges, emphasizing the importance of optimized delivery and safety profiling.

## Conclusions

Our data identify miR-155-5p, miR-3535, and miR-9-5p as pan-functional miRNAs that can simultaneously target tumor-intrinsic immune checkpoints and reprogram macrophages toward an M1-like, pro-inflammatory phenotype. By repressing the expression of immunosuppressive molecules such as CD274 and NT5E in tumor cells and activating M1-associated transcriptional programs in macrophages, these miRNAs orchestrate a multi-layered anti-tumoral immune response. Particularly, the regulatory impact on clinically relevant factors such as ZNF703 and STX11 further underscores their translational potential in counteracting M2-driven immunosuppression. Clinical relevance of miR-155-5p is supported by TCGA and immunotherapy-treated cohorts, where elevated MIR155HG expression correlates with improved patient survival and inflammatory anti-tumor signatures. In contrast, miR-3535 represents a newly identified regulatory small RNA whose endogenous expression and clinical significance remain to be established. Future translational studies in patient-derived samples will therefore be required to validate the clinical relevance of pan-functional miRNAs in tumor immunity and therapeutic response. Additional analyses in murine macrophage and tumor cell systems further support partial cross-species conservation of miR-155-5p-mediated immunoregulatory effects and provide an experimental basis for future syngeneic in vivo studies. Our findings therefore highlight the potential ability of individual miRNAs to function as multi-level immunomodulators across different cell types within the TME. Importantly, the observed effects are unlikely to rely solely on canonical post-transcriptional repression of direct targets but rather involve cell type–specific signaling cascades, indirect feedback loops, and possibly non-canonical functions. Future mechanistic studies, including direct target validation and pathway perturbation experiments, will be required to disentangle canonical versus non-canonical, direct versus indirect regulatory effects and to assess potential off-target consequences. Importantly, comprehensive validation in appropriate in vivo tumor models will be essential to determine the extent to which pan-functional miRNAs can reprogram the tumor microenvironment, modulate immune infiltration, and ultimately control tumor growth in a physiological context. Such efforts will be critical to translate pan-functional miRNA strategies into clinically viable approaches for conditioning the tumor microenvironment and enhancing immunotherapeutic efficacy.

## Supplementary information

Below is the link to the electronic supplementary material.


Supplementary Material 1 : Additional File 1: Supplementary Materials and Methods, Supplementary Tables S1–S4, and Supplementary Figures S1–S18.



Supplementary Material 2 : Additional File 2: Processed RNA sequencing dataset of HMDMs. 



Supplementary Material 3 : Additional File 3: Processed miRNA sequencing dataset of HMDMs.



Supplementary Material 4 : Additional File 4: Processed microarray gene expression dataset of pan-miRNA treated HMDMs.



Supplementary Material 5 : Additional File 5: Predicted binding sites of M1-enriched miRNAs in M2-upregulated genes.



Supplementary Material 6 : Additional File 6: Processed microarray gene expression dataset of MDA-MB-231 cells.


## Data Availability

Original data are stored at the senior author’s lab and can be provided upon request. The datasets supporting the conclusions of this article are included within the article and its additional files.

## Abbreviations

ADCY1: Adenylate cyclase 1
APOBEC3A: Apolipoprotein B mRNA editing enzyme catalytic subunit 3 A
CAR: Chimeric antigen receptor
CCL: C-C motif chemokine ligand
CD: Cluster of differentiation
CXCL: C-X-C motif chemokine ligand
DEG: Differentially expressed gene
ELISA: Enzyme-linked immunosorbent assay
ENTPD1 (CD39): Ectonucleoside triphosphate diphosphohydrolase 1
FBS: Fetal bovine serum
FC: Fold change
GSEA: Gene set enrichment analysis
HMDM: Human monocyte-derived macrophage
ICB: Immune checkpoint blockade
ICM: Immune checkpoint molecule
IFNγ: Interferon gamma
IL: Interleukin
IRF: Interferon regulatory factor
LNP: Lipid nanoparticle
LPS: Lipopolysaccharide
M0: Non-polarized macrophage
M1: Pro-inflammatory macrophage phenotype
M2: Anti-inflammatory macrophage phenotype
M2I: M2 macrophage-related score
miRNA: MicroRNA
NT5E (CD73): Ecto-5′-nucleotidase
PBMC: Peripheral blood mononuclear cell
PD-L1 (CD274): Programmed death ligand 1
PCC: Pearson correlation coefficient
qPCR: Quantitative polymerase chain reaction
RNA-seq: RNA sequencing
STAT: Signal transducer and activator of transcription
STX11: Syntaxin 11
TAM: Tumor-associated macrophage
TAP1: Transporter associated with antigen processing 1
TAP2: Transporter associated with antigen processing 2
TF: Transcription factor
TGF-β: Transforming growth factor beta
TME: Tumor microenvironment
TNFα: Tumor necrosis factor alpha
UTR: Untranslated region
ZNF703: Zinc finger protein 703
