# Supplementary material for: miR-155-5p drives coordinated tumor and macrophage reprogramming across multiple cancer entities
Source: BMC Med. 2026 Aug 27;24:466. doi: 10.1186/s12916-026-05146-7 (PMC13523270; doi:10.1186/s12916-026-05146-7)
Supplement: Supplementary file 1 [file 12916_2026_5146_MOESM1_ESM.pdf]

Supplemental Material to the paper:

miR-155-5p drives coordinated tumor and macrophage reprogramming across multiple cancer entities

Theresa Kordaß, Ann-Katrin Schlosser, Marijan Czygan, Luisa Vieira Codeco Marques, Melisa Wartusch, Eric Nerenz, Vincencia Stefanie Muliawan, Stephan Kersting, Wolfram Osen, Stefan B. Eichmüller

Supplementary Materials and Methods

In silico miRNA prediction

Ten publicly available data bases were used to determine miRNAs predicted to regulate expression of CD274, ENTPD1 and NT5E or genes related to macrophage polarization state. The following resources were downloaded at 01-05-2017 and used for *in silico* predictions:

- MicroCosm
- miRanda: microRNA conserved and non-conserved
- miRDB V5
- miRecords
- miRGator
- miRNA Map
- PACCMIT
- PicTar
- PITA Top Score and all predictions
- TargetScan

miRNA transfection cancer cells

For validation of the miRNA library screen individual transfections were performed. Therefore, cells were seeded in 12 well plate and grown to 70 % confluency on day of transfection e.g., 2 x 10^5 cells were seeded and cultured 24 h until transfection. Cells were transfected with Lipofectamine RNAiMax transfection reagent according to the manufacturer's protocol. Cells were transfected with 50 nM miRNA or siRNA. A non-targeting control microRNA (ath-miR-416), lacking sequence homology to human transcripts, was included in all experiments to control for non-specific effects of small RNA delivery and transfection reagent exposure. For RNA isolation and subsequent qPCR or Microarray analysis cells were harvested 48 h post transfection. For measuring changes in cell surface expression by flow cytometry cells were harvested 72 h post transfection. For selected experiments, miRNA mimics were tested at concentrations of 10 nM, 25 nM, and 50 nM to assess dose-dependent effects on target gene expression.

RNA extraction and qPCR

Total RNA from frozen cell pellets was isolated using miRNeasy Mini Kit according to the manufacturer's protocol, which enables analysis of miRNA expression as well as mRNA expression. Cell pellets were lysed using with QIAzol lysis reagent which is based on phenol/guanidine. Subsequently, RNA was purified with silica-membrane spin columns and eluted in 30 µL nuclease-free water. For sole analysis of mRNA expression, total RNA was isolated using the RNAeasy Kit according to the manufacturer's protocol. Until further usage for cDNA synthesis, RNA samples were stored at -80 °C. RNA samples were measured using a NanoDrop spectrophotometer or Qubit device using RNA BR Assay kit. For mRNA, 500 ng of total RNA were reverse transcribed in a 20 µL reaction utilizing oligo(dT)18 primer using the Transcriptor First Strand cDNA Synthesis Kit (Roche Applied Science, Mannheim, Germany). For miRNA, 40 ng of total RNA were reverse transcribed in a 15 µL reaction using TaqMan™ MicroRNA reverse transcription kit (Applied Biosystems, Foster City, CA, USA) according to the manufacturer's protocol and specific stem-loop primers for mature miRNA. All PCRs were run on a Veriti 96 well Thermal Cycler or QuantStudio 3 Real-Time PCR System (ThermoFisher Scientific, Waltham, USA). To perform qPCR with cDNA synthesized from mRNA, 2 µL cDNA diluted 1:5 with PCR-quality water was used in a 20 µL reaction using the SYBR Green PowerUP PCR Mastermix for qPCR. Suitable housekeeping (HK) genes were used to normalize samples. The HKs were selected based on absence of putative binding site for transfected miRNAs. In general, two HK genes were used per experiment, mainly RPL19 and TBP. For cDNA synthesized from miRNA, 2 µL were used in a 20 µL PCR reaction and small nuclear RNA U6 (RNU6B) was used as endogenous control. For all samples three technical replicates were performed, and relative expression was calculated according to the $2^{-\Delta\Delta Ct}$-method.

Flow cytometry

Flow cytometry was performed using a BD FACSCanto™ II (DKFZ Core Facility). Cells were harvested, washed twice with PBS, and resuspended in FACS buffer (PBS + 2% FCS). Surface staining was performed with fluorochrome-conjugated antibodies (see Supplementary Table S1) for 30 minutes at 4 °C in the dark. After a final wash, cells were analyzed immediately.

The gating strategy included the exclusion of doublets (FSC-A vs. FSC-H) and dead cells (via viability dye exclusion). Gates for marker expression were defined using corresponding isotype controls. An example gating strategy is shown below. Data acquisition and analysis were carried out using FlowJo software (v7).

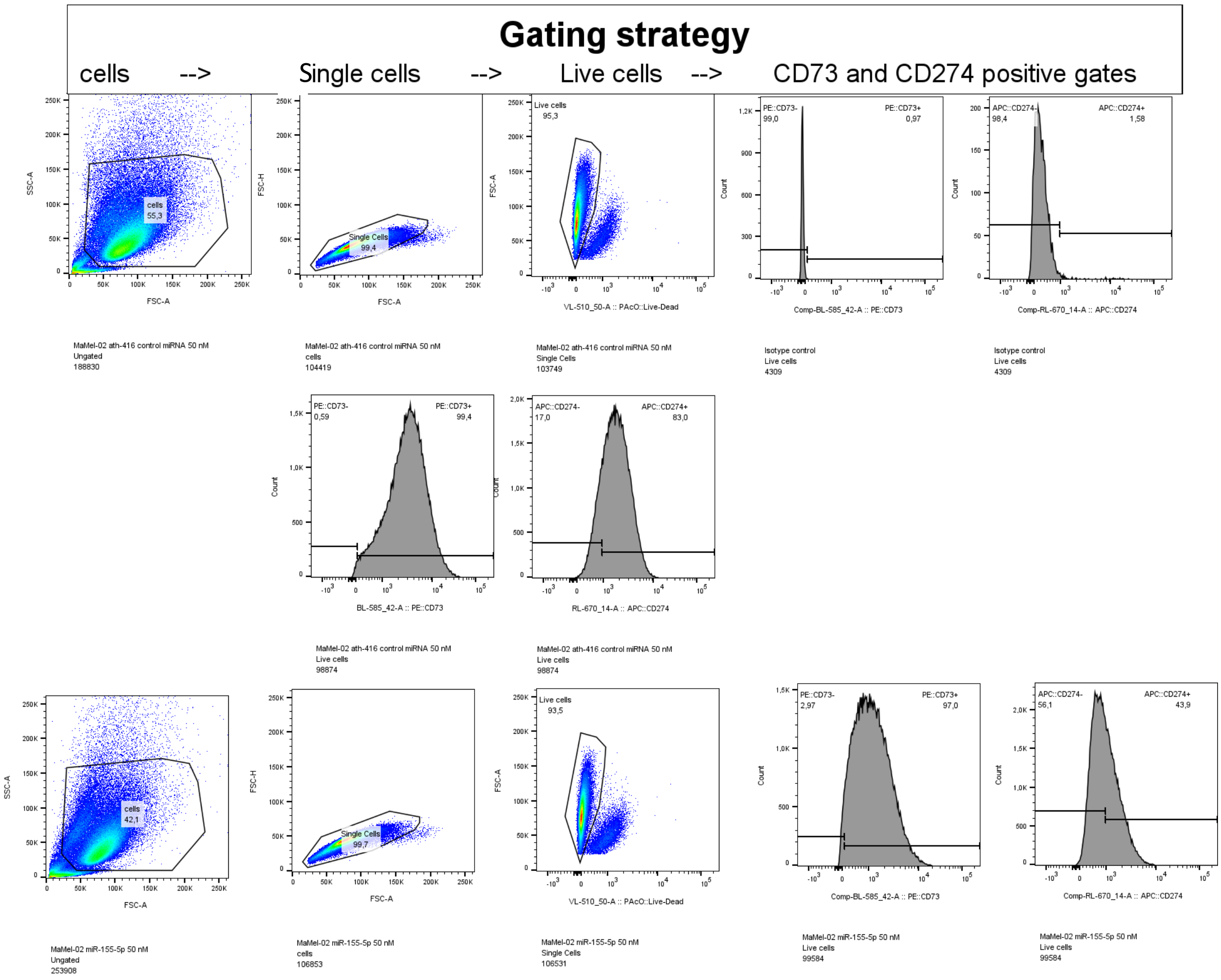

Flow cytometry analysis of macrophage polarization

For flow cytometric analysis of macrophage polarization, harvested cells were stained with Live/Dead viability dye and fluorochrome-conjugated antibodies against CD11b, CD80, CD38, CD206, and CD209. Cells were first gated according to forward and side scatter to exclude debris, followed by doublet exclusion. Dead cells were excluded using Live/Dead staining. Subsequent analyses were performed on viable CD11b^{+} monocyte/macrophage-derived cells. M1-like polarization was assessed based on CD80 and CD38 expression, whereas M2-like polarization was evaluated using CD206 and CD209 expression.

The following panel was used: CD80-AF488 (BL530/30), CD209-PE (584/42), CD11b-PerCP-Cy5.5 (BL670LP), CD206-APC (RL670/14), CD38-BV421 (VL450/50), and Live/Dead using LIVE/DEAD™ Fixable Yellow Dead Cell Stain Kit / Pacific Orange (VL510/50).

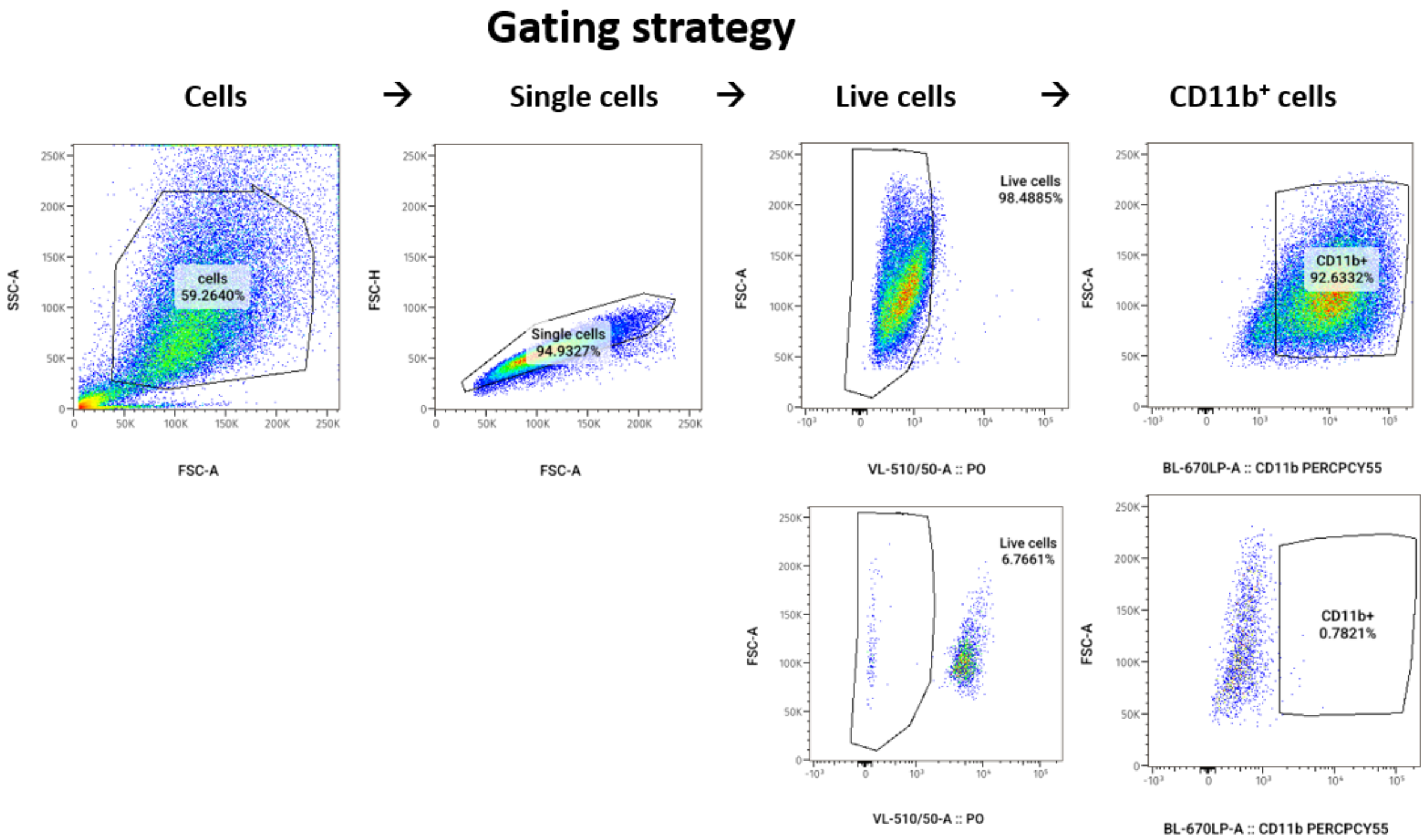

Enzyme-Linked Immunosorbent Assay ELISA

The supernatants from polarized and treated HMDMs were collected, and the concentration of secreted cytokines was quantified using ELISA MAX™ Deluxe kit for TNFα and CXCL10, following the manufacturer's instructions. The ELISA plates were read using the CLARIOstar Plus Plate reader. The process included subtracting the average absorbance of the blank replicates from the average absorbance of the sample replicates. Cytokine concentrations were ascertained by comparing the sample absorbance values to a standard reference curve, following the manufacturer's instructions.

Bioinformatic analyses

Functional Enrichment Analysis of Differentially Expressed Genes Using g:Profiler

To elucidate the biological significance of the DEGs identified in my RNAseq and Microarray data, we employed g:Profiler (Kolberg et al., 2023). g:Profiler mapped DEGs to Gene Ontology (GO) terms, and we specifically focused on Biological Processes (BP) pathways. This analysis allows for the

identification of over-represented GO:BP terms, providing insights into the biological mechanisms and processes most impacted by our experimental conditions. The analysis began with the preparation of a curated list of DEGs, which was then inputted into g:Profiler. We ensured the use of the most recent gene annotations to enhance the accuracy of our analysis. The g:Profiler tool was configured to perform an enrichment analysis, focusing on GO:BP terms to discern the biological pathways that were predominantly influenced by the observed gene expression changes.

TCGA data analysis

Publicly available datasets from The Cancer Genome Atlas (TCGA) were used to evaluate clinical and transcriptional associations of pan-functional miRNA target genes. For breast cancer (TCGA-BRCA) and melanoma (TCGA-SKCM), matched miRNA expression, mRNA expression, and clinical data were downloaded from the Broad Institute Firehose repository. Expression values provided as log2-transformed RPKM were used for downstream analyses. For correlation analyses, samples with available miRNA and mRNA expression data were included. Associations between miR-155-5p expression and selected genes of interest (including STX11, STAT1, IRF1, CXCL9, CXCL10, GBP5, and ZNF703) were assessed using Pearson correlation coefficients. Correlation strength was interpreted based on Pearson correlation coefficient (PCC), and statistical significance was determined using two-sided tests. For survival analyses, patients were stratified into high and low expression groups based on median gene expression. Overall survival was analyzed using Kaplan–Meier estimates and compared using the log-rank test. Statistical analyses were performed in R (version 4.4.3) using standard packages for survival and correlation analysis.

Transfection of Small RNAs into macrophages

Initially, we evaluated various transfection reagents, to identify the most efficient one for delivering miRNAs into macrophages. For this purpose, we utilized 20 nM of a Cy3 Dye-Labelled PremiR Negative Control, adhering to the manufacturer’s instructions for each transfection reagent. Our evaluation criteria included the efficiency of Cy3 incorporation, cell viability assessed 24 hours post-transfection, and the impact of these reagents on the macrophage phenotype, as determined by FACS analysis of Cy3, Live/Dead fixable yellow. The results led us to select DharmaFect 4 as our transfection reagent of choice for macrophages, based on its superior performance in terms of high Cy3 uptake, minimal impact on cell viability, and negligible influence on cell polarization. Following this, DharmaFect 4 was employed to transfect the miRNAs or siRNAs listed into both IL4-treated HMDMs, in accordance with the manufacturer’s guidelines. To ensure sustained intracellular expression levels of small RNA, cells were retransfected at 24-hour intervals after the initial transfection. A non-targeting control microRNA (ath-miR-416), lacking sequence homology to human transcripts, was used as negative control in all transfection experiments to account for non-specific effects of small RNA delivery and transfection reagent exposure. For silencing M2-regulators we used ON-TARGETplus siRNA Smartpools (Dharmacon) consisting of four different siRNA sequences. Cell morphology, adherence, and overall cell density of transfected HMDMs were routinely monitored microscopically throughout the experiments to assess potential cytotoxic effects associated with harvesting and DharmaFECT-mediated miRNA transfection. Representative microscopy images of transfected HMDMs are shown below.

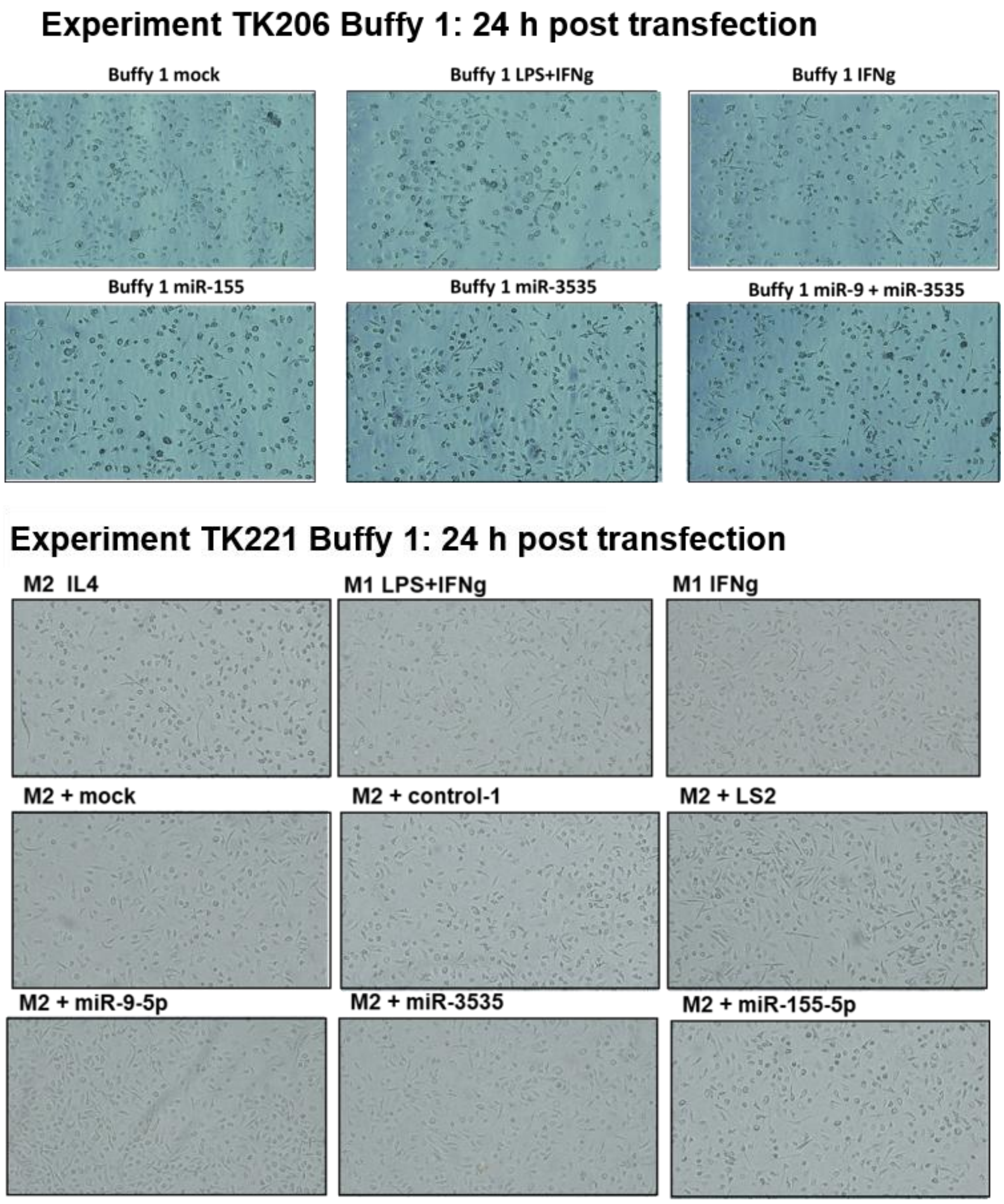

Treatment of M2-polarized macrophages with small molecule inhibitors

M2-like polarized human macrophages were generated by treating monocyte-derived macrophages with IL-4 (20 ng/mL) for 24 hours. After polarization, cells were treated with selected small molecule inhibitors to assess their effect on macrophage phenotype and gene expression. All compounds were added directly to the culture medium at the indicated concentrations (see Supplementary Table S4) and incubated for 48 hours unless otherwise specified. Applied inhibitor concentrations were selected based on published IC50 values, previously reported functional studies, and dose-finding pilot experiments, with the aim of achieving pathway modulation while minimizing cytotoxic effects. Under the applied treatment conditions, no overt cytotoxicity, including major morphological alterations, loss of adherence, or substantial reductions in cell density, was observed.

Supplementary Tables

Table S1. Antibodies used for flow cytometry.

Antibody	Type	Catalogue #	Manufacturer
**PE anti-human CD73**	IgG1, κ	344003	Biolegend, San Diego, USA
**BB515 anti-human CD39**	IgG2b, κ	565469	Becton Dickinson, Franklin Lakes, USA
**PE-Cy7 anti-human CD274**	IgG1, κ	25-5983-41	Invitrogen, Carlsbad, USA
**PE isotype control**	IgG1, κ	12-4714-81	ebioscience, San Diego, USA
**PE-Cy7 isotype control**	IgG1, κ	400125	ebioscience, San Diego, USA
**BB515 isotype control**	IgG2b, κ	564510	Becton Dickinson, Franklin Lakes, USA
**AF488 anti-human CD80**	IgG1, κ	305213	Biolegend, San Diego, USA
**APC anti-human CD206**	IgG1, κ	321109	Biolegend, San Diego, USA

Table S2. List of miRNAs used in this study.

miRNA	Sequence 5'-3'
**ath-miR-416 (control miRNA)**	5'-GGUUCGUACGUACACUGUUCA -3'
**cel-miR-243-3p (control miRNA)**	5'-CGGUACGAUCGCGGCGGGAUAUC -3'
**mmu-miR-3535**	5'-CUGAAGCUCAGAGGGCUCUGAU -3'
**hsa-miR-155-5p**	5'-UUAAUGCUAAUCGUGAUAGGGGUU-3'
**hsa-miR-9-5p**	5'UCUUUGGUUAUCUAGCUGUAUGA--3'

Table S3. List of Small molecule inhibitors used in this study, including known targets, reported IC50 values, and applied working concentrations.

Name	Known target(s)	IC_{50} values	Final conc.	Manufacturer	Cat. No.
**10074-G5**	MYC	15.6 µM Daudi cells, 13.5 µM HL-60 cells	10 µM	Hözel Diagnostika	SIW-S3686-5mg
**A1517499**	STAT6	21 nM in cell-free assay	1 µM	Sigma	SML1906-5MG
**Stattic**	STAT3	5.1 µM in cell-free assay, 20 µM in Panc-2 and HPAC cells	5 µM	Merck	573099-25MG
**Mocetinostat**	HDAC1/2/3/11	0.15 µM for HDAC1, 0.29 µM for HDAC2, 1.66 µM for HDAC3, 0.59 µM for HDAC11 in cell-free assay, 0.4 – 38 µM in various human cell lines	2 µM	Hölzel Diagnostika	TMO-T2512-5mg
**Entinostat**	HDAC1/2/3	0.163 µM for HDAC1, 0.396 µM for HDAC2, 0.605 µM for HDAC3 in cell-free assay	0.5 µM	Biomol	BPS-27011
**RGFP966**	HDAC3	80 nM in cell-free assay	80 nM	Biomol	Cay16917-1

Table S4. Enriched GO terms and pathways for significantly inhibited genes by miR-155-5p and miR-3535 in MDA-MB-231 cells.

Term	p value	Genes
**bounding membrane of organelle**	0.0006	VAMP4,RAB15,TMEM30A,PRRG4,CHST4,RAB23,NIPAL1,ASS1,PLEKHB2,ENPP4,SYPL1,MAL2,GOLT1B,ENTPD7,LYSMD3,ANTXR2
**endomembrane system**	0.0018	SCG2,VAMP4,RAB15,TMEM30A,PSKH1,GDPD1,CDS1,GPR85,LIPG,CHST4,RAB23,SLC40A1,NIPAL1,PLEKHB2,ENPP4,SYPL1,MAL2,GOLT1B,ENTPD7,LYSMD3,ACTR10,ANTXR2,DPY19L1
**organelle membrane**	0.0026	VAMP4,RAB15,TMEM30A,PSKH1,GDPD1,CDS1,PRRG4,CHST4,RAB23,NIPAL1,ASS1,PLEKHB2,ENPP4,SYPL1,MAL2,GOLT1B,ENTPD7,LYSMD3,ANTXR2,DPY19L1
**vesicle**	0.0065	SCG2,VAMP4,RAB15,TMEM30A,LIPG,LIFR,RAB23,SLC40A1,F11R,ASS1,PLEKHB2,ENPP4,SYPL1,NT5E,MAL2,ENTPD7,DSG2,ACTR10,ANTXR2,DDAH1
**cytoplasmic vesicle membrane**	0.0277	VAMP4,RAB15,TMEM30A,RAB23,PLEKHB2,ENPP4,SYPL1,MAL2,ENTPD7,ANTXR2
**vesicle membrane**	0.0304	VAMP4,RAB15,TMEM30A,RAB23,PLEKHB2,ENPP4,SYPL1,MAL2,ENTPD7,ANTXR2
**membrane**	0.0351	VAMP4,DCP1A,RAB15,SLC19A2,TMEM30A,PSKH1,GDPD1,CDS1,GPR85,PRRG4,LIFR,CHST4,RAB23,STC1,UAP1,SLC40A1,NIPAL1,F11R,IL1RAP,ASS1,PLEKHB2,ENPP4,TMEM237,SYPL1,NT5E,MAL2,GOLT1B,ENTPD7,LYSMD3,DSG2,ANTXR2,DPY19L1
**hsa-miR-192-5p**	0.0080	DCP1A,PHF19,SLC19A2,TMEM30A,PRRG4,ID1,RAB23,NIPAL1,IL1RAP,ENPP4,ENTPD7,LYSMD3
**hsa-miR-548p**	0.0354	GPR85,RAB23,SYPL1,ANTXR2,DDAH1

Supplementary Figures

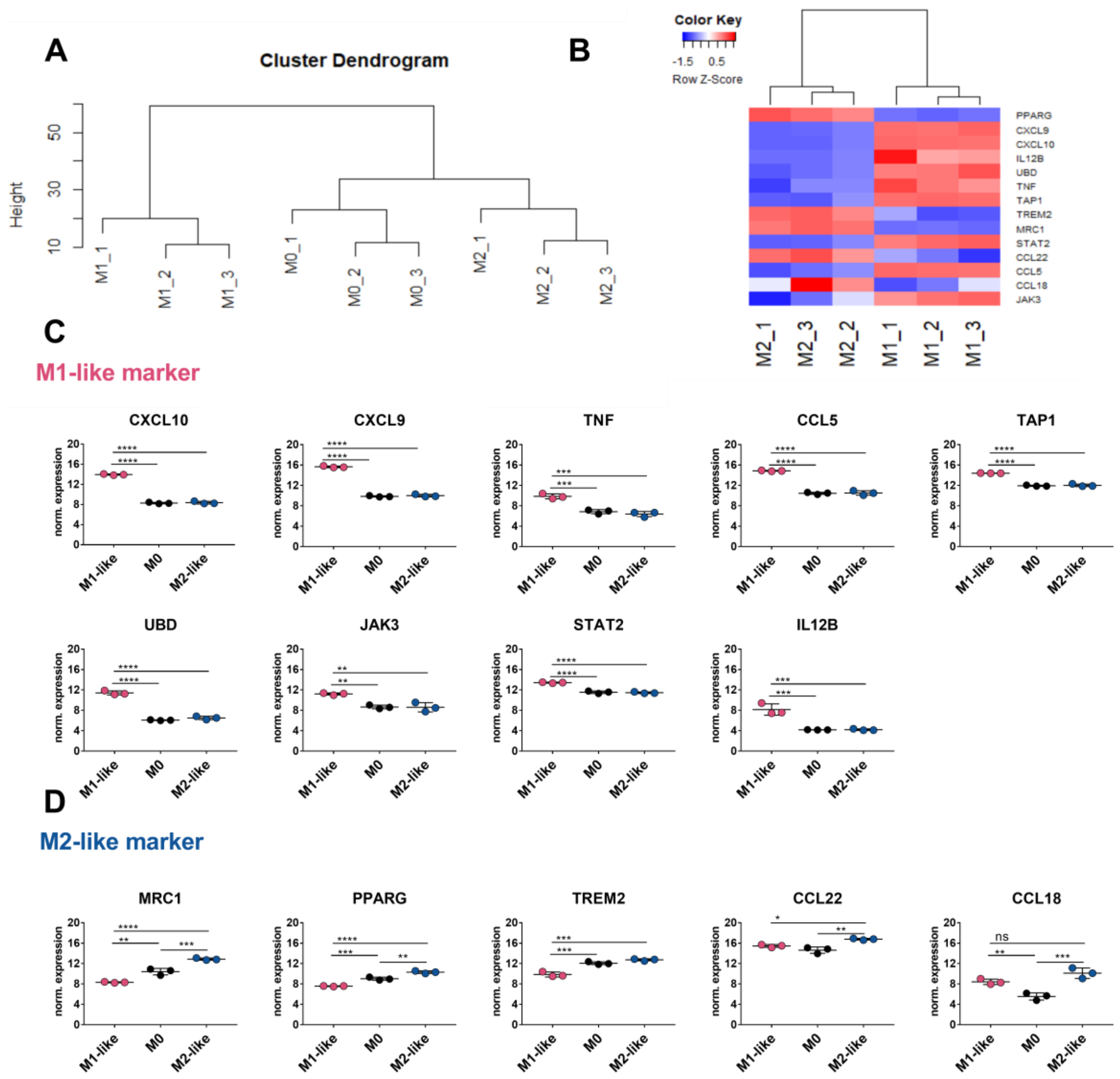

Fig. S1: Expression level of selected marker genes in human M1- and M2-like macrophages. Human monocytes were polarized with IL4 towards anti-inflammatory state (M2-like) or with LPS and IFNγ towards pro-inflammatory state (M1-like) or left untreated (M0). RNA-seq was performed 48 h after polarization. Three biological replicates were performed per condition. **A:** Clustering of samples based on 250 genes with highest variation. M2-like macrophages are more similar to the M0 state than M1-like macrophages. **B:** Heatmap of selected marker genes used later in this study to monitor macrophage polarization by qPCR and discriminate between M1- and M2-like macrophages. **C, D:** The expression of selected M1- and M2-like marker genes are shown respectively. Significance was assessed by ONE-Way ANOVA. P-values were corrected for multiple testing using Tukey's multiple comparison test. *:_p < 0.05; **: p < 0.01; ***: p < 0.001; ****: p < 0.0001; ns: p > 0.05.

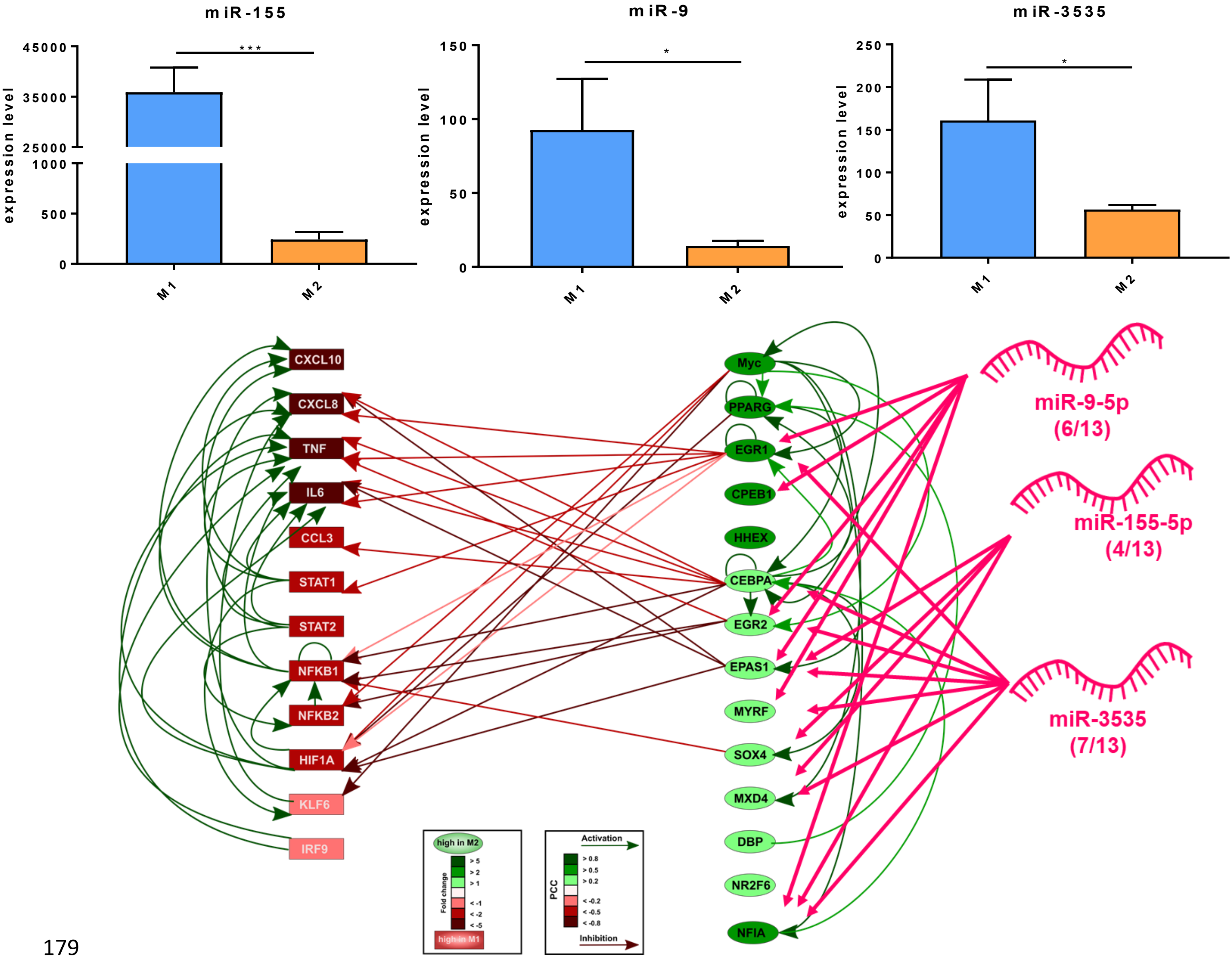

Fig. S2: miR-155, miR-9 and miR-3535 are upregulated in M1-like macrophages and are predicted to target multiple M2-regulators. A: Based on miRNA-sequencing of M1-like (HMDMs polarized with LPS/IFNγ) and M2-like (HDMs polarized with IL4) macrophages, we analyzed the expression of miR-155, miR-9 and miR-3535 between M1 and M2 cells. All three miRNAs are significantly enhanced in M1-like macrophages. **B:** Using mirRmap data base we determined M2-reguators exhibiting a binding site for miR-155-5p, miR-3535 or miR-9-5p within the 3′-UTR of the respective mRNA target molecule. All three miRNAs are predicted to target multiple M2-regulators, especially miR-3535 by targeting 7/13 M2-TFs from our regulatory network. Mean ± SD are shown. Significance was assessed by two Sample T-test. *: $p < 0.05$; **: $p < 0.01$; ***: $p < 0.001$; ****: $p < 0.0001$.

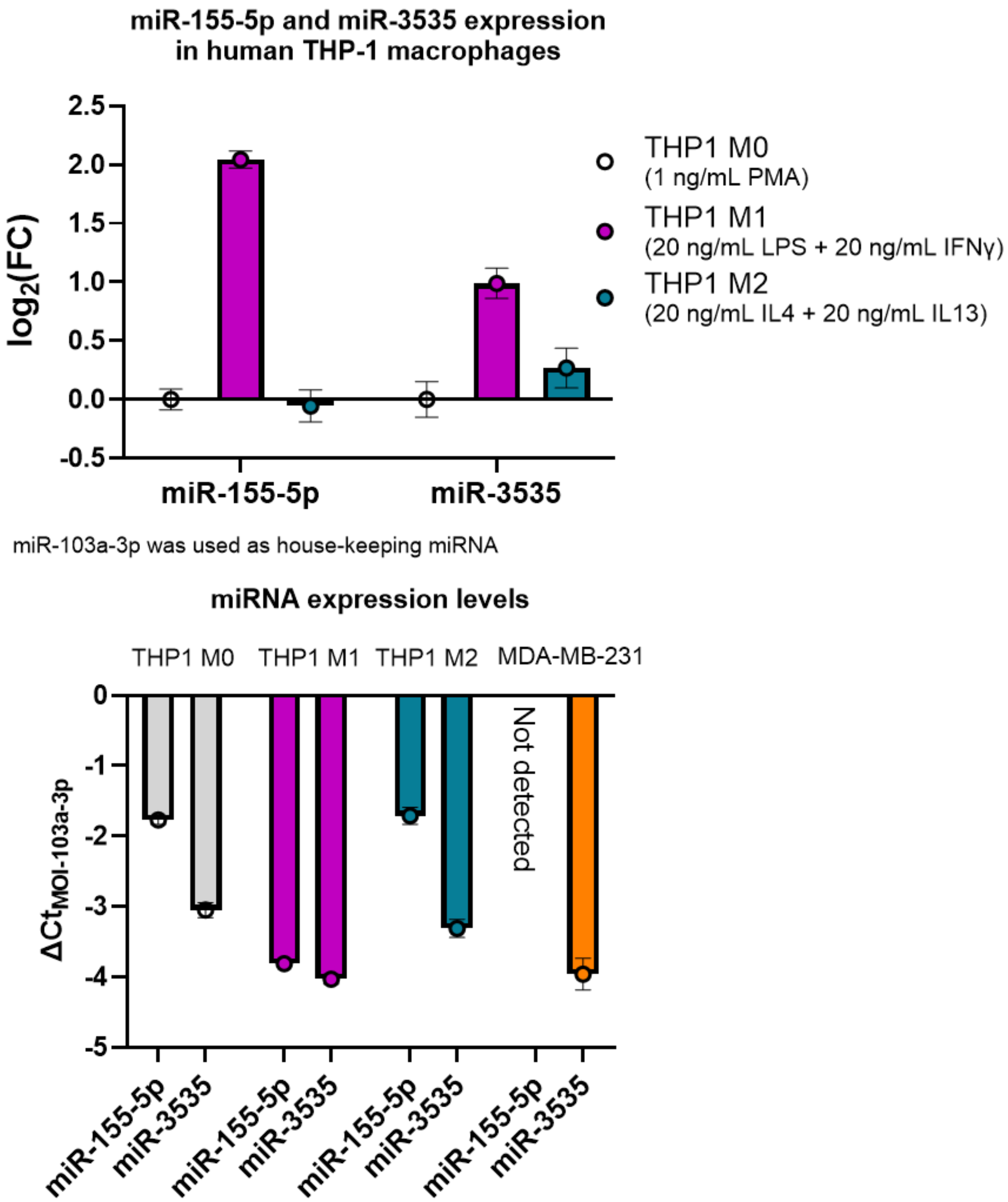

Fig. S3: Endogenous expression of miR-155-5p and miR-3535 in THP1-derived macrophages and MDA-MB-231 cells. Endogenous miRNA expression was analyzed using the TaqMan™ Advanced miRNA Assay Kit with miRNA-specific TaqMan probes. miR-103a-3p served as housekeeping miRNA for normalization. THP1-derived M0 macrophages were generated by stimulation with 1 ng/mL PMA for 72 h followed by 24 h resting without PMA. For M1 polarization, THP1-derived macrophages were stimulated with 20 ng/mL LPS and 20 ng/mL IFNγ for 48 h. M2 polarization was induced using 20 ng/mL IL-4 and 20 ng/mL IL-13 for 48 h. MDA-MB-231 cells were cultured under standard conditions. Cells were harvested and total RNA including small RNAs was isolated using the miRNeasy Kit prior to reverse transcription and qPCR analysis. MOI: miRNA of interest.

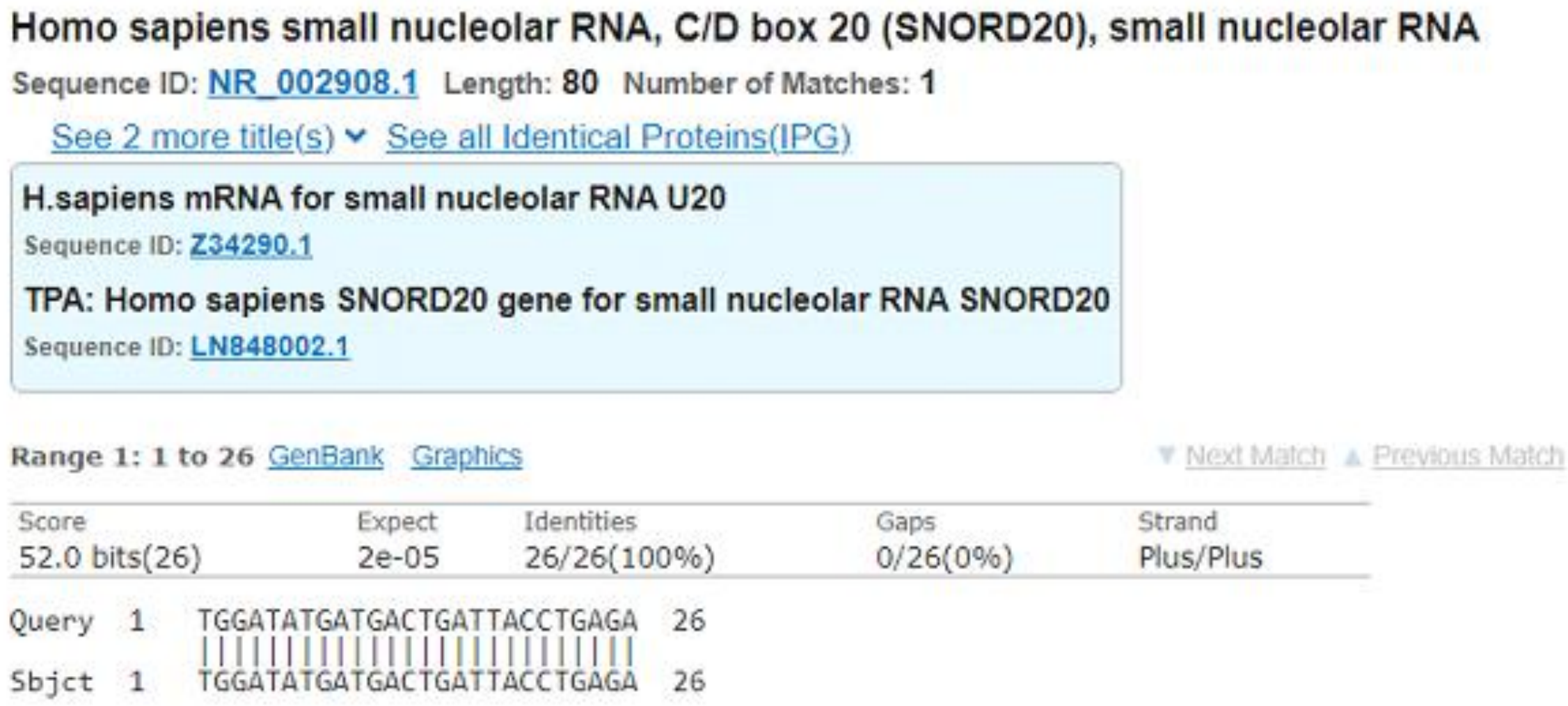

Fig. S4: BLAST alignment of murine miR-3535 with the human genome. The mature murine miR-3535 sequence (UGGAUAUGAUGACUGAUUACCUGAGA) was analyzed using NCBI BLAST against the human genome (GRCh38). The sequence shows complete alignment to the small nucleolar RNA SNORD20.

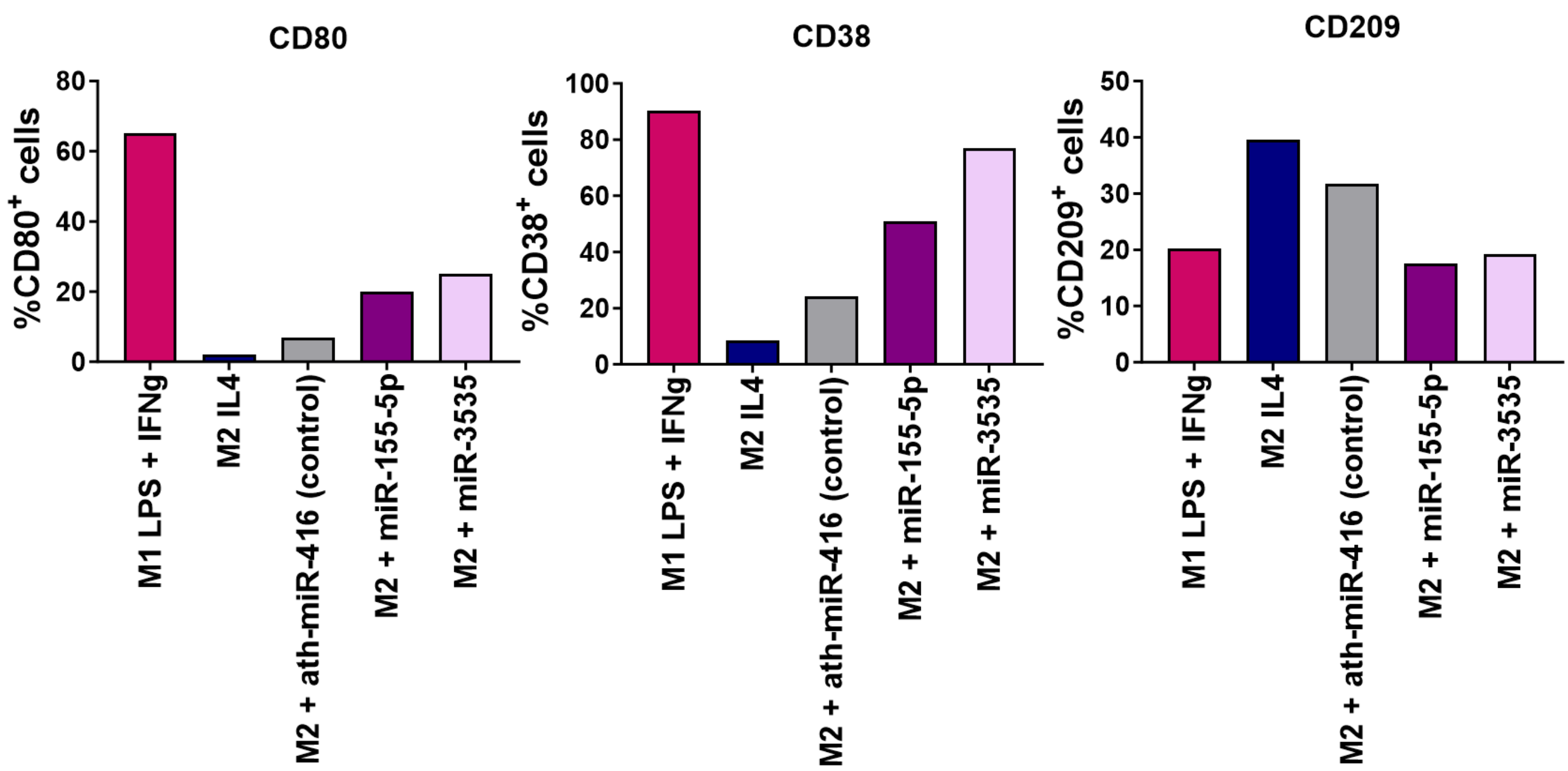

Fig. S5: miR-155 and miR-3535 enhance surface expression level of M1-like markers CD80 and CD38. HMDMs were transfected with 50 nM miRNA mimic and surface expression of M1-like markers (CD80/CD38) as well as M2-like markers (CD209) was measured 72 h post transfection (p.t.) by flow cytometry. Cells were gated through FSC/SSC and on viablecells. Percentage of marker positive cells were analyzed and compared to control transfection with ath-miR-416 and cytokine polarized macrophages.

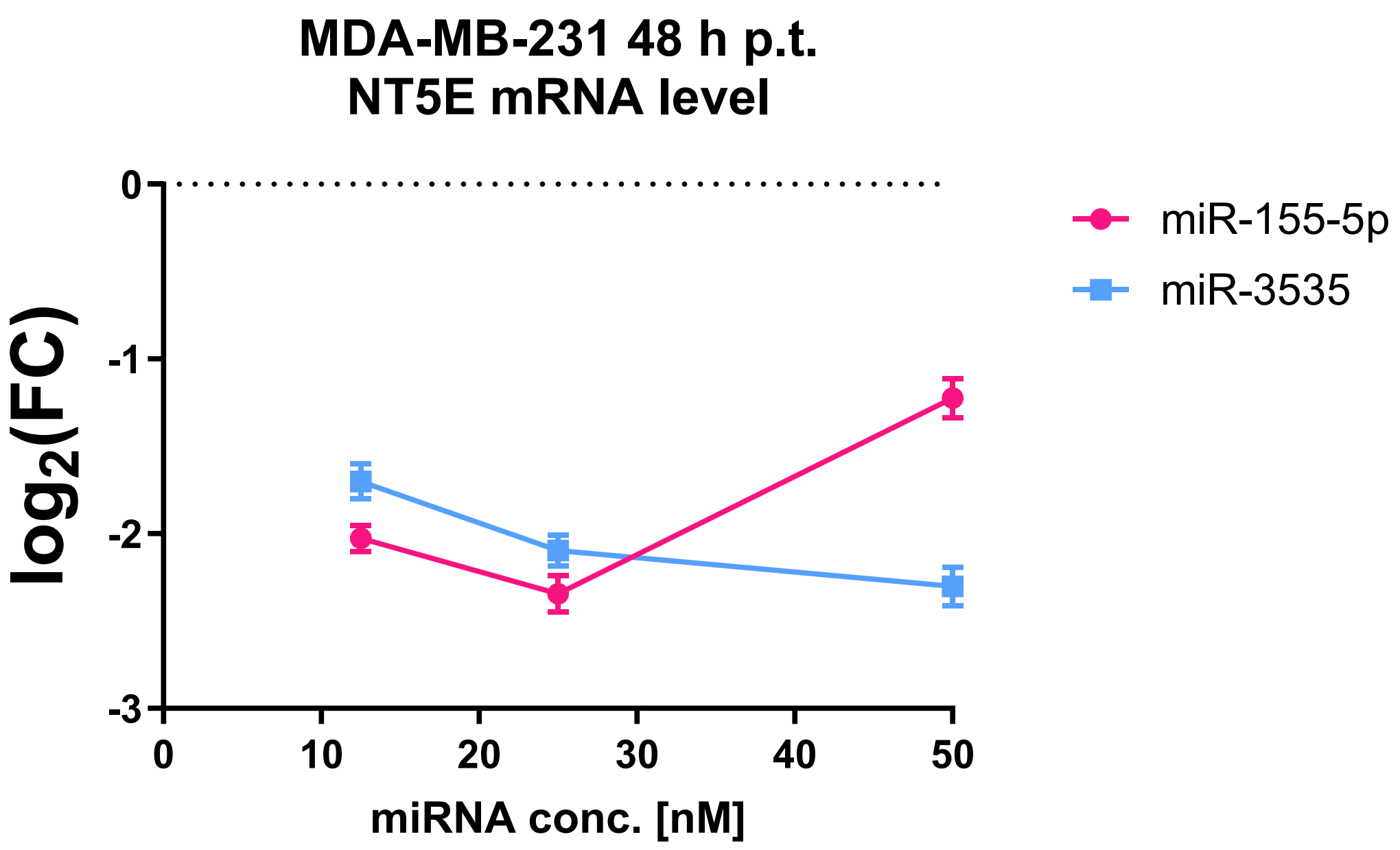

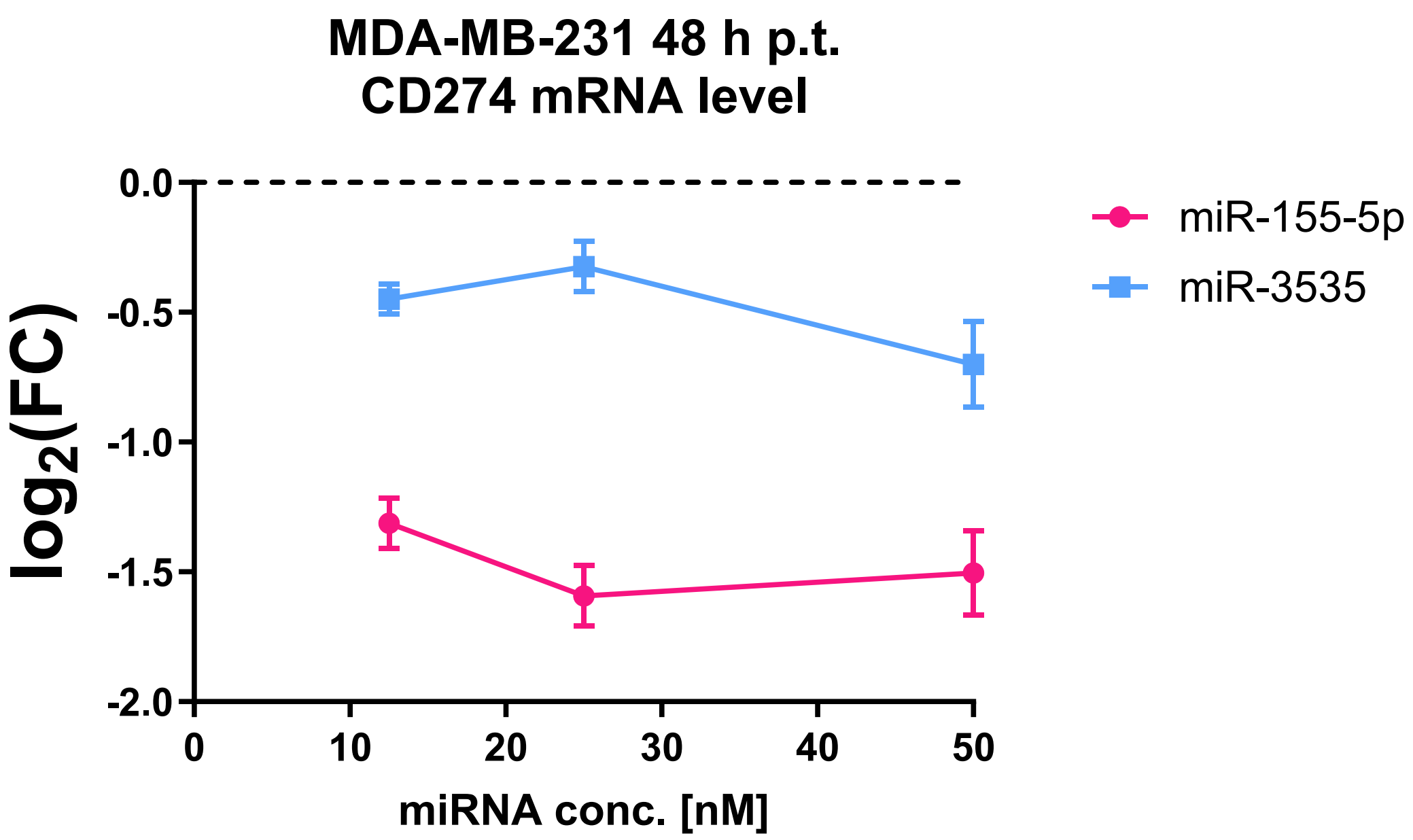

Fig. S6: Dose-dependent effects of miR-155-5p and miR-3535 on immune checkpoint expression in MDA-MB-231 cells. Cells were transfected with 12.5 nM, 25 nM, or 50 nM miRNA mimics and harvested after 48 h. NT5E and CD274 mRNA expression levels were analyzed by qPCR and normalized to the housekeeping gene RPL19. Fold changes were calculated relative to the corresponding ath-miR-416 control miRNA at each concentration. miR-3535 exhibited a concentration-dependent increase in target repression, whereas miR-155-5p showed a non-linear response pattern with maximal NT5E suppression at 25 nM and a plateau effect for CD274 repression from 25 nM onwards. Data are presented as mean ± SD.

Sequences downloaded from http://genome.ucsc.edu

Sequence of CD274-3'-UTR 2705 bases

UCCAGCAUUGGAACUUCUGAUCUUCAAGCAGGGAUUCUCAACCUGUGGUUUAGGGGUUCAUCGGGGCUGAGCGUGACAAGAGG
AAGGAAUGGGCCCGUGGGAUGCAGGCAAUGUGGGACUUAAAAGGCCCAAGCACUGAAAAUGGAACCUGGCGAAAGCAGAGGAG
GAGAAUGAAGAAAGAUGGAGUCAAACAGGGAGCCUGGAGGGAGACCUUGAUACUUUCAAAUGCCUGAGGGGCUCAUCGACGCC
UGUGACAGGGAGAAAGGAUACUUCUGAACAAGGAGCCUCCAAGCAAAUCAUCCAUUGCUCAUCCUAGGAAGACGGGUUGAGAA
UCCCUAAUUUGAGGGUCAGUUCCUGCAGAAGUGCCCUUUGCCUCCACUCAAUGCCUCAAUUUGUUUUCUGCAUGACUGAGAGU
CUCAGUGUUGGAACGGGACAGUAUUUAUGUAUGAGUUUUUCCUAUUUAUUUUGAGUCUGUGAGGUCUUCUUGUCAUGUGAGUG
UGGUUGUGAAUGAUUUCUUUUGAAGAUAUAUUGUAGUAGAUGUUACAAUUUUGUCGCCAAACUAAACUUGCUGCUUAAUGAUU
UGCUCACAUCUAGUAAAACAUGGAGUAUUUGUAAGGUGCUUGGUCUCCUCUAUAACUACAAGUAUACAUUGGAAGCAUAAAGA
UCAAACCGUUGGUUGCAUAGGAUGUCACCUUUAUUUAACCCAUUAAUACUCUGGUUGACCUAAUCUUAUUCUCAGACCUCAAG
UGUCUGUGCAGUAUCUGUUCCAUUUAAAUAUCAGCUUUACAAUUAUGUGGUAGCCUACACACAUAAUCUCAUUUCAUCGCUGU
AACCACCCUGUUGUGAUAACCACUAUUAUUUUACCCAUCGUACAGCUGAGGAAGCAAACAGAUUAAGUAACUUGCCCAAACCA
GUAAAUAGCAGACCUCAGACUGCCACCCACUGUCCUUUUAUAAUACAAUUUACAGCUAUAUUUUACUUUAAGCAAUUCUUUUA
UUCAAAAACCAUUUAUUAAGUGCCCUUGCAAUAUCAAUCGCUGUGCCAGGCAUUGAAUCUACAGAUGUGAGCAAGACAAAGUA
CCUGUCCUCAAGGAGCUCAUAGUAUAAUGAGGAGAUUAACAAGAAAAUGUAUUAUUACAAUUUAGUCCAGUGUCAUAGCAUAA
GGAUGAUGCGAGGGGAAAACCCGAGCAGUGUUGCCAAGAGGAGGAAAUAGGCCAAUGUGGUCUGGGACGGUUGGAUAUACUUA
AACAUCUUAAUAAUCAGAGUAAUUUUCAUUUACAAAGAGAGGUCGGUACUUAAAAUAACCCUGAAAAAUAACACUGGAAUUCC
UUUUCU**AGCAUUA**UAUUUAUUCCUGAUUUGCCUUUGCCAUAUAAUCUAAUGCUUGUUUAUAUAGUGUCUGGUAUUGUUUAACA
GUUCUGUCUUUUCUAUUUAAAUGCCACUAAAUUUUAAAUUCAUACCUUUCCAUGAUUCAAAAUUCAAAAGAUCCCAUGGGAGA
UGGUUGGAAAAUCUCCACUUCAUCCUCCAAGCCAUUCAAGUUUCCUUUCCAGAAGCAACUGCUACUGCCUUUCAUUCAUAUGU
UCUUCUAAAGAUAGUCUACAUUUGGAAAUGUAUGUUAAAAGCACGUAUUUUUAAAAUUUUUUUCCUAAAUAGUAACACAUUGU
AUGUCUGCUGUGUACUUUGCUAUUUUUAUUUAUUUUAGUGUUUCUUAUAUAGCAGAUGGAAUGAAUUUGAAGUUCCCAGGGCU
GAGGAUCCAUGCCUUCUUUGUUUCUAAGUUAUCUUUCCCAUAGCUUUUCAUUAUCUUUCAUAUGAUCCAGUAUAUGUUAAAUA
U**GUCCUA**CAUAUACAUUUAGACAACCACCAUUUGUUAAGUAUUUGCUCUAGGACAGAGUUUGGAUUUGUUUAUGUUUGCUCAA
AAGGAGACCCAUGGGCUCUCCAGGGUGCACUGAGUCAAUCUA**GUCCUA**AAAAGCAAUCUUAUUAUUAACUCUGUAUGACAGAA
UCAUGUCUGGAACUUUUGUUUUCUGCUUUCUGUCAAGUAUAAACUUCACUUUGAUGCUGUACUUGCAAAAUCACAUUUUCUUU
CUGGAAAUUCCGGCAGUGUACCUUGACUGCUAGCUACCCUGUGCCAGAAAAGCCUCAUUCGUUGUGCUUGAACCCUUGAAUGC
CACCAGCUGUCAUCACUACACAGCCCUCCUAAGAGGCUUCCUGGAGGUUUCGAGAUUCAGAUGCCCUGGGAGAUCCCAGAGUU
UCCUUUCCCUCUUGGCCAUAUUCUGGUGUCAAUGACAAGGAGUACCUUGGCUUUGCCACAUGUCAAGGCUGAAGAAACAGUGU
CUCCAACAGAGCUCCUUGUGUUAUCUGUUUGUACAUGUGCAUUUGUACAGUAAUUGGUGUGACAGUGUUCUUUGUGUGAAUUA
CAGGCAAGAAUUGUGGCUGAGCAAGGCACAUAGUCUACUCAGUCUAUUCCUAA**GUCCUA**ACUCCUCCUUGUGGUGUUGGAUUU
GUAAGGCACUUUAUCCCUUUUGUCUCAUGUUUCAUCGUAAAUGGCAUAGGCAGAGAUGAUACCUAAUUCUGCAUUUGAUUGUC
ACUUUUUGUACCU**GCAUUA**AUUUAAUAAAAUAUUCUUAUUUAUUUUGUUACUUGGUACACCAGCAUGUCCAUUUUCUUGUUUA
UUUUGUGUUUAAUAAAAUGUUCAGUUUAACAUCCCAGUGGAGAAAGUUA

miR-3535: 5'- UGGAUAUGAUGACUGAUUACCUGAGA -3' → reverse compliment seq: CAUAUCC

miR-155-5p: 5'-UUAAUGCUAAUCGUGAUAGGGGUU -3' → reverse compliment seq: AGCAUUA

Sequence of NT5E-3'-UTR 1774 bases

CCAAAAAUUCUCCUUGCCUUUAAUGUGUGAAACUGCAUUUUUUCAAGUGAGAUUCAAAUCUGCCUUUUAGGACCUGGCUUUGU
GACAGCAAAAACCAUCUUUACAGGCUCCUAGAAGCUGAAGGUUAGAGCAUUAUAAAAUGAAGAGACAGACAUGAUUACUCAGG
GUCAGCAACCUAGUGAGUUAGAAAAAAAAUUAACAUAGGGCCCUAUAAGGAGAAAGCCAACUAUGUUAAGUUUACGUGUCCAA
AUUUUAAUGAAAUUUUACUAACAAUUUUAAACCAUAUUUUUCUUCUUCAUAUCCAUUUCUAAUCCAUCAAACAGCUUAUGUUU
ACAUAAAAUUUUAUCAUUCACAAGGAAGUUUUAAGCACACUGUCUCAUUUGAUAUCCACAACUUAUUUUUGGUAGGAAAGAGA
GAUGUUUUUCCCACCUGUCAGAUGAAAAAACUGAAGCUCAAAAAGGGUUGACUUGACCAUACAGCUAAUGCUGACAGAUCCAA
GACCUAGACCUAGGUCUUUUGAACUCAAGUCCAGCAUUCUCAACUAUAUCAAGUUACUGUUCAGAAUACUUAAUAUCUCCUCU
CUUCAUAAUUAUCAAUAGCCCCAAGCUCAUGGAUGACAAAUCUCUGCUUUAUUUCUUGUCUCUAUUUUUUCACUUUAUAGCUC
CUGUUAUAAUAGCAAGUUUAAUGGUAUAAACACAGGAUACCAUCCUCUCUUGCAACACCCAUGUGCCUUUGAUGAGUCAGGUA
GCAAGCUGUAGUAGAUAAUGAGAAAGGCCAGAGGCUGCAAAAGACAGUCAAAGGACACGAGAGAAAGGAAGGGGAAGAACAGG
ACUCCAGGACUGUUUUAUAUUAUAGAAAAGCAAGAGCUAAAGAGCAUUUACACAUGUUAAACAGAUACUUGUUAAGCAUAGUG
CCUGACACACGGCAUUAGCUGUUAUUUUAUGAGAUUCCAUCAGCUCUGCCUCUGUCCUCUUUCUUCUAACAUGAAGGUAUCAU
GAGAAGAGAACCUUCUAACAUAAGCUGUAAUUCUAAACCUGCACUUGUCCCUCUCCAGCAAGAGGCUAGCACUGAAUUCAUUC
UACUCAUACUACACACCCAGUUAUGGAAUGUCCAGAGUUCUCGAAGAAAAUAAAUGACUUUAGGAAGAGGUAUACAUUUUUUA
AGUCGCUCUGCCUCCAAAUCUGAACAGUCACUGUAAAUCAUUCUUAAGCCCAGAUAUGAGAACUUCUGCUGGAAAGUGGGACC
CUCUGAGUGGGUGGUCAGAAAAUACCCAUGCUGAUGAAAUGACCUAUGCCCAAAGAACAAAUACUUAACGUGGGAGUGGAACC
ACAUGAGCCUGCUCAGCUCUGCAUAAGUAAUUCAAGAAAUGGGAGGCUUCACCUUAAAAACAGUGUGCAAAUGGCAGCUAGAG
GUUUUGAUAGGAAGUAUGUUUGUUUCUUAGUGUUUACAAAUAUUAAGUACUCUUGAUACAAAAUAUACUUUUAAACUUCAUAA
CCUUUUUAUAAAAGUUGUUGCAGCAAAAUAAUAGCCUCGGUUCUAUGCAUAUAUGGAUUAGCUAUAAAAAAUGUCAAUAAGAU
UGUACAAGGAAAAUUAGAGAAAGUCACAUUUAGGGUUUAUUUUUUACACUUGGCCAGUAAAAUAGGGUAAAUCCUAUUAGAAU
UUUUUAAAGAACUUUUUUUAAGUUUCCUAAAUCUGUGUGUGUAUUGUGAAGUGGUAUAAGAAAUGACUUUGAACCACUUUGCA
AUUGUAGAUUCCCAACAAUAAAAUUGAAGAU

Fig. S7: *In silico* prediction of miRNA binding sites within the 3'UTRs of the NT5E and CD274 mRNA molecules. Computational target analysis (miRmap and seed scanning) identified multiple putative binding sites for the pan-functional miRNAs. For NT5E (CD73), miR-155-5p and miR-3535 each harbor two predicted binding sites within the 3'UTR (canonical seed matches). For CD274 (PD-L1), miR-155-5p shows two predicted sites, whereas miR-3535 contains only a weaker, non-canonical (≈5-mer) seed match. Schematic maps indicate site positions along the 3'UTR.

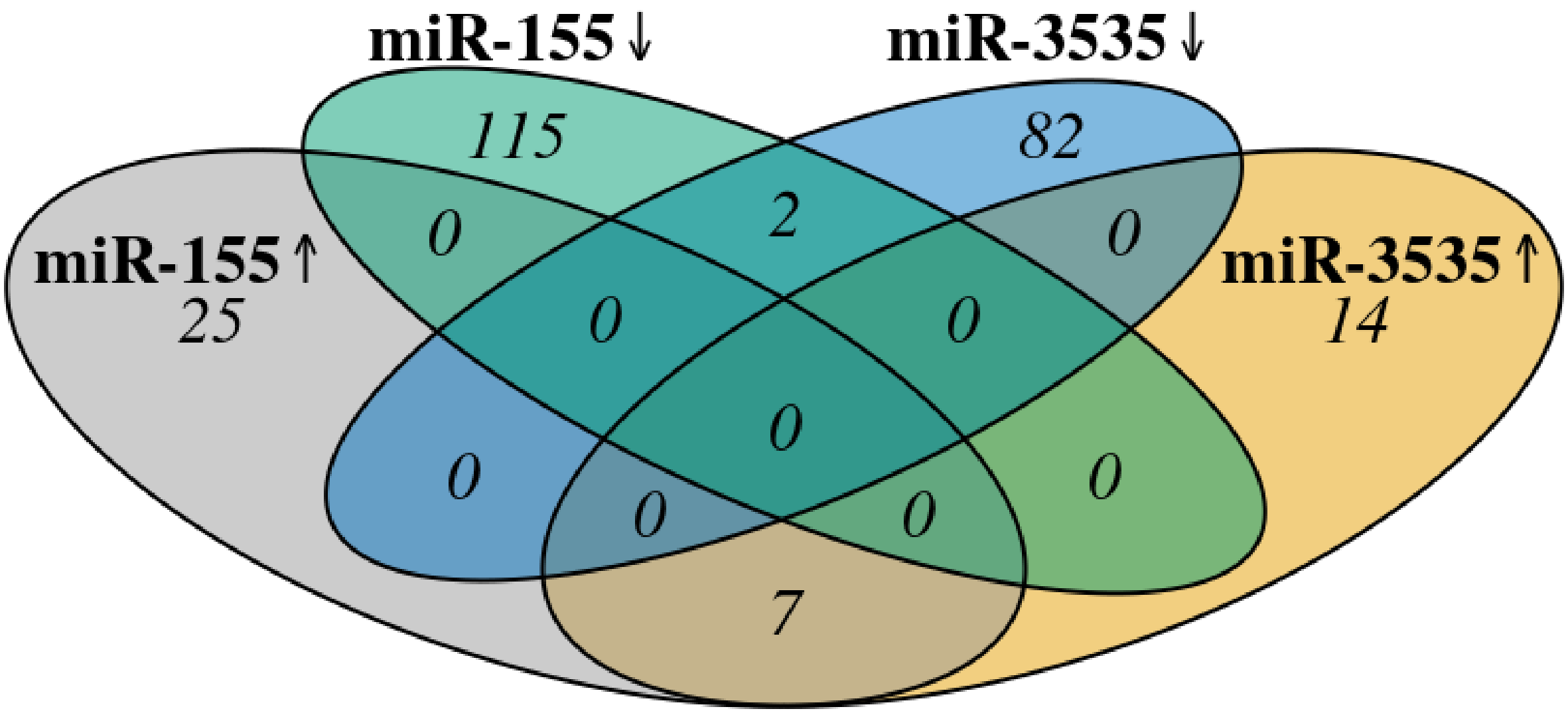

Names	total	elements
miR-155 up miR-3535 up	7	CDCA7L HDDC2 WBP1L PUDP SGMS2 NT5DC3 TNRC6B
miR-155 down miR-3535 down	2	G0S2 NT5E
miR-155 up	25	EREG MMP2 PMP2 NPIPB4 RHOB GNAQ AMPD2 IL24 LONRF1 TRIM9 PDE1C PPP2R2B CEP135 KIF21A AKAP7 PDGFRA SYT11 C1orf61 DUSP1 DPYSL3 CBS NPIPB5 CDR2 PREX1 CREB5
miR-155 down	115	POLE3 IL7R ASPH TPD52 PRKCI CFL2 SEMA3A CREG1 DDAH1 ZNF185 CCNYL1 ZSWIM6 FYTTD1 SORT1 F3 C9orf78 FEZ2 SOX9 ENTPD7 TMEM123 NR1D2 UBXN8 TRAM1 TWF1 BICD2 KCTD3 STYX PLEKHB2 TOMM34 GLUL ZMYND11 ELK3 DYRK3 PHACTR2 H3F3AP4 MICB AFF4 PLLP ASS1 SYPL1 HLTF DENND6A RCOR1 ENPP4 CBFB FAR1 PAQR3 TMEM237 ICAM1 MEST TXNDC12 CASP6 MOCS2 DUSP14 PAQR5 CDC14B LY6K MYO1D CPD ARHGAP18 RPS6KA5 IL6ST PPP2CB LAPTM5 TMEM33 GPR160 KRAS LAMP1 ACTR10 CACUL1 PEBP1 DSG2 PHC2 VAMP3 ATG3 ARL2BP SDCBP DNTTIP1 GOLT1B ARL6IP6 KPNA1 EDEM3 KLHL5 PRNP SCAMP1 PICALM HSD17B12 SLC35F6 ANTXR2 GMNN RNF26 PRKG1 MAL2 LZTFL1 SAMHD1 CLIC6 HDHD2 PRR16 CNIH1 NEGR1 TCP11L2 SSX2IP WDR45 DPY19L1 AGTRAP DR1 LYSMD3 TAPT1 EGFR TBCA CLCN3 ATP6V1C1 RFK PDE12 NECAP1
miR-3535 up	14	CALU ACVR2B RBMS3 GANAB FHL2 CRISPLD2 MNS1 FAM3C2 JAG1 GTF2E2 ATP8B1 USP46 VGLL4 GABRE
miR-3535 down	82	SEC23A EMP1 REEP3 PKIA CBX5 GCH1 NCAPH CRADD MAN2A1 SERPINE1 EIF4G2 WASL IL1RAP PLBD2 PLAU PELI2 MAN1A1 HIPK3 TMEM170A STC1 SHCBP1 SLC31A2 SEC24A PTGFRN ANKRD46 CPNE3 TMEM104 ETV1 UAP1 UBE2B MGLL SMARCD1 JOSD1 GPR3 TMEM38B GPD2 KDELR3 DIRC2 VANGL1 FBXL20 CRYZ SOAT1 PHTF2 BNIP3L RDH11 DESI2 CAND1 PRRG4 TRIM44 SIGMAR1 PPP1R15B OCRL ARSB HBP1 MAPK1IP1L OSGIN2 TMEM41B APAF1 FBXL17 CTPS2 FTO ACSL4 TWSG1 FOXRED2 NOV FLRT2 SLC35D1 LIPA NEDD4 C5orf28 ACER3 LRRC8D SC5D LUZP6 MBNL3 ARL6 ANKRD13C CBR1 NIPAL1 ADGRG1 IL11 SWAP70

Names	total	elements
in silico targets miR-155 down	174	EPB41L4B RPS6KA5 WDR45 ETNK2 TOMM34 PDP1 LZTFL1 HDHD2 TRMT6 ATP6V1C1 ENTPD7 SOX9 OAS1 PAQR3 NEGR1 NT5E NR1D2 ATG3 PKP2 ANXA2 SAMHD1 SCG2 PCDH20 SESN1 PLEKHB2 SWSAP1 ATXN1L PSKH1 CLCN3 CDC37 BICD2 KBTBD2 EHD1 KLHL5 CNIH1 TMEM135 SLC27A2 HMGCS1 KCTD3 MYO1D TBC1D23 CCDC126 PEBP1 ADD3 ENPP4 GPR137B MSL3 SLC35F6 DDAH1 SLC12A6 ANTXR2 KIAA0196 STC1 EFNA1 CSNK1G2 PHF19 CACUL1 PHC2 ZSWIM6 ALDH7A1 MTUS1 GDPD1 PKN2 GTPBP8 UBXN8 ARHGAP18 CACNB4 FAM105A SACM1L FAR1 FAM69A IL6ST DNTTIP1 MRS2 RANBP10 GLIPR1 HSD17B12 TCP11L2 F3 FEZ2 GTF2H3 GOLT1B CLIC6 ELMOD1 PDE12 C9orf78 GOT1 CASP6 ACTR10 DCP1A PRR16 POLE3 SORT1 TRAM1 CDS1 TMEM33 ARL2BP ARL6IP6 LRRC42 DUSP14 UBE2D3 TXNDC12 PDK4 PTTG1 WEE1 MYO1E CARHSP1 INPP5F H3F3A DYRK3 RCOR1 DAZAP2 NECAP1 MAPK13 RAB34 LYSMD3 GPR85 OSBPL2 SYPL1 SLC35F2 FAM210B CD47 ZMPSTE24 ERRFI1 ELK3 MOCS2 USP28 EDEM3 RUFY2 ZNF652 SAMD12 CSGALNACT2 MFAP3 SEMA3A TWF1 RAB11FIP2 DSG2 HSPA4L FBXO33 STK38L AFF4 PPP2CB PCYOX1 OSTM1 CCNYL1 IL1RAP RP2 CSNK1A1 KPNA1 TPD52 COMMD4 RNF26 CREG1 LIMCH1 CCNG1 SCRN3 SCAMP1 DR1 RAB5C TMEM144 CEP41 FYTTD1 BACH1 ZMYND11 TMEM30A SSX2IP TAPT1 UAP1 TADA2B CD274 MAL2 DPY19L1 BET1 DNAJC19

Names	total	elements
in silico targets miR-155 up	12	KIF21A ANGEL2 ABHD2 SUV39H2 FN1 HOXB3 REV3L TRIM9 CREB5 WBP1L TBPL1 RALGAPA1

Fig. S8. Microarray analysis of MDA-MB-231 cells transfected with miR-155-5p or miR-3535. Overall, expression of 1178 genes was strongly inhibited by miR-155-5p (FC < 0.5) and 84 genes for miR-3535. 32 genes were strongly upregulated by miR-155-5p (FC > 2.0) and 21 genes for miR-3535. Cells were harvested 48 h post-transfection, and differential gene expression was determined relative to control cells transfected with ath-miR-416. Three biological replicates were performed per condition. Genes were considered differentially expressed (DEGs) based on a threshold of $p < 0.05$, with fold change (FC) > 2.0 for upregulated genes and FC < 0.5 for downregulated genes. For the 306 genes significantly downregulated by miR-155-5p, we screened for potential binding sites using the previously mentioned miRNA–target databases. For 174 of these 306 genes, at least one database predicted a miR-155-5p binding site within the respective 3′-UTR.

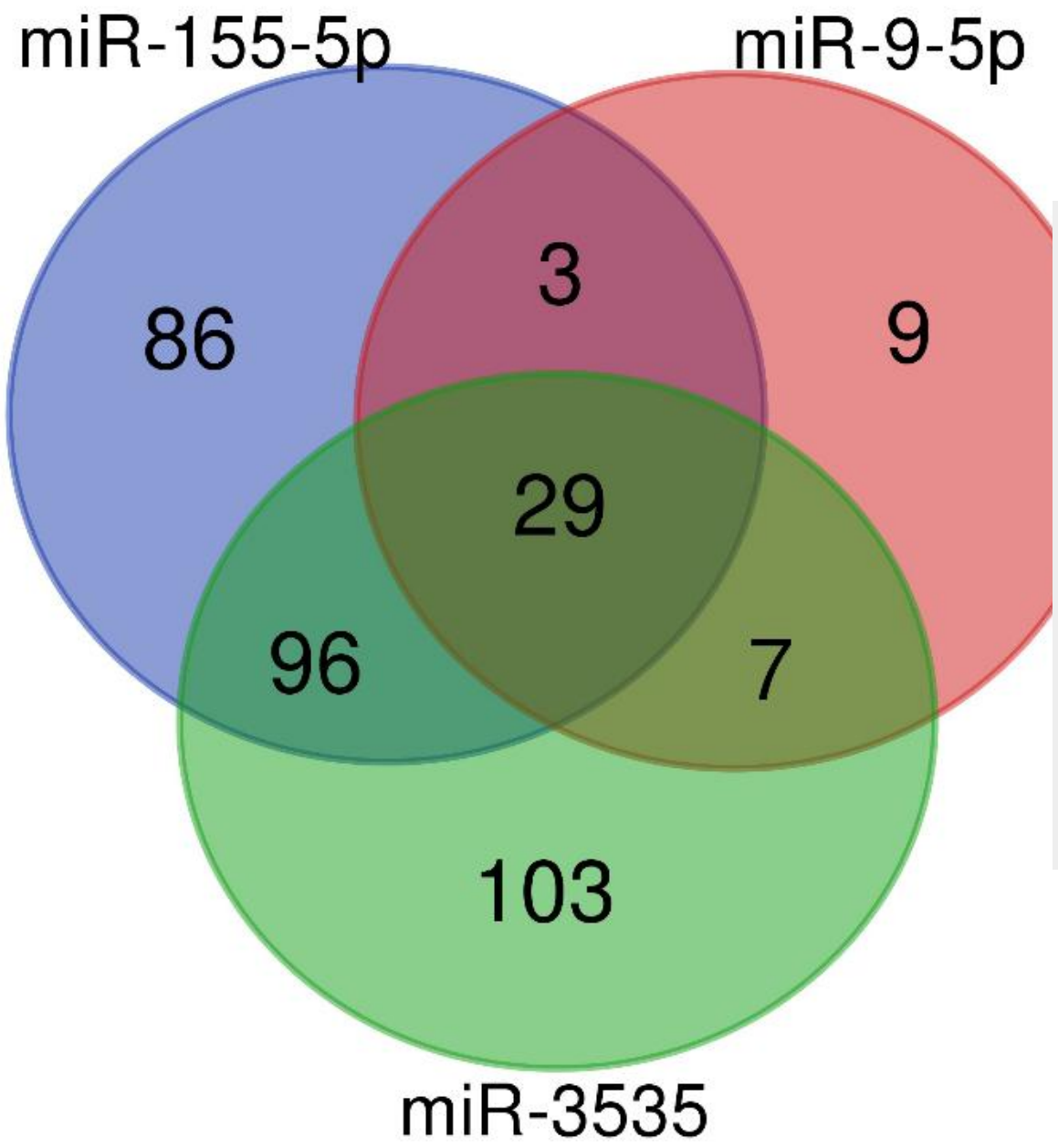

Names	total	elements
miR-155-5p miR-3535 miR-9-5p	29	IL7R TNFAIP6 IDO1 MSC IFITM3 PTGER2 SERTAD1 ADM CCL20 STX11 HCAR2 MXD1 SECTM1 SEMA4A APOBEC3A IL3RA NFKBIA HIST1H2BC ISG20 NUPR1 HIST2H2AA3 TRAF1 SPECC1L-ADORA2A HCAR3 RTP4 CCR7 RUNX2 PDE4B BIRC3
miR-155-5p miR-9-5p	3	CXCR4 PLK2 GPR132
miR-155-5p miR-3535	96	IFI35 ZC3HAV1 RGS9 CCNA1 IER2 CCL5 OAS3 HIST2H2AA4 APOBEC3D OAS1 RSAD2 ZC3H12A MX1 TNF C19orf66 PML NT5C3A TRIM22 RANBP3L DDX60 CMPK2 BCL2L11 HIST1H4H CFB NFKBIZ DDX58 CASP1 RABGAP1L CD80 CCL4L2 CCL3L3 IFIH1 MVB12A IFNL1 DUSP1 NCF1 ITGBL1 AIM2 CD38 SERPINB9 TNFRSF9 OVOL1 HIST2H4A HIST1H2BD NEURL3 USP18 IL15 SAMD9 IFI44L NUB1 IFIT1 LAMP3 HIST2H4B CCL8 TNFSF10 RARRES3 TNFSF13B GBP1 HESX1 EPSTI1 GBP5 DUSP5 IFNB1 OAS2 SP140 CSRNP1 CRLF2 DHX58 IFITM1 UBD CCL3 FBXO32 TAP1 ZBP1 PMAIP1 APOL3 CD274 OASL TNFAIP3 GBP4 USP41 HSH2D CXCL11 BANP APOBEC3B C21orf91 IFI44 WTAP PRDM1 ISG15 MX2 IFIT3 ARID5A IFIT2 IFI27 IFITM2
miR-3535 miR-9-5p	7	FPR3 CLEC4E TMEM150B CMKLR1 SLC2A6 PSTPIP2 MVB12B

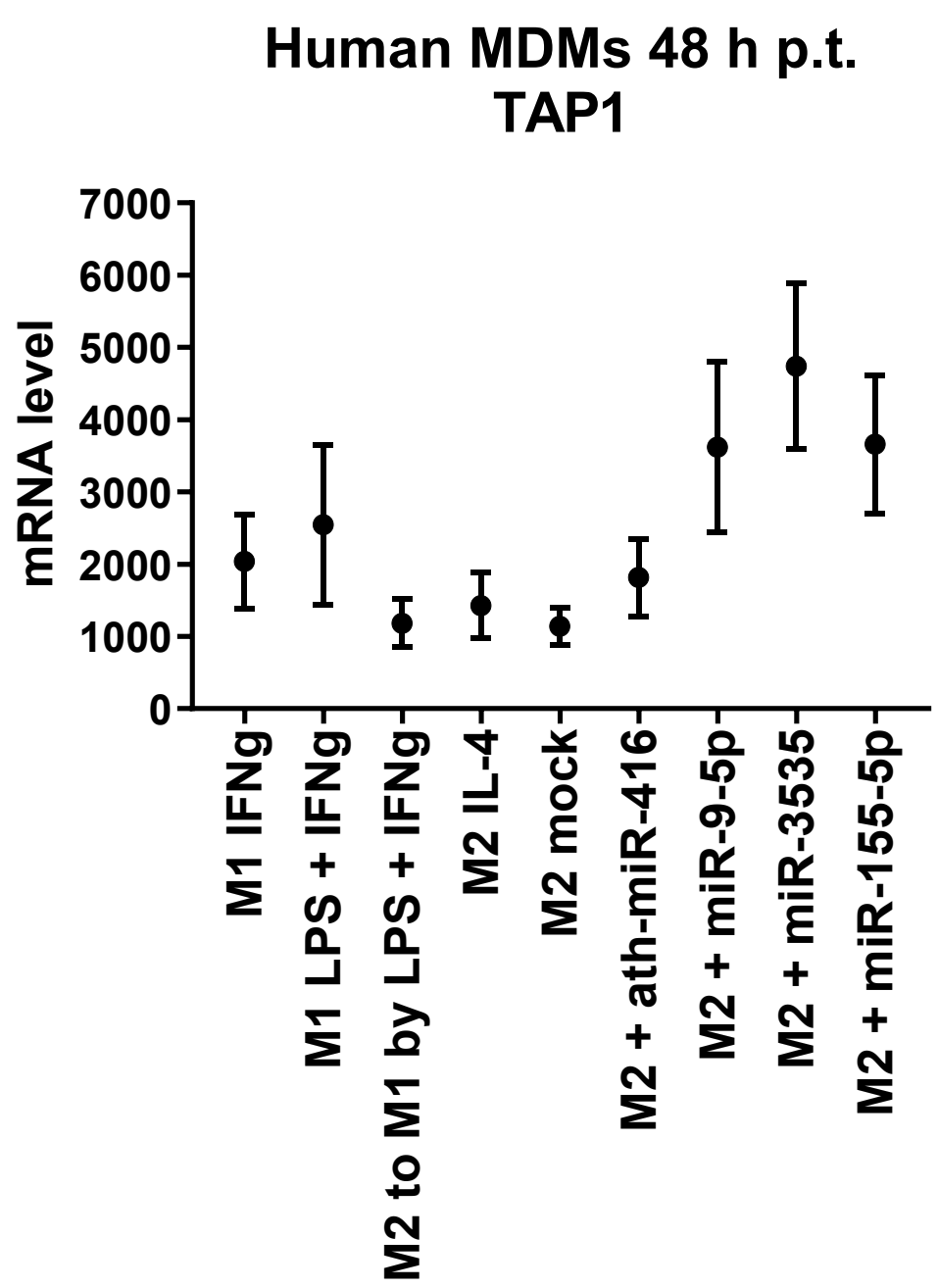

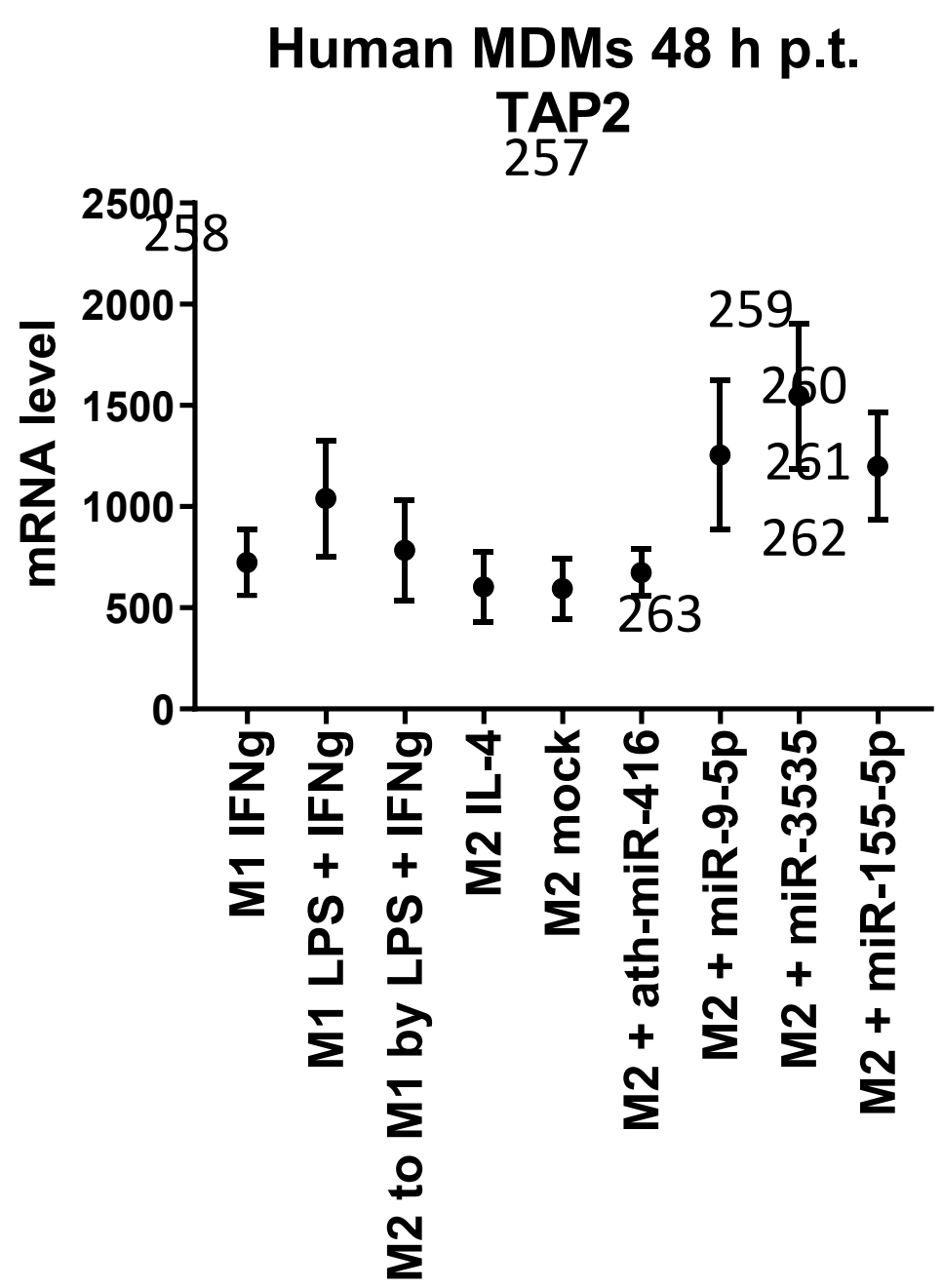

Fig. S9: Overlap of significantly upregulated genes in M2-like polarized macrophages transfected with pan-functional miRNAs.

Venn diagram showing the overlap of transcripts upregulated (fold change > 2.0) in M2-like polarized human macrophages transfected with miR-155-5p, miR-3535, or miR-9-5p, compared to control miRNA (ath-miR-416). 29 genes were commonly induced by all three miRNAs, suggesting a shared transcriptional program associated with M1-like repolarization. Notably, TAP1, which encodes an ATP-binding cassette transporter essential for MHC class I antigen processing and presentation, was significantly upregulated by both miR-155-5p and miR-3535 (FC > 2.0). To note, miR-3535 also enhanced TAP2 expression.

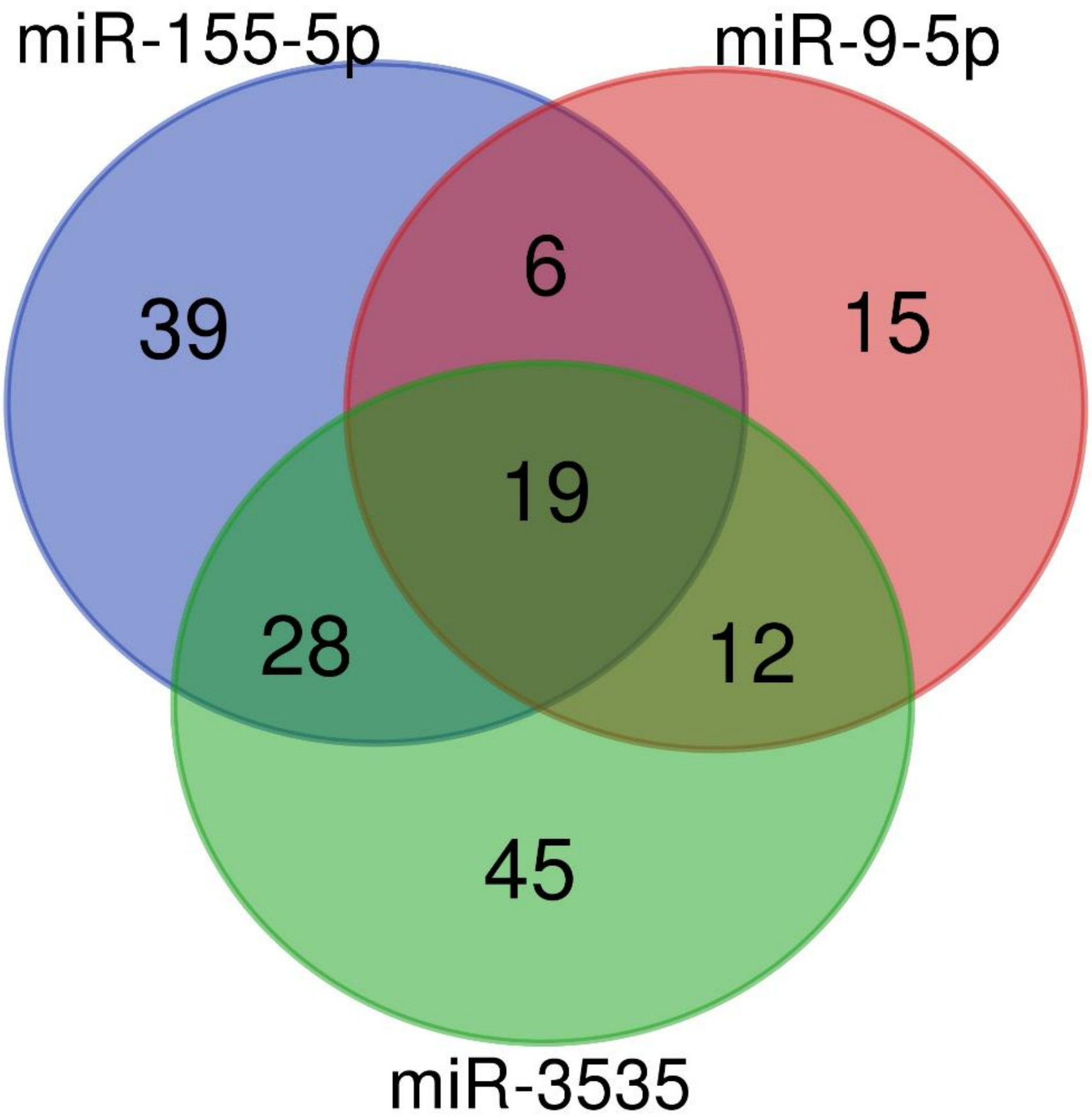

Names	total	elements
miR-155-5p miR-3535 miR-9-5p	19	CD180 SORT1 MKI67 TDRD9 PRKACB GPR34 TGFBR2 LDHB FCGR3B MYO1D ADRA2B NNT MMP12 ANOS1 HPGDS CAB39 DCBLD2 NUP210 S100A9
miR-155-5p miR-9-5p	6	ESYT1 KCNJ1 SGMS2 FCGR1A KALRN FLNB
miR-155-5p miR-3535	28	ITGAM CDCA7L GALNT7 VCL DHCR24 HS2ST1 FABP3 CD36 AMICA1 ITGB2 TSPAN15 FAM213A ITGA3 S100A4 DUSP9 FBP1 SLAMF8 HACD3 CRYL1 ATP13A2 PLXNC1 GPX3 PPP3CA STMN1 FCGR3A DNASE2B ACO1 IFITM10
miR-3535 miR-9-5p	12	IQGAP3 DTL MAN1A1 BCAT1 SPTBN1 CERK CCNA2 ETV5 TGOLN2 RNF125 MCM6 ANLN

Fig. S10: Overlap of significantly downregulated genes in M2-like polarized macrophages transfected with pan-functional miRNAs.

Venn diagram depicting the overlap of transcripts downregulated (fold change < 0.5) in M2-like polarized human macrophages transfected with miR-155-5p, miR-3535, or miR-9-5p, compared to control miRNA (ath-miR-416). 19 genes were commonly suppressed by all three miRNAs, while additional transcript-specific effects were observed for each miRNA. These shared downregulated targets likely represent core components of the M2-associated transcriptome that are consistently repressed during miRNA-induced repolarization. Notably, only a minority of these genes are predicted to be direct targets of all three miRNAs, suggesting that secondary transcriptional reprogramming contributes to the observed suppression.

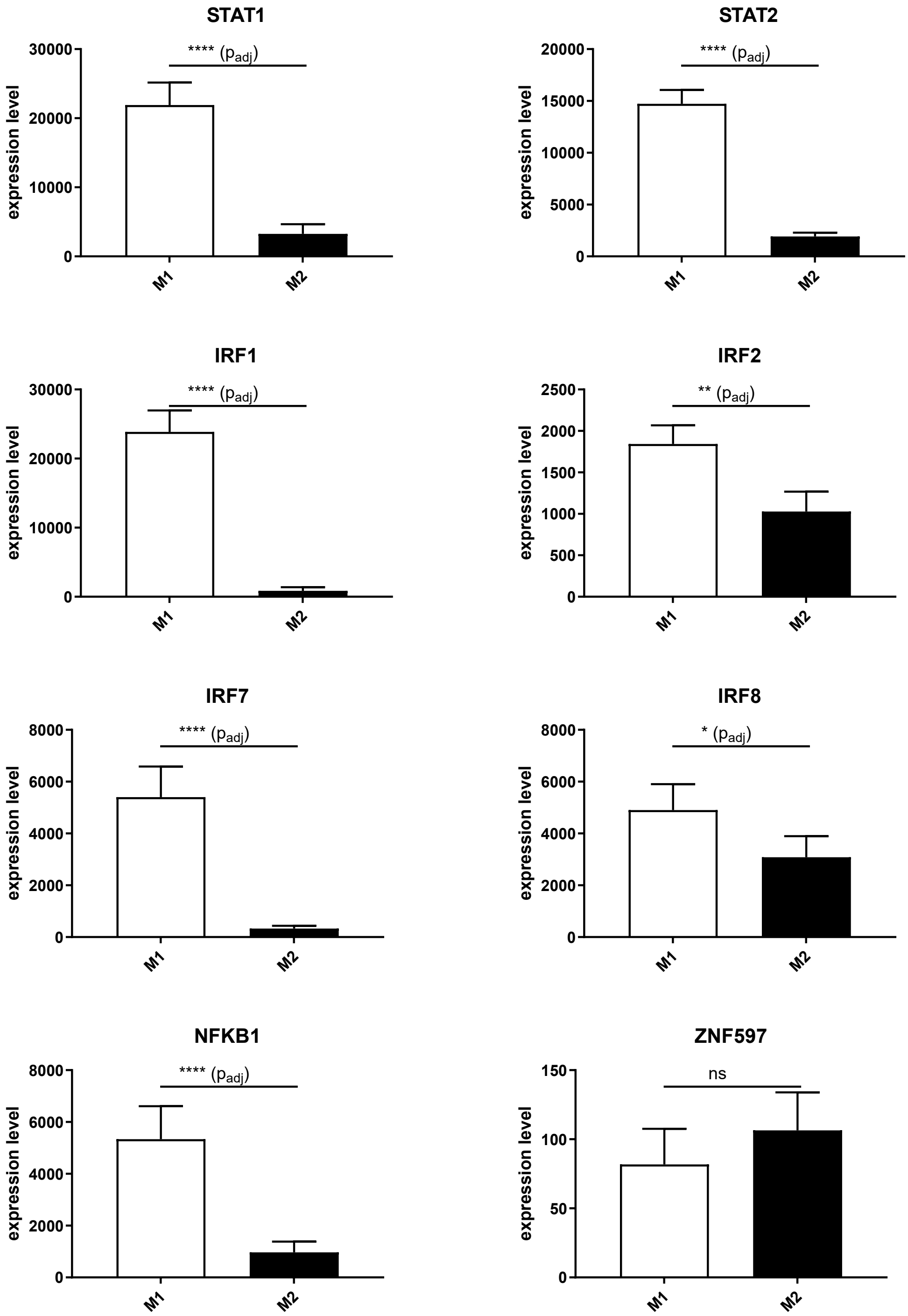

Fig. S11: Expression of selected transcription factors in M1- or M2-polarized human macrophages Gene expression levels of eight transcription factors were analyzed based on RNA-seq data from human macrophages polarized with IFN-γ and LPS (M1) or with IL-4 (M2). Seven out of eight transcription factors showed significantly higher expression in M1-polarized cells. Data are presented as mean ± SD. Statistical significance was assessed by multiple t-tests with correction for multiple comparisons. $p < 0.05$; **: $p < 0.01$; ***: $p < 0.001$; ****: $p < 0.0001$.

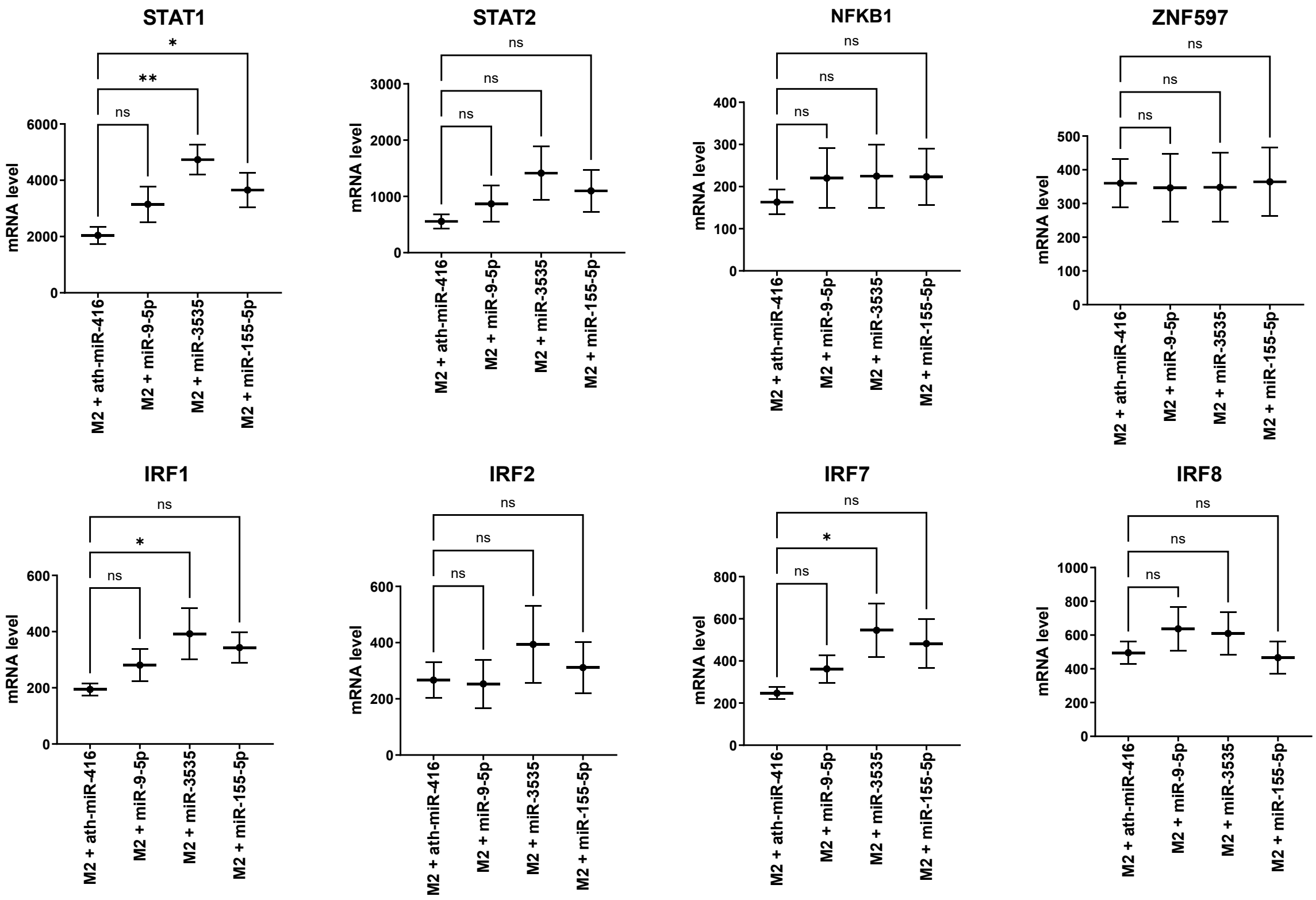

Fig. S12: Expression of selected transcription factors (TFs) in M2-like human macrophages after miRNA transfection. Human macrophages were polarized towards an M2-like state using IL-4 and subsequently transfected with either ath-miR-418 (negative control) or pan-functional miRNAs (miR-155-5p, miR-3535, or miR-9-5p). RNA was isolated 48 h post-transfection, and expression of selected transcription factors (STAT1, IRF1, IRF7, IRF8, IRF2, STAT2, NFKB1, ZNF597) was analysed by microarray. Statistical analysis was performed using one-way ANOVA with multiple comparison correction. Significant upregulation of STAT1, IRF1 and IRF7 was observed in miR-3535-transfected macrophages compared to control. STAT1 was also significantly enhanced by miR-155-5p. Data are presented as mean ± SEM; *p < 0.05, **p < 0.01.

	Fold change Activity Scores compared to control miRNA		
**TFs**	**miR-155**	**miR-3535**	**miR-9**
**ZNF14**	3,750577	1,93825154	2,285286
**YY2**	2,311654	5,03936048	2,471517
**ZBTB25**	1,794553	1,87911091	1,567079
**ZNF345B**	1,58143	2,04843731	1,873153
**SOX14**	1,58143	2,04843731	1,873153
**IRF9**	1,63634	2,29177482	1,528413
**SIX2**	1,674298	1,63283883	1,666247
**ZNF3**	2,28074	1,52220251	1,825341
**FOXL1**	3,242469	6,16837491	2,10316
**LHX1**	1,984583	3,31111403	1,989099
**IRF7**	1,69129	2,28299207	1,601353
**HOXD1**	0,605127	0,66833852	0,642712
**HOXA5**	0,669819	0,68403624	0,706897
**JUND.FRA1**	0,579687	0,45453973	0,509071
**E2F3.DP1**	0,667949	0,61711263	0,60502
**ZNF184**	0,438999	0,50491625	0,46495
**ZNF703**	0,400017	0,45059021	0,325757
**SLC2A4RG**	0,608908	0,68073139	0,690946
**HOXB3**	0,624704	0,63777125	0,654726
**HOXD10**	0,665537	0,70825558	0,6892
**HOXD12**	0,687613	0,69261387	0,697281
**HOXA11**	0,638355	0,64847331	0,670691
**TCEAL7**	0,537848	0,69489245	0,696258

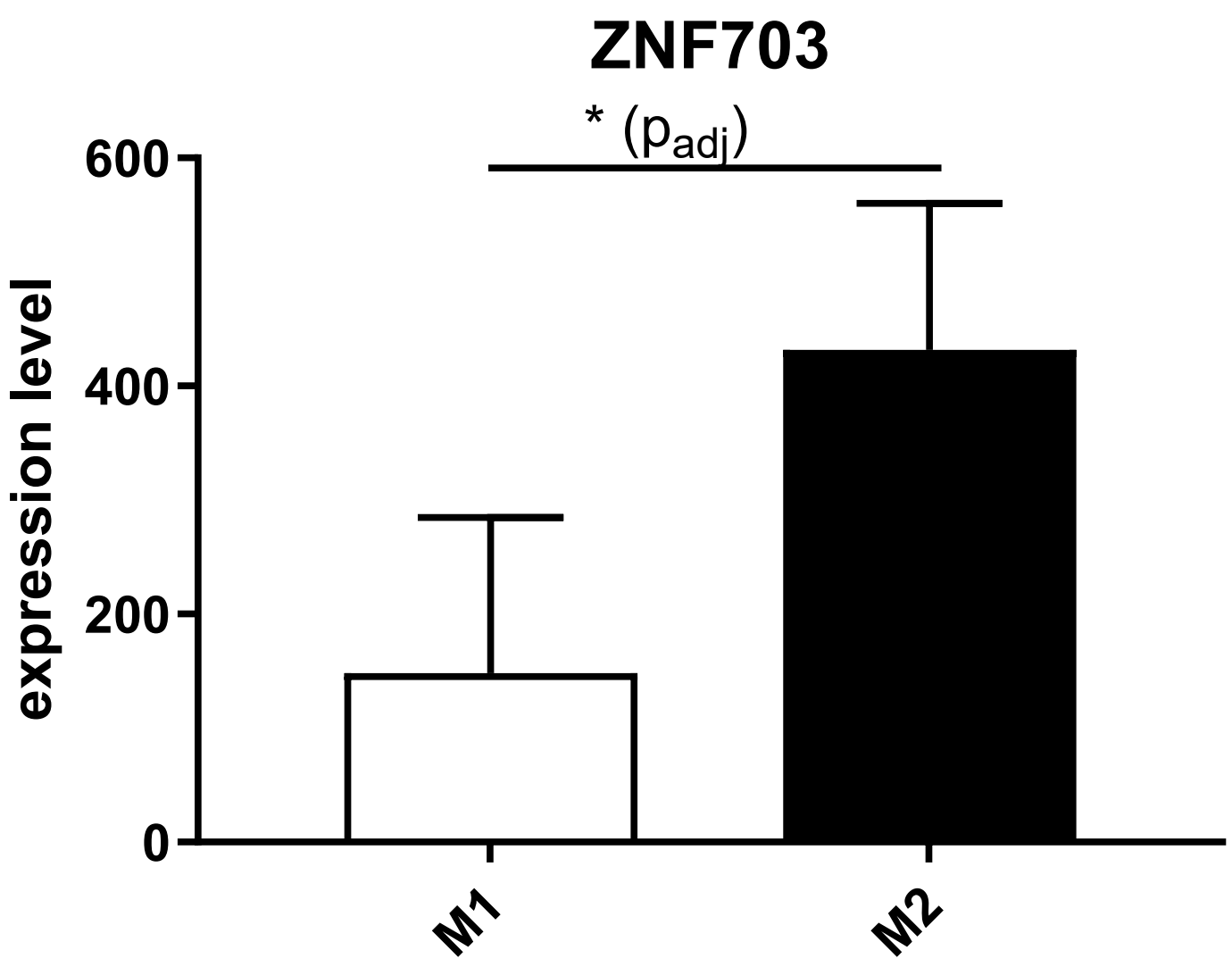

Fig. S13: Activity of transcription factors with increased (purple) or decreased (blue) activity compared to control miRNA-treated M2-like macrophages Based on microarray gene expression data from human macrophages polarized towards an M2-like state using IL-4 and subsequently transfected with either control miRNA (ath-miR-416) or pan-miRNAs (miR-155-5p, miR-3535, or miR-9-5p), we calculated the activity of 1160 transcription factors using our previously published TF activity model. Activity fold changes were computed relative to the control miRNA group. Shown are selected transcription factors with consistently increased or decreased activity across all pan-miRNA treatments.

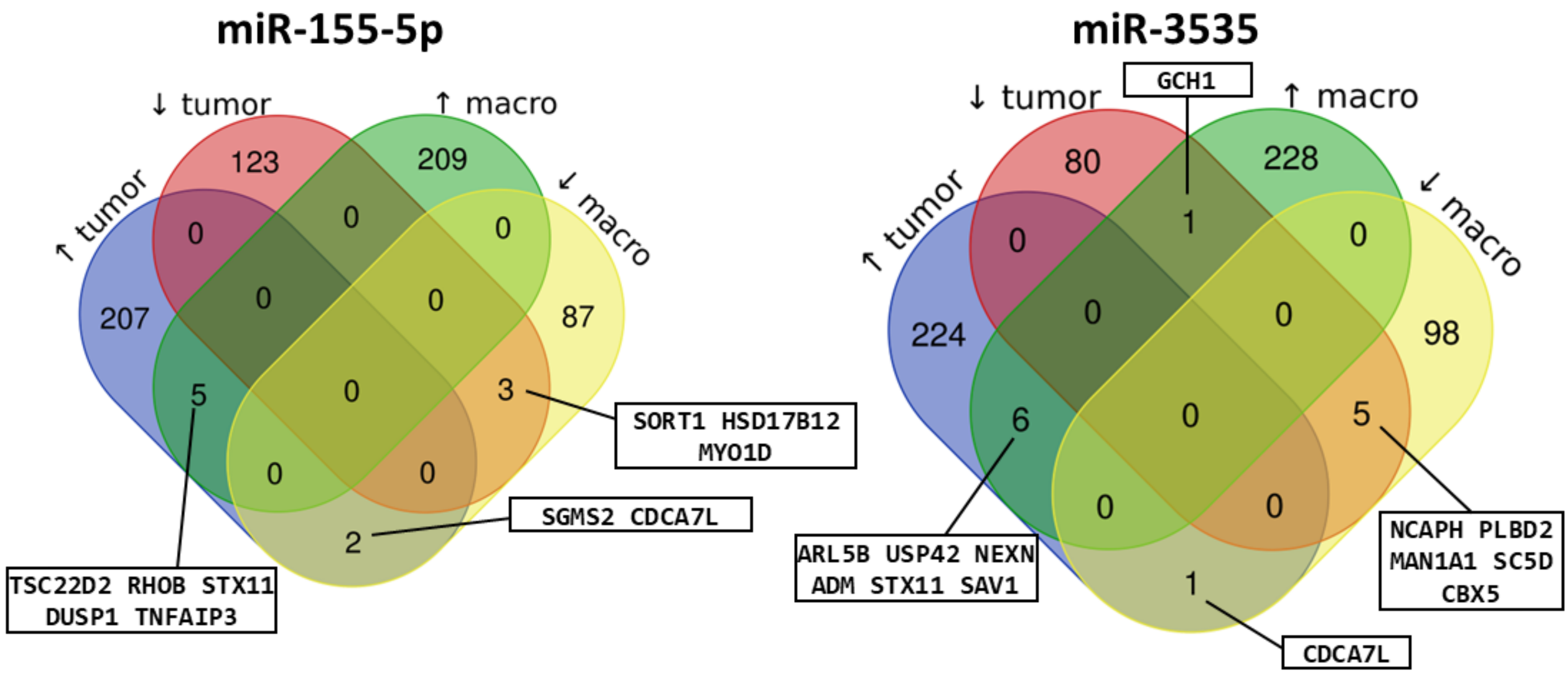

Fig. S14: Overlap of significantly downregulated or upregulated genes in M2-like polarized macrophages and tumor cells transfected with pan-functional miRNAs. Venn diagrams illustrate the overlap of genes significantly downregulated (FC < 0.5) or upregulated (FC > 2.0) in M2-like polarized human macrophages and the MDA-MB-231 breast cancer cell lines following transfection with miR-155-5p or miR-3535, compared to control miRNA (ath-miR-416). Only a limited number of transcripts were consistently regulated in both cell types, highlighting cell-type-specific responses to pan-functional miRNAs.

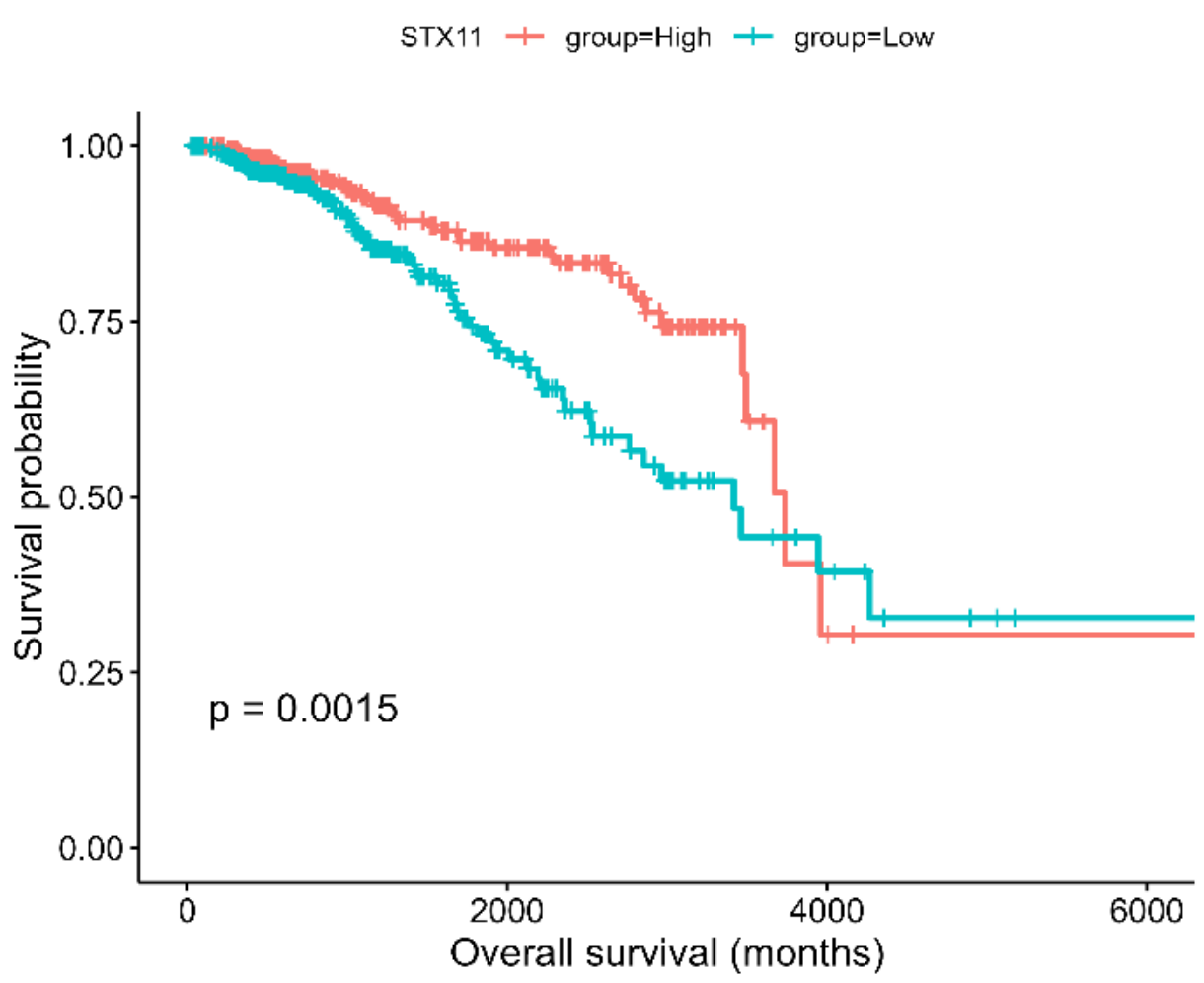

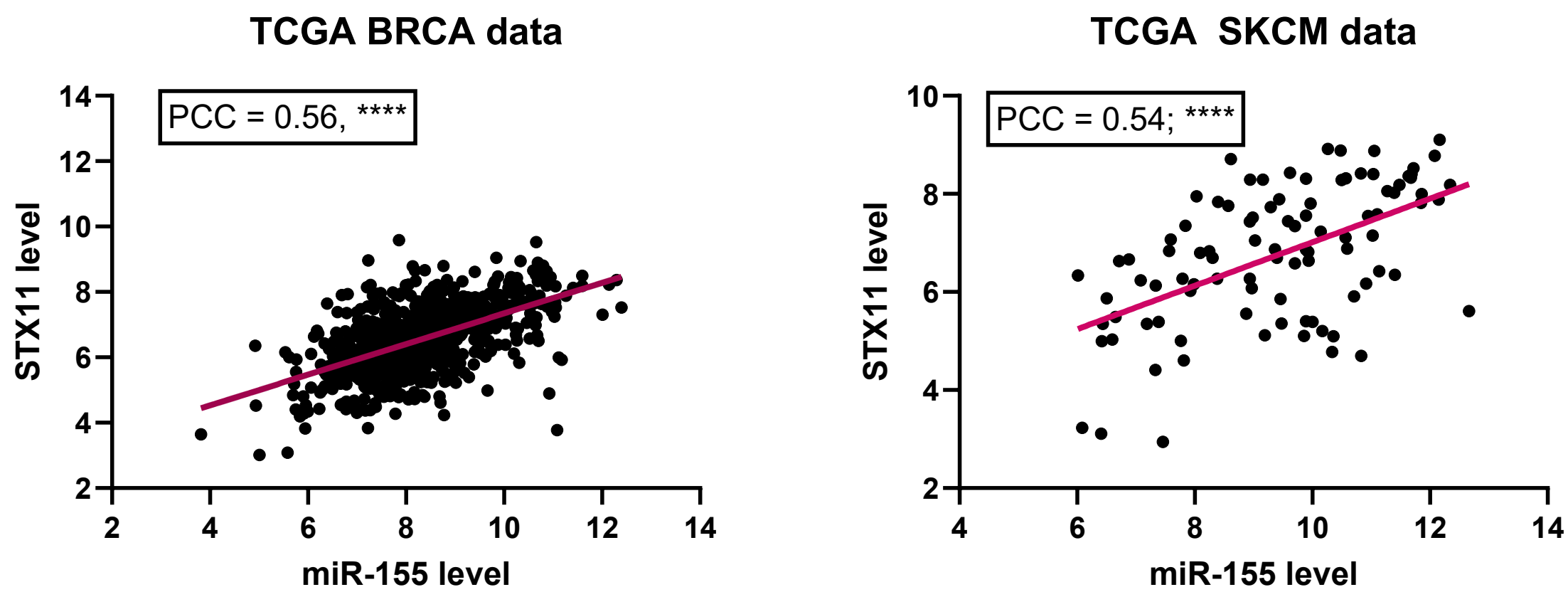

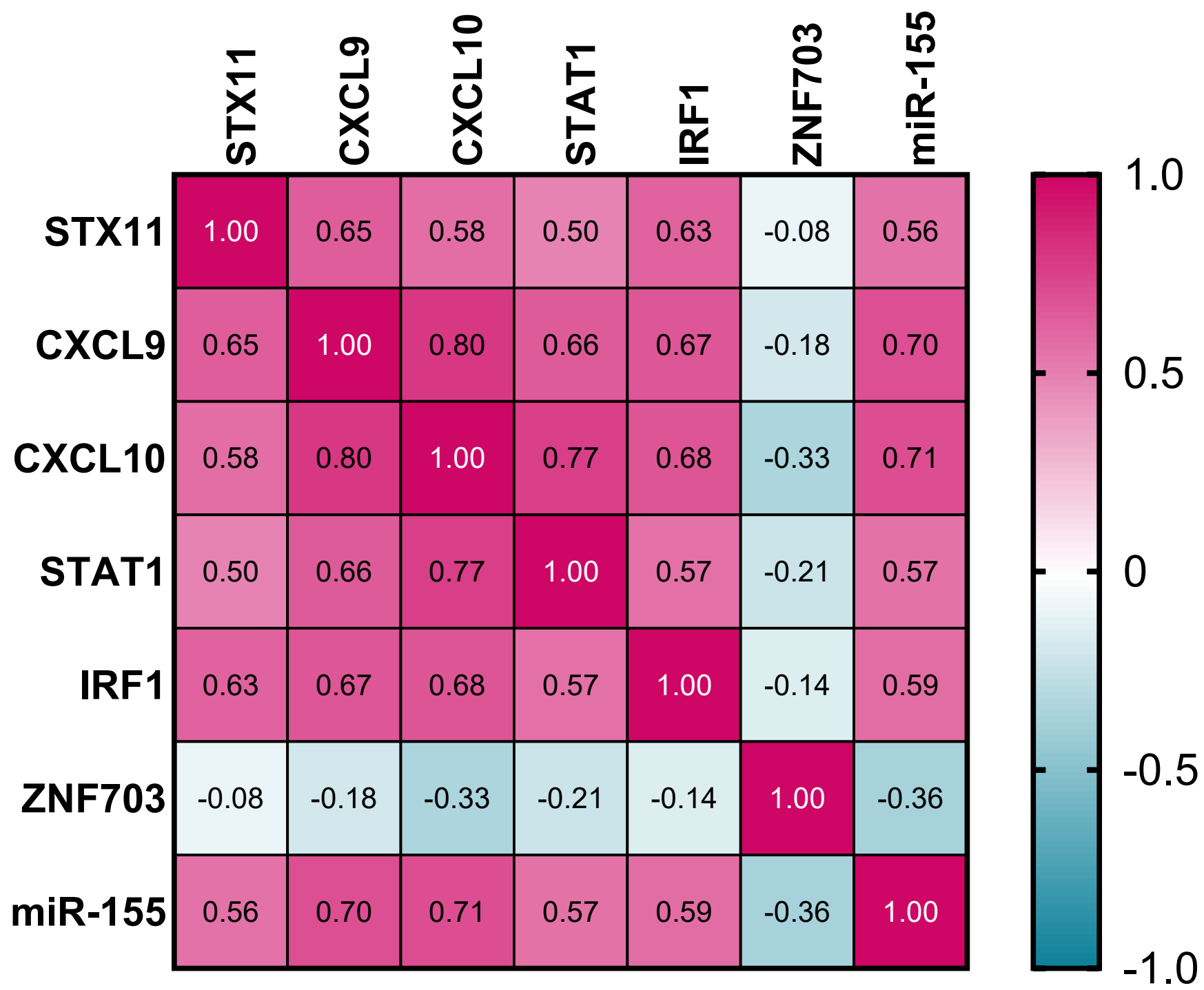

Fig. S15: STX11 expression and interferon-associated inflammatory gene signatures correlate with miR-155-5p in TCGA datasets. Kaplan–Meier analysis of overall survival in

TCGA breast cancer (TCGA-BRCA) stratified by STX11 mRNA expression (high vs low, median split). High STX11 expression was associated with improved overall survival. Statistical significance was assessed by log-rank test. Correlation between miR-155-5p expression and STX11 mRNA expression in TCGA-BRCA and TCGA melanoma (TCGA-SKCM). Correlation analyses were performed using Pearson correlation. PCC > 0.5 indicates a strong positive association. Significance levels: ****p < 0.0001. Correlation matrix showing associations between miR-155-5p and interferon-associated pro-inflammatory genes (GBP5, CXCL9, CXCL10, STAT1, IRF1, and STX11) in TCGA breast cancer. Strong positive correlations were observed among these genes, indicating a coordinated inflammatory transcriptional network. Correlation analysis of ZNF703 with miR-155-5p and the inflammatory gene set. ZNF703 showed consistent inverse correlations with miR-155-5p and interferon-associated genes, suggesting opposing regulatory programs.

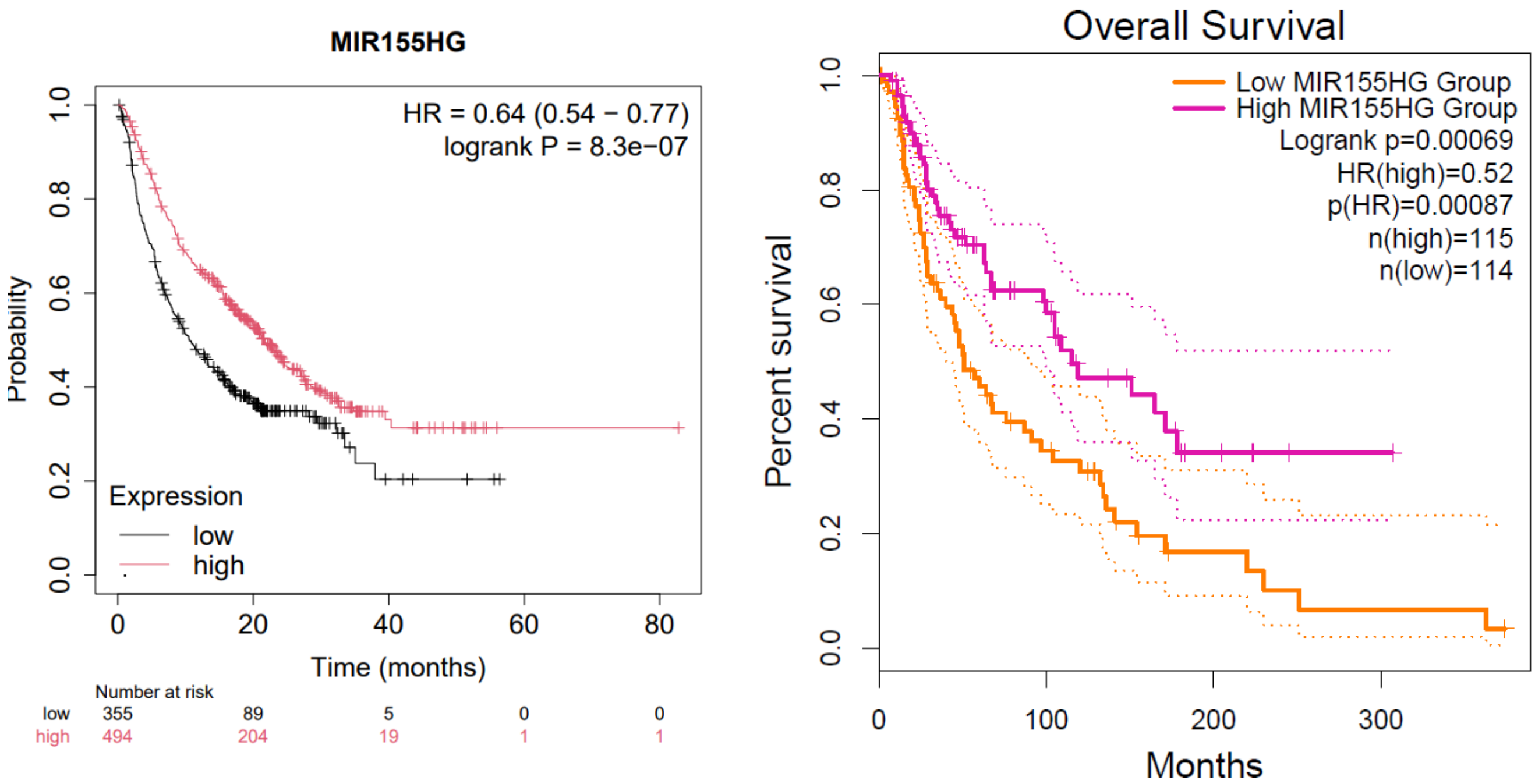

Fig. S16: High MIR155HG expression correlates with improved survival in cancer patients, including immunotherapy-treated and melanoma cohorts. High expression of MIR155HG is significantly associated with improved patient survival in cancer cohorts treated with immunotherapy (left), in melanoma (SKCM) patients, elevated MIR155HG levels correlate with better overall survival (right), supporting the role of miR-155-5p as a tumor-suppressive miRNA.

A

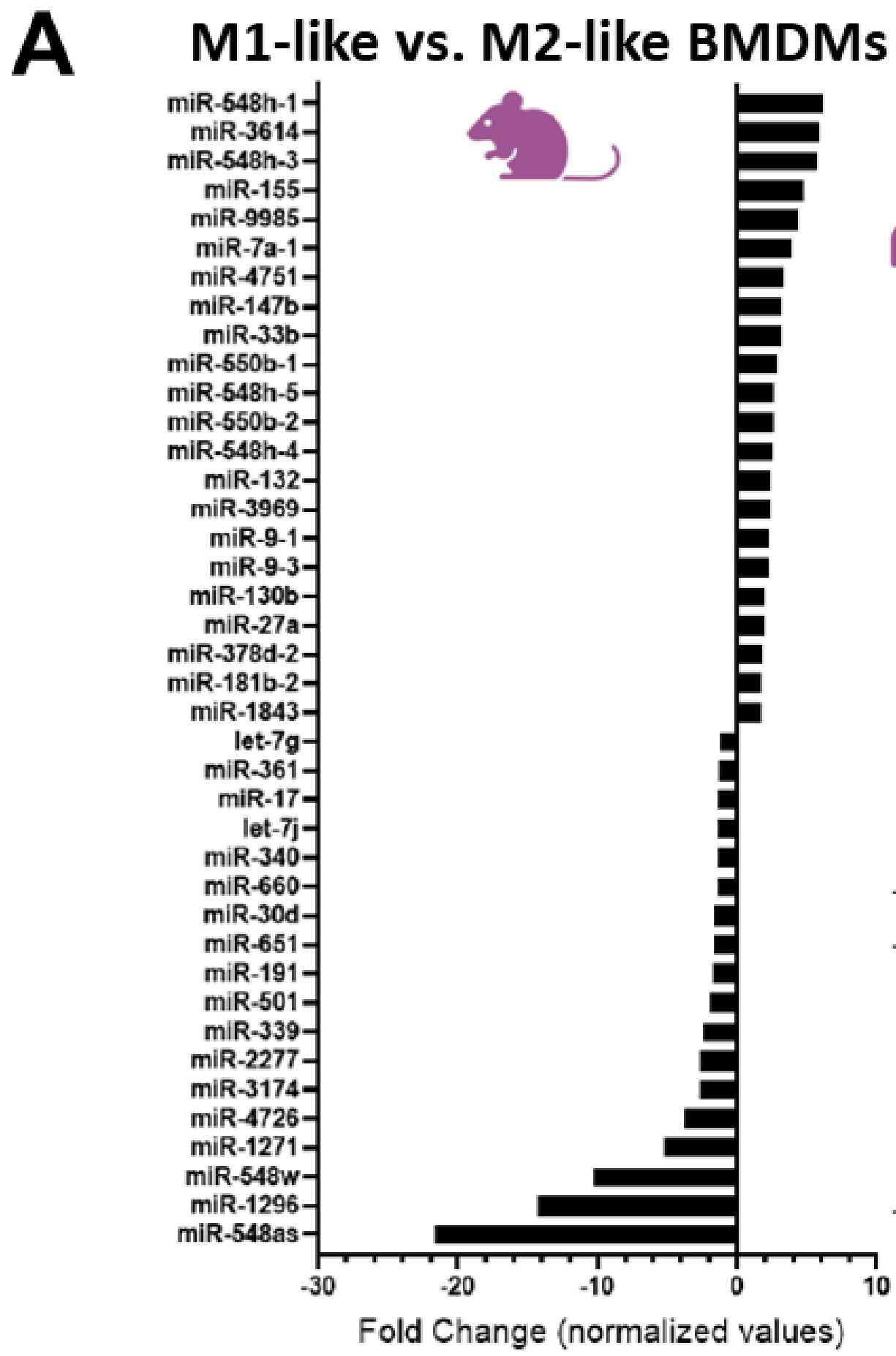

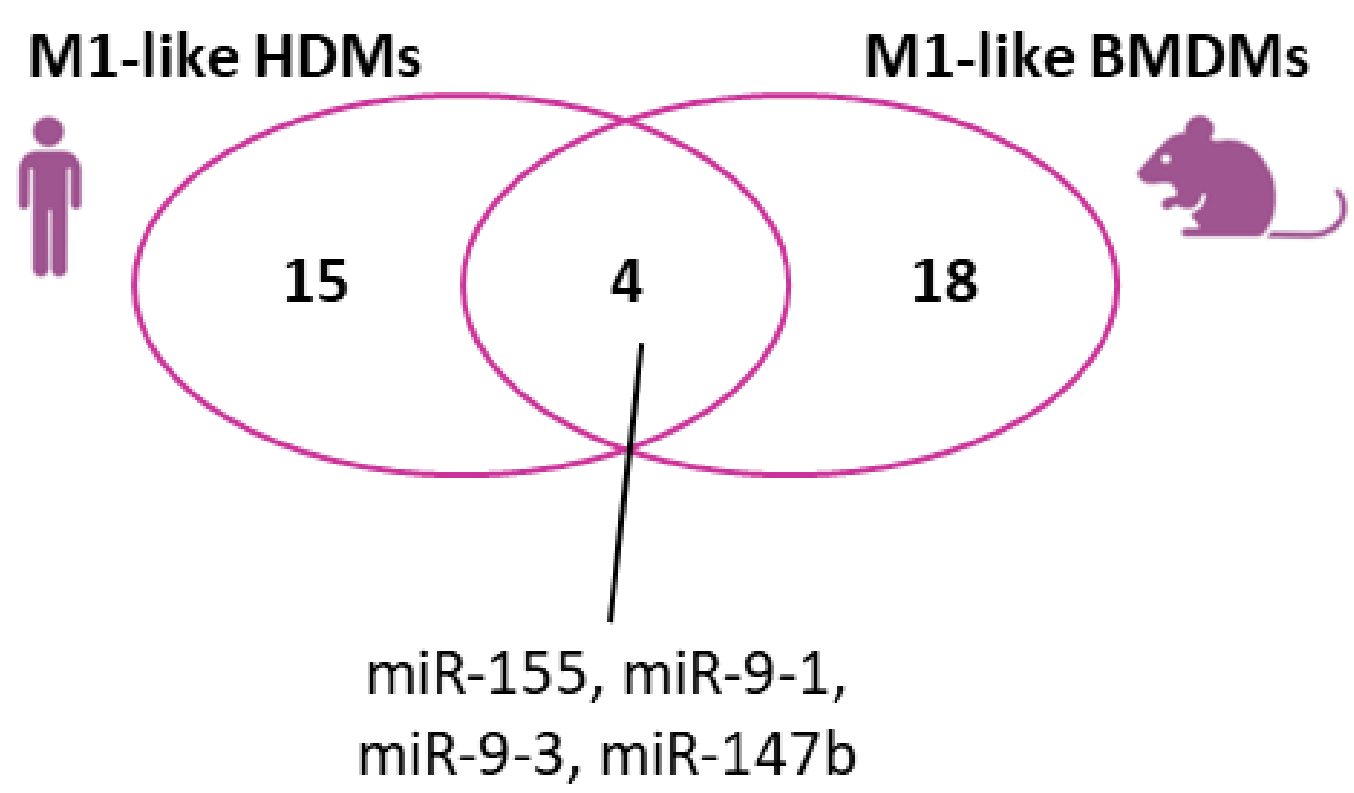

In silico **predictive binding scores of candidate miRNAs to key transcriptional regulators in macrophage polarisation.**

miRNA	Sum	*Hdac1*	*Hdac3*	*Myc*	*Stat6*	*Pparg*
mmu-miR-709	10	0	1	5	2	0
mmu-miR-694	10	5	0	4	0	0
mmu-miR-144-3p	9	0	5	0	3	4
mmu-miR-125a-5p	9	0	4	0	1	0
mmu-miR-125b-5p	9	0	4	0	1	0
mmu-miR-155-5p	0	0	0	0	0	0
mmu-miR-9-5p	7	1	1	1	2	0

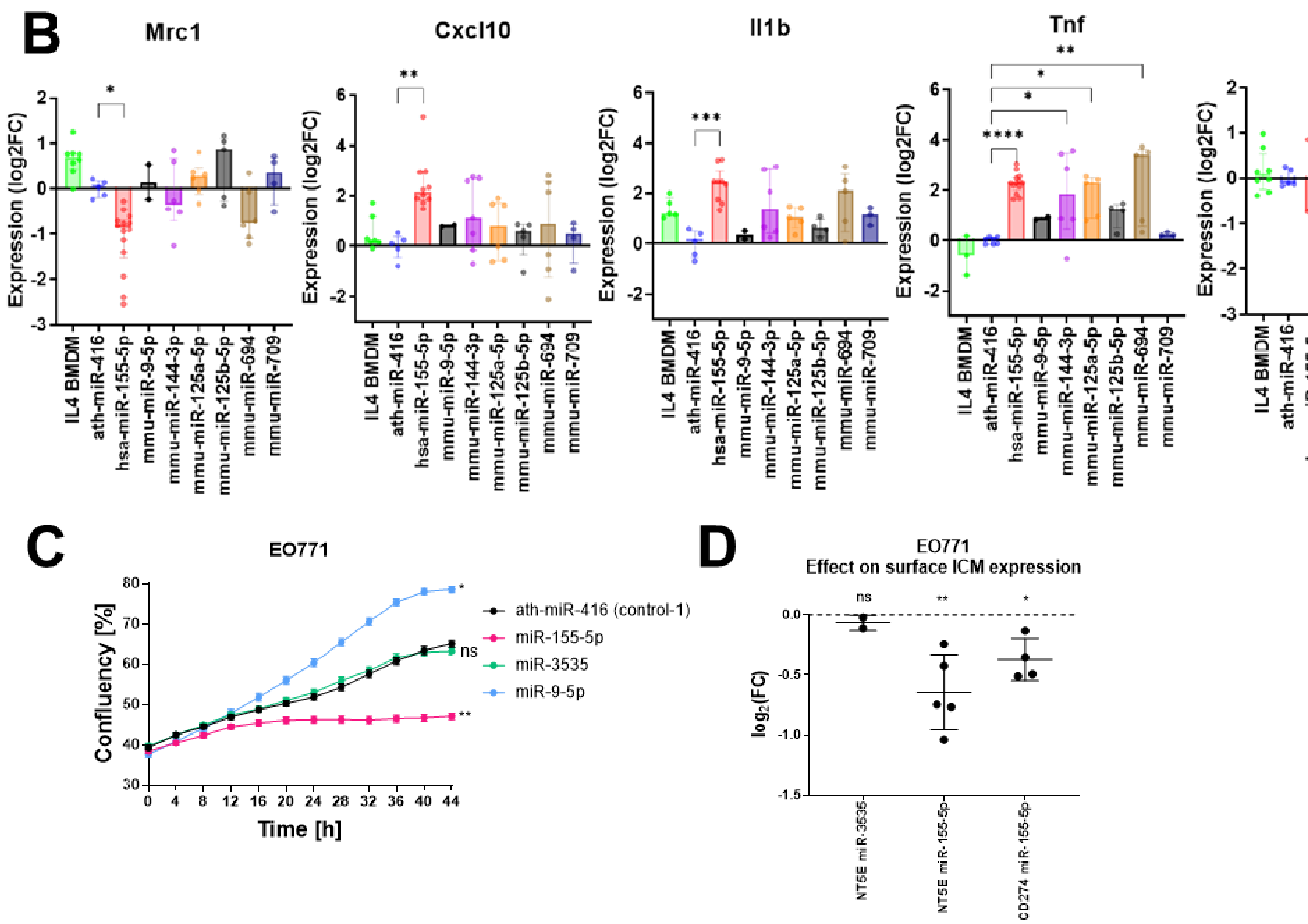

Fig. S17: Cross-species validation of miR-155-5p–mediated effects in murine macrophages and tumor cells. (A) RNA s analysis of murine bone marrow-derived macrophages (BMDMs) polarized toward an M1-like phenotype (LPS + IFNγ) or an M2-like phenotype (IL-4). miR-155-5p is significantly enriched in M1-like compared to M2-like macrophages. Comparative cross-species analysis revealed that four miRNAs (miR-155, miR-9-1, miR-9-3, and miR-147b) were commonly upregulated in both human M1-like HMDMs and murine M1-like BMDMs. In contrast, no overlap was observed for miRNAs enriched in M2-like macrophages between species. Notably, miR-3535 did not show significant differential expression in murine BMDMs. Candidate miRNAs for functional repolarization experiments were selected either based on conserved enrichment in M1-like macrophages (miR-155-5p, miR-9-5p) or by *in silico* prediction analyses indicating potential targeting of multiple M2-associated transcriptional regulators, including Hdac1, Hdac3, Myc, Stat6, Pparg, and Stat3. Interestingly, despite its strong experimental activity, miR-155-5p was not identified by the *in silico* prediction model, whereas miR-9-5p showed predicted interactions with several M2-associated regulators. **(B)** Functional evaluation of candidate miRNAs for macrophage repolarization in murine bone marrow-derived macrophages (BMDMs). Candidate miRNAs were selected based on RNA-sequencing and *in silico* target prediction analyses. Transfection of M2-like polarized BMDMs with miR-155-5p induced a pro-inflammatory transcriptional program characterized by increased expression of Cxcl10, Il1b, and Tnf, together with reduced Mrc1 expression, indicating repolarization toward an M1-like phenotype. In contrast, miR-9-5p did not induce consistent repolarization effects in murine BMDMs, suggesting species- or context-dependent differences in miRNA activity. **(C)** Effect of miRNAs on proliferation of the murine breast cancer cell line EO771. EO771 cells were seeded at 10,000 cells/well in 96-well plates and transfected 24 h later with 50 nM miR-155-5p, miR-9-5p, miR-3535, or control miRNA (ath-miR-416). Cell confluence was monitored by live-cell imaging using the IncuCyte SX5 system over 48 h with images acquired every 4 h. Confluence and proliferation kinetics were analyzed using IncuCyte 2020B software. For each condition, six replicate wells were analyzed. Data are shown as mean ± SEM. Statistical analysis of proliferation kinetics was performed using repeated-measures one-way ANOVA followed by Dunnett's multiple comparison test comparing miRNA-transfected conditions to control transfection. *$p < 0.05$; **$p < 0.01$. **(D)** Effect of transfection with 50 nM miRNA (miR-155-5p or miR-3535) in EO771 murine breast cancer cells. Surface expression of Nt5e and Cd274 was assessed by flow cytometry and compared to control transfection with ath-miR-416. Statistical significance was determined using a one-sample t-test (*$p < 0.05$, **$p < 0.01$). miR-155-5p significantly reduced Nt5e and Cd274 surface expression, whereas miR-3535 did not show a significant effect.

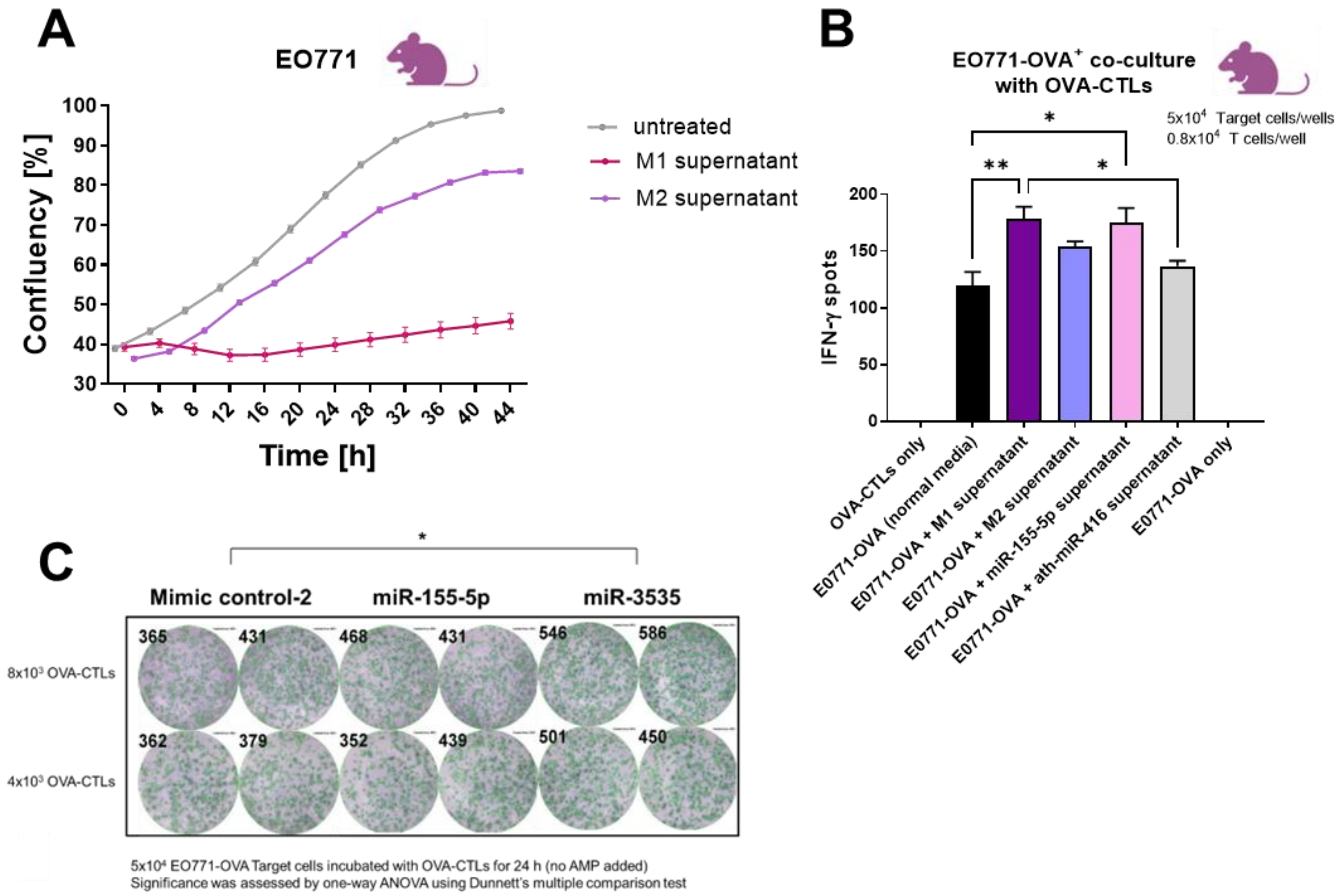

Fig. S18: Functional effects of macrophage polarization states on tumor cell proliferation and T-cell activation. (A) Effect of conditioned medium from polarized murine macrophages on EO771 breast cancer cell proliferation. EO771 cells were cultured in conditioned medium derived from M1-like macrophages (LPS + IFNγ), M2-like macrophages (IL-4), or untreated control medium. Cell proliferation was monitored by live-cell imaging using the IncuCyte SX5 system. Conditioned medium from M1-like macrophages significantly reduced EO771 proliferation compared to both untreated medium and conditioned medium from M2-like macrophages. Statistical analysis revealed significant effects of treatment, time, and treatment–time interaction (two-way ANOVA, all $p < 0.0001$). Tukey's multiple comparison test confirmed significantly reduced proliferation in EO771 cells cultured with M1-conditioned medium compared to M2-conditioned medium ($p < 0.0001$). **(B)** Functional assessment of T-cell activation in EO771-OVA/OVA-CTL co-culture systems in the presence of differently conditioned macrophage supernatants. OVA-specific cytotoxic T lymphocytes (OVA-CTLs) were co-cultured with EO771-OVA tumor cells in the presence of conditioned medium derived from classically polarized M1-like macrophages, M2-like macrophages, or macrophages transfected with the indicated miRNAs. IFNγ secretion was quantified by ELISpot assay. Conditioned medium from M1-like macrophages significantly enhanced IFNγ spot formation compared to normal medium controls ($p = 0.0098$). Similarly, conditioned medium from [miR-155-5p-transfected macrophages] significantly increased IFNγ spot numbers compared to normal medium controls ($p = 0.0133$). In contrast, conditioned medium from control-miRNA (ath-mIR-416) transfected macrophages did not significantly alter IFNγ secretion. Functional enhancement induced by miR-155-5p-conditioned macrophages was less pronounced than that observed following classical IFNγ+LPS-mediated M1 polarization, supporting the concept of partial macrophage repolarization following miRNA treatment. **(C)** Functional assessment of

tumor cell–mediated T-cell activation following miRNA transfection of EO771-OVA cells. EO771-OVA tumor cells (5 × 10^4 cells/well) were transfected with 50 nM miR-155-5p, miR-3535, or control miRNA (mimic control-2/cel-miR-39b-5p) and co-cultured with 8 × 10^3 OVA-specific cytotoxic T lymphocytes (OVA-CTLs). IFNγ secretion was quantified by ELISpot assay. miR-3535-transfected EO771-OVA cells induced significantly increased IFNγ spot formation compared to control-transfected tumor cells, whereas miR-155-5p did not show a significant effect. No AMP supplementation was added to the assay system, indicating that the observed effects were independent of exogenous adenosine generation and therefore unlikely to be solely mediated through altered NT5E/CD73-dependent AMP conversion.
